# Bi-allelic variants in the non-protein-coding minor spliceosome components *RNU6ATAC* and *RNU4ATAC* cause syndromic monogenic autoimmune diabetes

**DOI:** 10.1016/j.ajhg.2026.02.017

**Published:** 2026-03-20

**Authors:** Matthew B. Johnson, James Russ-Silsby, Paul A. Blair, Molly Govier, Georgia Bonfield, Clara Domingo-Vila, EXE-T1D consortium, ATAC clinical consortium, Matthew N. Wakeling, Richard A. Oram, Sarah E. Flanagan, Timothy I.M. Tree, Kashyap A. Patel, Andrew T. Hattersley, Elisa De Franco

**Affiliations:** 1 Clinical and Biomedical Science, Faculty of Health and Life Sciences, University of Exeter, Exeter, UK; 2 Department of Immunobiology, King’s College London, London, UK

**Keywords:** small nuclear RNA, non-coding, minor spliceosome, monogenic diabetes

## Abstract

Non-protein-coding genes are emerging as critical contributors to the etiology of rare diseases, providing key insights into human biology and uncovering novel disease mechanisms. We identified 7 individuals from 4 families with early-onset diabetes (diagnosed aged <5 years) and immune dysregulatory features caused by bi-allelic variants in *RNU6ATAC*. *RNU6ATAC* encodes a small nuclear RNA (snRNA) that acts as a catalytic component of the minor spliceosome, a protein-RNA complex that mediates the splicing of ∼700 genes containing U12/minor-type introns. Variant screening of the other 64 minor spliceosome genes in 276 infants with diabetes identified 12 unrelated individuals with bi-allelic disease-causing variants in *RNU4ATAC*. Bi-allelic pathogenic *RNU4ATAC* variants are known to cause a variable spectrum of clinical features, which until now did not include diabetes. Clinically, 12/19 *RNU6ATAC/RNU4ATAC* affected individuals had additional immune dysregulatory features, and 50% of individuals tested were islet-autoantibody positive, strongly supporting an autoimmune etiology for their diabetes. RNA sequencing (RNA-seq) in 3 individuals with bi-allelic *RNU6ATAC* variants showed a pattern of intron retention in U12-intron-containing genes similar to that seen in *RNU4ATAC* individuals (*n* = 3), supporting a shared disease mechanism. Analysis of affected individuals’ transcriptomic, methylation, and immune data revealed impaired B cell development and maturation. We conclude that bi-allelic *RNU6ATAC* variants cause a syndrome of early-onset autoimmune diabetes and immune dysregulation. We further show that infancy-onset diabetes is a feature of RNU4ATAC-opathy. Our work highlights the important role of two snRNAs critical to minor spliceosome function in immune system regulation, providing insights into the pathogenesis of autoimmune diabetes.

## Graphical abstract

**Figure:**
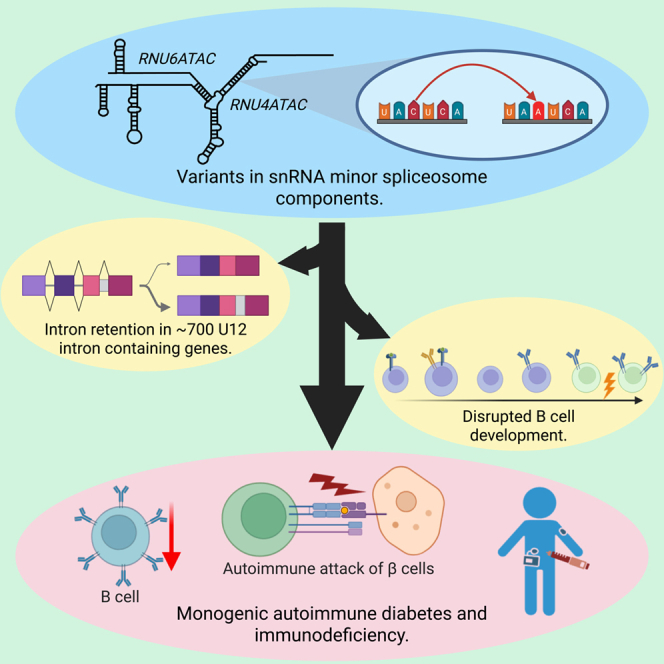

## Teaser

We show that bi-allelic variants in the minor spliceosome snRNAs *RNU6ATAC* and *RNU4ATAC* cause early-onset autoimmune diabetes with immune dysregulation. We define a shared mechanism involving U12 intron retention and impaired B cell development, expanding the phenotypic spectrum of RNU4ATAC-opathy and implicating minor splicing in beta cell autoimmunity.

## Main text

Uncovering genetic causes of human disease provides key insights into the regulation of core biological processes. Despite significant advances ever since the introduction of exome and genome sequencing, up to half of individuals affected by a rare disease remain without a genetic diagnosis.^1^^,^^2^ Variants in the non-protein-coding genome—including regulatory elements such as promoters, enhancers, and untranslated regions—may account for a considerable fraction of this diagnostic gap.^3^^,^^4^^,^^5^ The recent discovery that ReNU syndrome (MIM: 620851)—caused by variants in the non-protein-coding gene *RNU4-2* (MIM: 620823)—explains ∼0.4% of individuals with undiagnosed neurodevelopmental delay has highlighted the importance of non-protein-coding genes in rare disease.^6^^,^^7^^,^^8^

Here, we report the identification of variants in the non-protein-coding gene *RNU6ATAC* (MIM: 601429), encoding a catalytic component of the minor spliceosome, as the genetic cause of a syndrome defined by early-onset autoimmune diabetes, hypogammaglobulinemia, and additional immune dysregulation. Through screening of the genes encoding the other minor spliceosome components, we also extend the phenotype associated with bi-allelic variants in another non-coding minor spliceosome component, *RNU4ATAC* (MIM: 601428), to include early-onset autoimmune diabetes.

**Figure 1:**
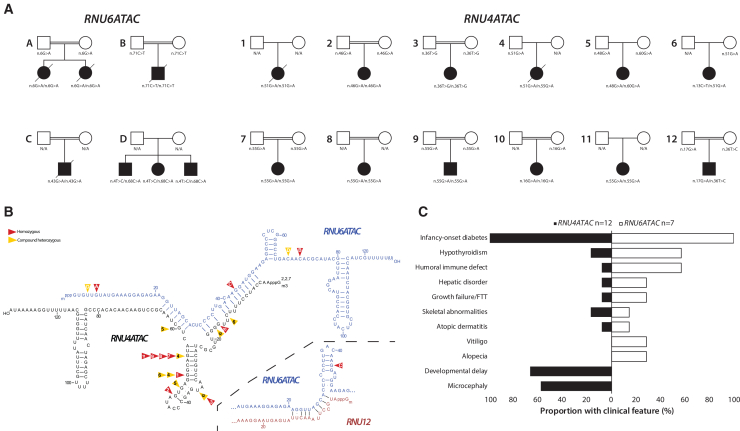
Genetic and clinical information for the RNU6ATAC and RNU4ATAC cohorts (A) Partial pedigrees of the individuals with *RNU6ATAC* and *RNU4ATAC* bi-allelic variants. (B) Secondary structure of RNU4ATAC and RNU6ATAC snRNA duplex with variant positions highlighted and number/letter by the affected individual. Heterozygous variants identified in *trans* with another pathogenic variant are shown with yellow arrows. Homozygous variants are shown with red arrows. Hydrogen bonds between bases in stem I and stem II are shown with lines. The image was adapted from Almentina Ramos Shidi et al.^11^ (C) Tornado plot showing clinical features in *RNU6ATAC* vs. *RNU4ATAC* cohorts.

To identify genetic causes of autoimmune diabetes and immune dysregulation, we performed genome sequencing in three consanguineous individuals with infancy-onset diabetes (diagnosed at 13, 17, and 36 weeks) and hypogammaglobulinemia. All known genetic causes of infancy-onset diabetes had been previously excluded. No shared genes with ultra-rare homozygous coding variants (gnomAD v.4.1.0^9^ minor-allele frequency [MAF] < 1 × 10^−5^) in all 3 individuals were identified. We next looked for ultra-rare homozygous variants in non-coding genes. This identified a single common gene, the small nuclear RNA (snRNA) *RNU6ATAC* (Figure 1A; Table 1). All three individuals had a different *RNU6ATAC* homozygous variant that was absent from gnomAD v.4.1.0 (∼75,000 individuals) and ClinVar.^10^ The affected sibling of one of the individuals (individual A.II-2) was also homozygous for the *RNU6ATAC* rare variant. *RNU6ATAC* variants have not previously been reported as causing human monogenic disease.

**Table 1:** Demographic, genetic, and clinical features of individuals with bi-allelic *RNU6ATAC* disease-causing variants

**Individual**	**A.II-1**	**A.II-2**	**B.II-1**	**C.II-1**	**D.II-1**	**D.II-2**	**D.II-3**
Age at last assessment (years^a^)	0.3 (deceased)	0.3 (deceased)	0.3 (deceased)	5	4	14	9
Sex	female	female	male	male	male	female	male
Genetic ancestry	EAS	EAS	SAS	MID	MID	MID	MID
BW *Z* score	−2.26	−3.17	−1.72	N/R	0.06	0.06	N/R
Age diagnosed with diabetes (weeks)	13	11	17	36	5	110	260
Glucose at diagnosis (mmol/L)	28.3	33	41	N/R	25	25	N/R
HbA1c (mmol/mol)	N/A^b^	N/A^b^	N/A^b^	N/R	111	108	118
Insulin dose U/kg/day	N/A, on infusion before death	N/A, on infusion before death	1.5	N/R	0.7	N/R	N/R
Islet autoantibodies (titer, threshold, IU)	N/A	N/A	GADA positive (52, >11)	N/R	negative	negative	GADA positive (98, >11)
Immune dysregulatory features	sepsis, atopic dermatitis, B cell lymphopenia, low IgA, low IgG, hypothyroidism	B cell lymphopenia, hypothyroidism	agammaglobulinemia	hypogammaglobulinemia, immunodeficiency	thyroiditis (1.5 years), alopecia (3.5 years)	N/R	alopecia and vitiligo (4.5 years), thyroiditis (4 years)
Microcephaly	no	no	no	N/R	no	no	no
Developmental delay	N/A, died early infancy	N/A, died early infancy	NA, died early infancy	N/R	no	no	no
Additional features	diarrhea, facial dysmorphism	jaundice, elevated unconjugated bilirubin	none reported	epiphyseal dysplasia, elevated liver enzymes	none reported	severe growth retardation (not GH deficient), delayed puberty	none reported
*RNU6ATAC* variant	n.[6G>A]; [6G>A] (GenBank: NR_023344.1)	n.[6G>A]; [6G>A] (GenBank: NR_023344.1)	n.[71C>T]; [71C>T] (GenBank: NR_023344.1)	n.[43G>A]; [43G>A] (GenBank: NR_023344.1)	n.[4T>C]; [68C>A] (GenBank: NR_023344.1)	n.[4T>C]; [68C>A] (GenBank: NR_023344.1)	n.[4T>C]; [68C>A] (GenBank: NR_023344.1)
Genomic co-ordinate (Hg38)	g.[134164559C>T]; [134164559C>T] (GenBank: NC_000009.12)	g.[134164559C>T]; [134164559C>T] (GenBank: NC_000009.12)	g.[134164494G>A]; [134164494 G>A] (GenBank: NC_000009.12)	g.[134164522C>T]; [134164522C>T] (GenBank: NC_000009.12)	g.[134164561A>G]; [134164497G>T] [GenBank: NC_000009.12]	g.[134164561A>G]; [134164497G>T] [GenBank: NC_000009.12]	g.[134164561A>G]; [134164497G>T] [GenBank: NC_000009.12]

BW, birthweight; N/A, not applicable; N/R, not recorded; EAS, East Asian; SAS, South Asian; MID, Middle Eastern; F, female; M, male; GADA, glutamic acid decarboxylase; GH, growth hormone.

a Presented as years and fractions of a year.

b Due to the presence of fetal hemoglobin, HbA1c measurement is not reliable in the first 6 months of life.

To identify further individuals, we screened existing genome sequencing data from 276 individuals with neonatal diabetes (diagnosed aged <6 months) or early-onset diabetes with additional autoimmune disease (both diagnosed aged <5 years) in whom known causes of monogenic diabetes had been excluded.^12^ This analysis identified 3 further individuals from 1 family with compound heterozygous variants in *RNU6ATAC* (Figure 1A; Table 1), taking the total to 7 individuals from 4 families. Sanger sequencing of *RNU6ATAC* (see the supplemental material and methods for primer sequences and amplified region) in 196 individuals with neonatal diabetes of unknown cause who did not have genome sequencing data did not identify any additional individuals.

*RNU6ATAC* forms part of the catalytic core of the minor spliceosome.^13^^,^^14^ This fundamental and highly evolutionarily conserved protein-RNA complex mediates the excision of introns containing the U12-type sequence motif (<0.5% of all human introns) to create mature mRNA.^15^ Disruption of the spliceosome causes intron retention that leads to downstream impacts, including transcript loss due to nonsense-mediated decay or disruption to protein function through protein truncation or protein elongation.^16^

We next investigated if bi-allelic variants in the other 64 genes encoding components of the minor spliceosome were implicated in the etiology of early-onset diabetes (Table S1).^14^^,^^17^ We screened for ultra-rare variants in these genes using the same genome sequencing dataset used for the *RNU6ATAC* analysis described above. This identified 7 unrelated individuals with homozygous or compound heterozygous variants in *RNU4ATAC* (Figure 1A; Table 2). We did not identify ultra-rare bi-allelic variants in any of the 63 remaining genes. We then Sanger sequenced *RNU4ATAC* in 196 individuals with neonatal diabetes of unknown cause who had not undergone genome sequencing and identified 5 additional individuals. In total, we identified 12 individuals with bi-allelic likely pathogenic/pathogenic *RNU4ATAC* variants in our early-onset diabetes cohort.

**Table 2:** Demographic, genetic, and clinical features of individuals with bi-allelic *RNU4ATAC* disease-causing variants

**Individual**	**1.II-1**	**2.II-1**	**3.II-1**	**4.II-1**	**5.II-1**	**6.II-1**	**7.II-1**	**8.II-1**	**9.II-1**	**10.II-1**	**11.II-1**	**12.II-1**
Age at last assessment (years^a^)	6.8 (deceased)	12 (deceased)	0.9	1.4 (deceased)	8.7	11	5.9	9.5	1.0	0.8	1.3	0.4
Sex	F	F	F	F	F	F	F	F	F	F	F	M
Genetic ancestry	MID	OTH	MID	EAS	OTH	AFR	MID	OTH	EAS	MID	SAS	MID
BW *Z* score	−3.07	−3.04	−4.39	−4.29	−1.71	−0.97	1.14	−2.15	−1.56	−0.98	−0.14	−2.71
Age diagnosed with diabetes (weeks)	20	61	8	10	21	10	51	26	40	20	20	1
Glucose at diagnosis (mmol/L)	38.1	25	21	20.3	39	32	N/R	33	55	33	44	24
HbA1c (mmol/mol)	64	73	N/A	47.5	43.2	50	61	N/A	57	N/A	90	45
Insulin dose U/kg/day	1.5	3	0.82	1.5	0.8	0.72	N/R	1.2	1.0	1	1.4	0.5
Islet autoantibodies (titer, threshold for positivity)	negative	negative	N/A	negative	GADA positive (93, >11)	GADA positive (1,581, >11)	GADA positive (657, >11)	N/A	N/A	N/A	N/A	N/A
Immune dysregulatory features	recurrent infections, IgA deficiency	recurrent infections, myelodysplastic syndrome	N/R	N/R	raised immature granulocytes	severe atopic dermatitis, autoimmune hypothyroidism	N/R	recurrent infections	N/R	N/R	N/R	autoimmune hypothyroidism
Microcephaly	yes	yes	yes	yes	N/R	yes	yes	yes	yes	N/R	yes	N/R
Developmental delay	yes	yes	N/R	yes	yes	yes	yes	yes	N/R	N/R	yes	N/R
Additional clinical features	hip dislocation, muscle weakness, respiratory failure, seizures	triple X syndrome, insulin resistance, muscle weakness, dysmorphic features, growth retardation, diabetic nephropathy	facial dysmorphism, peaked nose, small head, downward slanting palpebral fissures, small mandible	muscle weakness, corpus callosum agenesis, bilateral knee dislocation	muscle weakness, epilepsy, cholestasis and direct hyperbilirubinemia (resolved), ASD, congenital cataracts, sensorineural hearing loss	loose skin folds, laryngomalacia tracheomalacia, multiple epiphyseal dysplasia, high myopia, alternating exotropia	high arched palate, abnormal object eye tracking, dysmorphic features	epilepsy, musculoskeletal abnormalities, premature menarche (9 years, suppressed), mitral valve prolapse; older sister affected with microcephaly and diabetes (11 m), died age 15 m	N/R	N/R	dysmorphism	muscle weakness
*RNU4ATAC* variant	n.[51G>A]; [51G>A] (GenBank: NR_023343.1)	n.[46G>A]; [46G>A] (GenBank: NR_023343.1)	n.[36T>G]; [36T>G] (GenBank: NR_023343.1)	n.[51G>A]; [55G>A] (GenBank: NR_023343.1)	n.[48G>A]; [60G>A] (GenBank: NR_023343.1)	n.[13C>T]; [51G>A] (GenBank: NR_023343.1)	n.[55G>A]; [55G>A] (GenBank: NR_023343.1)	n.[55G>A]; [55G>A] (GenBank: NR_023343.1)	n.[55G>A]; [55G>A] (GenBank: NR_023343.1)	n.[16G>A]; [16G>A] (GenBank: NR_023343.1)	n.[55G>A]; [55G>A] (GenBank: NR_023343.1)	n.[17G>A]; [36T>C] (GenBank: NR_023343.1)
Genomic co-ordinate (Hg38)	g.[121530930G>A]; [121530930G>A] (GenBank: NC_000002.12)	g.[121530925G>A]; [121530925G>A] (GenBank: NC_000002.12)	g.[121530915T>G]; [121530915T>G] (GenBank: NC_000002.12)	g.[121530930G>A]; [121530934G>A] (GenBank: NC_000002.12)	g.[121530927G>A]; [121530939G>A] (GenBank: NC_000002.12)	g.[121530892C>T]; [121530930G>A] (GenBank: NC_000002.12)	g.[121530934G>A]; [121530934G>A] (GenBank: NC_000002.12)	g.[121530934G>A]; [121530934G>A] (GenBank: NC_000002.12)	g.[121530934G>A]; [121530934G>A] (GenBank: NC_000002.12)	g.[121530895G>A]; [121530895G>A] (GenBank: NC_000002.12)	g.[ 121530934G>A]; [ 121530934G>A] (GenBank: NC_000002.12)	g.[121530896G>A]; [121530915T>C] (GenBank: NC_000002.12)

BW, birthweight; N/A, not applicable; N/R, not recorded; MID, Middle Eastern; OTH, other; EAS, East Asian; AFR, African; SAS, South East Asian; ASD, atrial septal defect; F, female; M, male; GADA, glutamic acid decarboxylase autoantibody.

a Presented as years and fractions of a year.

*RNU4ATAC* encodes an snRNA that binds directly to *RNU6ATAC* to stabilize the pre-catalytic configuration of the minor spliceosome, sequestering *RNU6ATAC*’s catalytic elements.^13^^,^^14^ Recessively inherited variants in *RNU4ATAC* are a known cause of “RNU4ATAC-opathies,”^18^ encompassing a highly variable clinical spectrum that commonly includes microcephaly, developmental delay, and intra-uterine growth restriction (IUGR) and can include immune dysregulatory features. Diabetes has not been reported as a feature of RNU4ATAC-opathies.

In total, we therefore identified 19 individuals from 16 families with defects in 2 minor spliceosome snRNAs and early-onset diabetes (Figures 1A and 1B): 7 individuals from 4 families with bi-allelic *RNU6ATAC* variants (Table 1, denoted by letters) and 12 unrelated individuals with bi-allelic *RNU4ATAC* variants (Table 2, denoted by numbers). Variant testing confirmed carrier status in all the parents available for testing (*n* = 19).

Bi-allelic *RNU6ATAC* variants caused early-onset diabetes (median onset: 17 weeks) with additional immune dysregulation in 6/7 individuals (Table 1). Common features included humoral immune defects (4/7; B cell lymphopenia and a/hypogammaglobulinemia) and autoimmunity (4/7; hypothyroidism [*n* = 4], alopecia [*n* = 2], and vitiligo [*n* = 1]). Cholestatic jaundice and elevated liver enzymes were observed in two individuals. Among individuals with reported gestation, 3/5 had IUGR (*Z* score < −1.28^19^).

Individuals with bi-allelic *RNU4ATAC* variants often displayed classic RNU4ATAC-opathy features,^18^ including microcephaly, IUGR, and developmental delay, which were seen in 10/12 individuals (Table 2). Six had immune dysregulation (including hypothyroidism [*n* = 2] and recurrent infections [*n* = 3]). Autoimmunity (e.g., Addison’s disease and autoimmune hypothyroidism) has been reported in some individuals with RNU4ATAC-opthies,^20^ including a single individual with type 1 diabetes (T1D) and Addison’s disease.^21^ In our cohort, 12 unrelated individuals had early-onset diabetes (median onset: 20 weeks), confirming that diabetes is part of the RNU4ATAC-opathy spectrum. Diabetes is unlikely to be attributable to specific variants in *RNU4ATAC*, as 12/15 variants were previously reported to cause RNU4ATAC-opathies in individuals who were not diagnosed with diabetes.^10^

There was substantial overlap in clinical features between individuals with variants in *RNU6ATAC* and *RNU4ATAC* but also some notable differences (Figure 1C). In our cohort, variants in both genes were associated with early-onset diabetes and immune dysregulation. Developmental abnormalities were common in individuals with *RNU4ATAC* variants, consistent with previous reports, but not in those with variants in *RNU6ATAC*, although later-onset developmental issues in the latter group cannot be excluded. A recent study identified an individual with compound heterozygous ultra-rare *RNU6ATAC* variants of unknown clinical significance (n.[36T>G]; [28C>T] [GenBank: NR_023344.1]) who had IUGR, postnatal growth failure, microcephaly, epilepsy, intellectual disability, and ataxia but no diabetes.^22^

To investigate the diabetes mechanism, we first performed islet autoantibody testing on available serum samples (*n* = 4 *RNU6ATAC* and *n* = 6 *RNU4ATAC*). Five of the ten individuals (50%) were positive for GADA (glutamic acid decarboxylase) antibodies (threshold is >97.5^th^ centile of the non-diabetic population). This rate of positive islet autoantibodies is similar to that seen in age-matched individuals with T1D and monogenic autoimmune diabetes,^23^^,^^24^ indicating that the diabetes in these individuals is likely driven by islet autoimmunity. Furthermore, all individuals with available clinical data presented in infancy/early-childhood with very high glucose values (median: 32 mmol/L, interquartile range [IQR]: 25–39, *n* = 15, Table 1) and were insulin treated with full replacement doses (median: 1.0 U/kg/day, IQR: 0.7–1.5, *n* = 13, Table 1), indicative of profound and rapid loss of endogenous insulin secretion.

**Figure 2:**
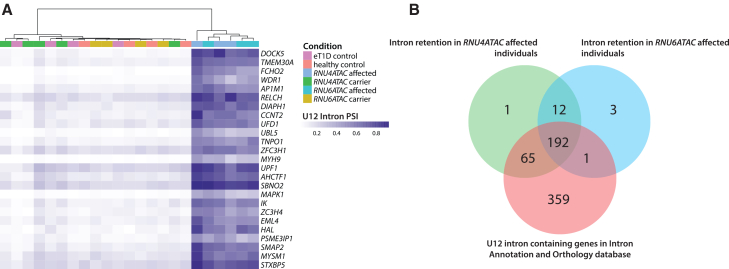
RNA-seq identifies significant intron retention in individuals with variants in *RNU6ATAC* and *RNU4ATAC* (A) Heatmap showing top 25 genes with significant intron retention. Individuals with variants in *RNU4ATAC* and *RNU6ATAC* cluster together, showing similar profiles of intron retention across U12-intron-containing genes. PSI, percent spliced in. (B) Venn diagram showing the intersection of known U12-intron-containing genes (IAOD^25^; pink), and empirically defined genes with significant intron retention in whole-blood RNA from individuals with variants in *RNU6ATAC* (*n* = 3, blue) and individuals with variants in *RNU4ATAC* (*n* = 3, green). Thirteen genes were identified that showed significant intron retention in both cohorts but were not present in the IAOD database, most likely reflecting previously undescribed U12 genes.

To assess the impact of the identified *RNU6ATAC* variants, we performed whole-blood RNA sequencing (RNA-seq) on samples from 3 individuals with bi-allelic variants and compared intron retention to our individuals with bi-allelic *RNU4ATAC* variants (*n* = 3), unaffected parents (*n* = 4 *RNU6ATAC* and 5 *RNU4ATAC*), healthy age-matched control subjects (*n* = 4), and age-matched individuals with early-onset T1D (*n* = 4) (Table S2). This identified significant intron retention in individuals with bi-allelic *RNU6ATAC* variants, similar to the pattern detected in individuals with bi-allelic *RNU4ATAC* variants, in 274 genes (Figure 2A). Most genes with significant intron retention (*n* = 258/274, 94%) were known U12-intron-containing genes listed in the Intron Annotation and Orthology U12 Database (IAOD) (Figure 2B; Table S3).^25^ The remaining 16 genes likely represent previously undescribed U12 genes, given we found significant intron retention in individuals with bi-allelic variants in *RNU4ATAC* (*n* = 1), *RNU6ATAC* (*n* = 3), or both (*n* = 12) (Figures 2A and S1; Table S3). The high level of intron retention of U12 genes identified in our cohort was comparable to that previously reported in individuals with RNU4ATAC-opthies.^20^^,^^26^^,^^27^

**Figure 3:**
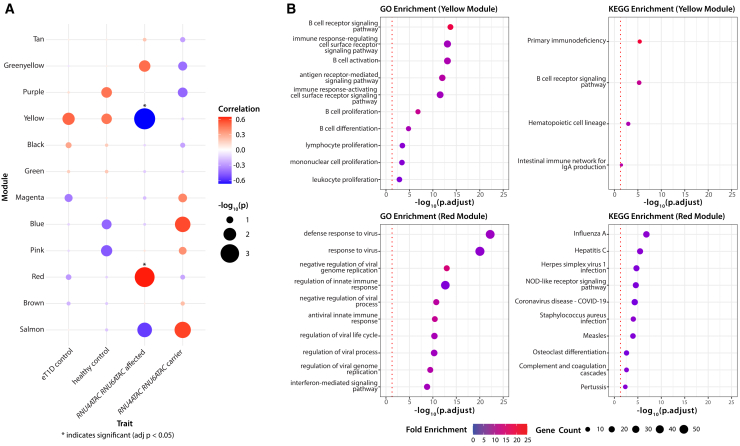
Weighted gene co-expression network analysis and gene enrichment analysis of resulting enriched gene modules from disease cohort RNA-seq data (A) Correlation between weighted gene co-expression network analysis (WGCNA) and derived gene modules and disease status, which included affected individuals with *RNU6ATAC* and *RNU4ATAC* variants, carriers, age-matched T1D control subjects, and age-matched healthy control subjects. Colors are used as arbitrarily defined module names. Significant correlation after Bonferroni correction for multiple testing is shown with ^∗^. (B) GO and KEGG enrichment analysis of significantly correlated gene modules with log-transformed adjusted *p* values shown. The red dotted line represents adjusted *p* < 0.05. Enriched pathways converge on immune defects, particularly B cell and other humoral immunity.

To investigate potential mechanisms for autoimmune diabetes and additional immune dysregulatory features, we performed weighted gene co-expression network analysis (WGCNA) on our RNA-seq data.^28^ This identified 2 significantly differentially regulated gene modules in individuals with bi-allelic variants in *RNU4ATAC*/*RNU6ATAC* compared to control subjects (Figure 3A). Enrichment analysis through Gene Ontology^29^^,^^30^ and KEGG^31^ converged on significant enrichment for genes involved in B cell signaling, development, or proliferation and innate immune responses, as well as other immune pathways (Figures 3B and S2).

**Figure 4:**
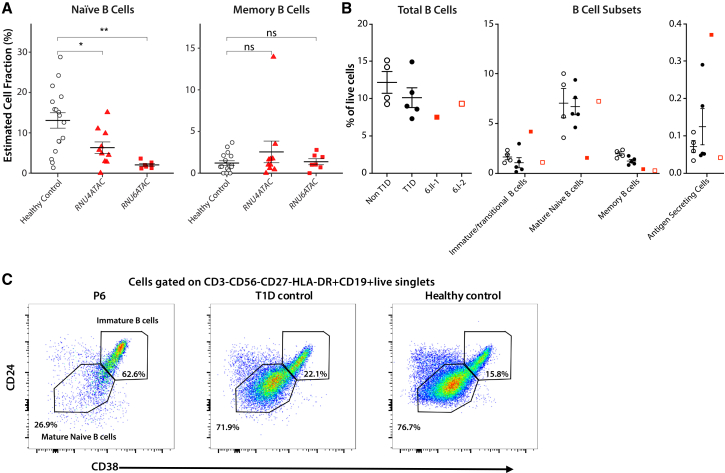
Immune profile analysis of affected individuals using methylation and flow cytometry (A) Deconvolution of whole-blood-derived DNA using the IDOL library identified normal estimates of memory B cells but significantly reduced naive B cells in individuals with variants in both *RNU4ATAC* (*n* = 10) and *RNU6ATAC* (*n* = 7) compared to age-matched control subjects. Bars show mean and standard error. ∗*p* < 0.05, ∗∗*p* < 0.01. (B) Flow cytometry on fresh blood cells of an *RNU4ATAC* affected individual (red triangle) identified reduced naive and increased transitional B cells, as well as elevated antigen-secreting cells (ASCs), compared to healthy control subjects (white circles) and age-matched T1D control subjects (black circles). Bars show mean and standard error. (C) Dot plots of flow cytometry data showing markedly increased immature B cell compartment and reduced mature naive B cells in the individual with bi-allelic variants in *RNU4ATAC* (6.II-2 in Figure 1; Table 2) vs. representative control subjects. Each plotted point for the flow cytometry data in (B) and (C) is from a single blood draw for control subjects, the affected individual, and the carrier parent.

To investigate the impact on B cells, we performed deconvolution analysis of whole-blood genome-wide methylation data from 7 individuals with bi-allelic *RNU6ATAC* variants, 10 individuals with bi-allelic *RNU4ATAC* variants, and 17 age-matched healthy control subjects (Table S4).^32^ This showed significantly reduced estimated naive B cells (Figure 4A), while memory B cells and 10 other immune cell subsets did not show significant differences from control subjects (Figure S3). Deconvolution of RNA-seq data into immune cell subsets also showed reduced naive B cells (Figure S4). To validate these findings, we performed in-depth immune profiling on an individual with bi-allelic *RNU4ATAC* variants (individual 6.II-1, Figure 1A), from whom we were able to obtain a fresh whole-blood sample. This showed a striking B cell developmental defect, with reduced naive and memory B cells and increased transitional B cells and antibody-secreting cells vs. age-matched healthy and T1D control subjects (Figures 4B and 4C). This suggests impaired B cell development and maturation, as has previously been seen in some individuals with RNU4ATAC-opathy.^33^^,^^34^^,^^35^ We also found reduced basophils and increased proliferating CD8^+^ and CD4^+^ T cells (i.e., expressing Ki67), while other cell types were similar to healthy and T1D control subjects (Figure S5). The individual was not known to have an infection when the sample was taken, but we are unable to rule out a nascent infection that could explain these findings.

We report bi-allelic pathogenic variants in *RNU6ATAC* as the cause of a genetic syndrome characterized by monogenic autoimmune diabetes with additional immune dysregulation. We also extend the phenotype associated with bi-allelic variants in *RNU4ATAC* to include early-onset autoimmune diabetes. These represent the first causes of monogenic diabetes resulting from pathogenic variants in non-protein-coding genes.

Our results highlight a key role for the minor spliceosome’s components *RNU6ATAC* and *RNU4ATAC* in immune regulation. We identified 19 individuals with defects in minor spliceosome components; 7 with the *RNU6ATAC*-associated syndrome and 12 with *RNU4ATAC* variants. All had early-onset diabetes, and 12 (63%) had additional immune dysregulatory features. Using a multi-omic approach, combined with islet autoantibody testing, we provide evidence that the diabetes in these individuals is autoimmune and identify a shared B cell developmental defect across both monogenic disorders.

The *RNU6ATAC* and *RNU4ATAC* variants we have identified are predicted to impact U12 intron splicing through several potential mechanisms. Of the 5 different *RNU6ATAC* variants, 2 variants are predicted to impact U12 intron binding directly (n.4T>C andn.6G>A [GenBank: NR_023344.1]; Figure S6), 1 is within the region of *RNU6ATAC* predicted to bind *RNU4ATAC* (n.43G>A [GenBank: NR_023344.1]) and thus may prevent recruitment of *RNU6ATAC* during spliceosome assembly, and 2 have less clear functional consequences but may induce misfolding or instability of the snRNA or its affinity to other components of the minor spliceosome.^13^^,^^17^ None of the *RNU6ATAC* variants were within the region that is predicted to bind *RNU12* (MIM: 620204).^36^ Of the 10 different *RNU4ATAC* variants, 4 were predicted to affect the *RNU6ATAC* binding region (n.13C>T, n.16G>A, n.17G>A, and n.60G>A [GenBank: NR_023343.1]), with the remaining 6 affecting the 5′ stem loop.^13^^,^^17^

Our data support a profound B cell defect resulting from pathogenic variants in *RNU6ATAC* (from affected individuals' RNA-seq and methylation data) or *RNU4ATAC* (from affected individuals' RNA-seq, methylation, and flow cytometry data). This is consistent with humoral immune deficiencies and B cell developmental defects previously reported in some individuals with RNU4ATAC-opathies but without diabetes.^18^^,^^33^ The role of B cells in the pathogenesis of islet autoimmunity is debated; some evidence points to a direct pathogenic role, while other evidence suggests that B cell dysregulation is secondary to islet autoimmunity.^37^ Postmortem pancreases of individuals with the young onset “TIDE1” endotype of T1D show increased insulitic B cells,^38^ and B cell depletion with the monoclonal antibody rituximab has shown some success in delaying T1D progression.^39^ However, an individual with absent B cells due to X-linked agammaglobulinemia (XLA) developed autoimmune diabetes, implying that B cells or autoantibodies are not required for diabetes development.^40^ Further study of individuals with both B cell defects and autoimmune diabetes is warranted to understand the role of B cell regulation, maturation, and development in autoimmune diabetes.

The minor spliceosome is found across many eukaryote genera, though it has been lost in some.^15^ While it is involved in the splicing of a small proportion of genes, many have essential functions, and minor intron splicing may play a role in temporal regulation of protein expression through slower mRNA processing.^41^ This is supported by incomplete intron retention of U12 genes, as seen in the individuals analyzed in this study and previously in RNU4ATAC-opathy.^11^^,^^26^ Further work is needed to untangle the role of genes undergoing minor splicing in the development of beta cell autoimmunity, as it is possible that a subset of these genes has direct pathogenic roles.

We performed methylation analysis on 17 affected individuals (7 *RNU6ATAC* and 10 *RNU4ATAC*) and RNA-seq on 6 affected individuals (3 and 3). The results of these studies supported all individuals having B cell defects, which were further validated in a single individual with bi-allelic *RNU4ATAC* variants using gold-standard immune analyses through flow cytometry. Although we would have liked to study more individuals, the geographic diversity of the cohort and the severity of disease (6 individuals deceased in early life) prevented sample collection from the remaining individuals.

In conclusion, we report bi-allelic pathogenic variants in *RNU6ATAC* as a cause of monogenic autoimmune diabetes with additional immune dysregulation and extend the phenotype of RNU4ATAC-opathies to include autoimmune diabetes. Our work provides insights into the role of these snRNAs in human immune regulation and beta cell autoimmunity and crucial new diagnoses to families.

## Data and code availability

Anonymized RNA-seq data are available through application at the European Genome-phenome Archive web portal (https://ega-archive.org). The accession number for the data reported in this paper is EGA Archive: EGAS50000001565. Access to these data will be granted for appropriate use in research and will be governed by the provisions laid out in the terms contained in the data access agreement. All other non-clinical data analyzed during this study are included in this published article and supplemental information. Additional clinical, methylation array, and genotype data can be used to identify individuals and are therefore available through collaboration with experienced teams working on approved studies examining the mechanisms, causes, diagnosis, and treatment of diabetes and other beta cell disorders. Requests for collaboration will be considered by a steering committee following an application to the Genetic Beta Cell Research Bank (https://www.diabetesgenes.org/current-research/genetic-beta-cell-research-bank/). Contact by email should be directed to Prof. Elisa De Franco (e.de-franco@exeter.ac.uk). All requests for access to data will be responded to within 14 days. The code used in the analysis of the data is publicly available on GitHub (https://github.com/JamesR-S/RNU6ATAC_RNU4ATAC_Monogenic_Diabetes).

## Consortia

The members of the EXE-T1D consortium are Rebecca A. Dobbs, Evangelina Williams, Kathleen M. Gillespie, William A. Hagopian, Amber M. Luckett, Michelle Hudson, Timothy J. McDonald, Noel G. Morgan, Kathryn Murrall, Suraj Ramchand, Sarah J. Richardson, Bart O. Roep, Bradford Dimos, Megan E. Smithmyer, and Cate Speake

The members of the ATAC clinical consortium are Elke Fink-Leinweber, Markus Lundgren, Annelie Carlsson, Ghaisani Fadiana, Frida Soesanti, Elizabeth A. Mann, M. Tracy Bekx, Tabitha Randell, Tugba Kontbay Çetin, Mahsa M. Amoli, Can Thi Bich Ngoc, Dung Chi Vu, Nguyan Hoang Lan, Saif S. Albayati, Nileema Thuse, Kalpana Jog, Chitteranjan Yajnik, Khadija N. Humayun, Patrick Willems, Adel Djermane, and Yasmine Ouarezki

## Acknowledgments

We are grateful to the patients and their families for taking part in our gene discovery study. We thank Sabrina Wright and the Exeter Sequencing Facility (University of Exeter) for technical assistance and Joe Burrage and Dr. Emma Dempster for generating DNA methylation data. We are grateful to Dr. Patrick Willems and the GENDIA (Antwerp, Belgium) team and Dr. Majedah AbdulRasoul and Dr. Maria Al Mahdi (Dasman Institute, Kuwait) for patient referral and for providing clinical details. This study was supported by the National Institute for Health and Care Research Exeter Biomedical Research Centre and the National Institute for Health and Care Research Exeter Clinical Research Facility. The views expressed are those of the authors and not necessarily those of the National Institute for Health and Care Research or the Department of Health and Social Care. M.B.J. is a Diabetes UK and Breakthrough T1D RD Lawrence Fellow (23/0006516). E.D.F. is a Diabetes UK RD Lawrence Fellow (19/005971) and the recipient of a European Foundation for the Study of Diabetes/Novo Nordisk Foundation Future Leaders Award (NNF23SA0087432). K.A.P. has a Wellcome Trust Research Fellowship (219606/Z/19/Z). S.E.F. has a Wellcome Trust Senior Research Fellowship (223187/Z/21/Z). This study was supported by The Leona M. and Harry B. Helmsley Charitable Trust (grants 2016PG-T1D049, 2018PG-T1D049, 2103–05059, and G-2404-06858) and a Wellcome Trust Collaborative Award in Science to E.D.F. and A.T.H. (grant no. 224600/Z/21/Z). For the purpose of open access, the author has applied a CC BY public copyright license to any author-accepted manuscript version arising from this submission.

## Author contributions

M.B.J., J.R.-S., A.T.H., and E.D.F. designed the study, analyzed and interpreted the data, wrote the manuscript, and directed the project. P.A.B., C.D.-V., and T.I.M.T. performed the immunological experiments, interpreted the resulting data, and wrote the manuscript. M.G. and G.B. performed experiments and interpreted the resulting data. M.N.W. and J.R.-S. wrote the scripts to analyze genome sequencing data. M.B.J., S.E.F., K.A.P., E.D.F., A.T.H., and the ATAC clinical consortium recruited patients and interpreted the clinical data. M.J. and E.D.F. interpreted the sequence variants. R.A.O. interpreted the clinical data and recruited patients for flow. The EXE-T1D consortium contributed to the recruitment of individuals for immune studies. All authors contributed to drafting the final manuscript. E.D.F. is the guarantor of the study and data.

## Declaration of interests

The authors declare no competing interests.

## Web resources

European Genome-phenome Archive, https://ega-archive.org
GenBank, https://www.ncbi.nlm.nih.gov/genbank/
OMIM, https://www.omim.org

## Supplemental information

Document S1. Figures S1–S6, Tables S1, S2, and S4–S7, and supplemental material and methods

Table S3. U12-intron-containing genes with intron retention determined by RNA-seq of whole-blood-derived RNA from affected individuals

Document S2. Article plus supplemental information
