# Bi-allelic variants in the non-protein-coding minor spliceosome components *RNU6ATAC* and *RNU4ATAC* cause syndromic monogenic autoimmune diabetes

**DOI:** 10.1016/j.ajhg.2026.07.003

**Published:** 2026-07-14

**Authors:** Matthew B. Johnson, James Russ-Silsby, Paul A. Blair, Molly Govier, Georgia Bonfield, Clara Domingo-Vila, EXE-T1D consortium, ATAC clinical consortium, Matthew N. Wakeling, Richard A. Oram, Sarah E. Flanagan, Timothy I.M. Tree, Kashyap A. Patel, Andrew T. Hattersley, Elisa De Franco

## Main text

(The American Journal of Human Genetics *113*, 877–887; April 2, 2026)

In the original paper, there was a mistake in Figure 1 where the parents in family D were shown as being not available for testing. This was incorrect; the parents in family D were both tested for *RNU6ATAC* variants, and they were each a carrier of one of the variants identified in the offspring. The number of parents tested was erroneously indicated as 19 in the original paper, when it was in fact 21.

It was also found that in Table S2, the incorrect ID was given for some of the individuals analyzed using RNA-seq. In the original version, Table S2 indicates individuals from family C as having been assessed through RNA-seq, when in fact they were the individuals from family D.

Two typos were also corrected in the figure legends: in Figure 2B’s legend, there are 12 genes with intron retention in both cohorts (not 13 as previously stated); in Figure 4C’s legend, the sample ID is 6.II-1 (instead of 6.11-2 as previously stated).

All errors have now been revised in the original article and supplemental material. The authors regret these errors.

**Figure 1:**
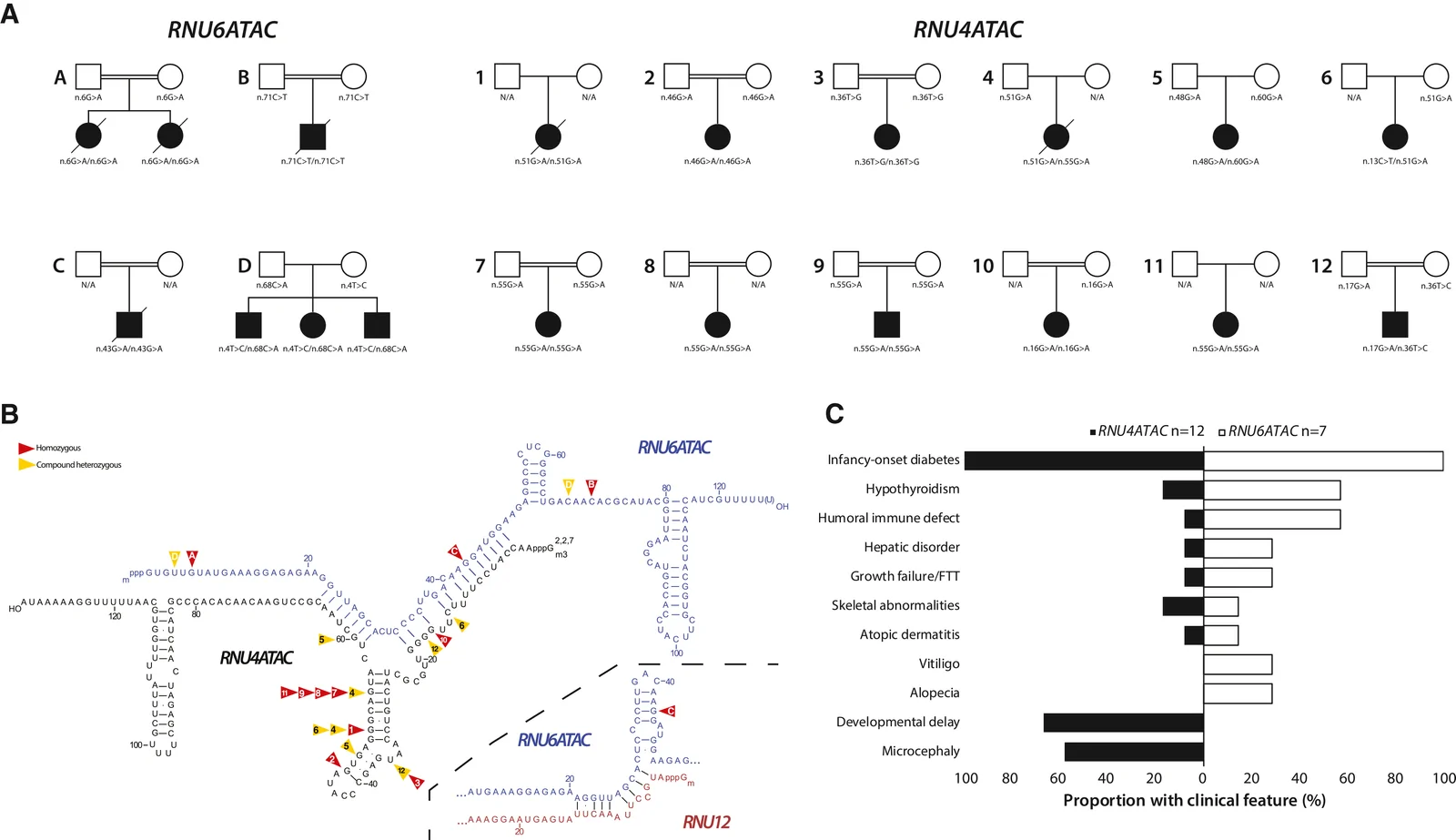
Genetic and clinical information for the RNU6ATAC and RNU4ATAC cohorts (corrected)

**Figure 1:**
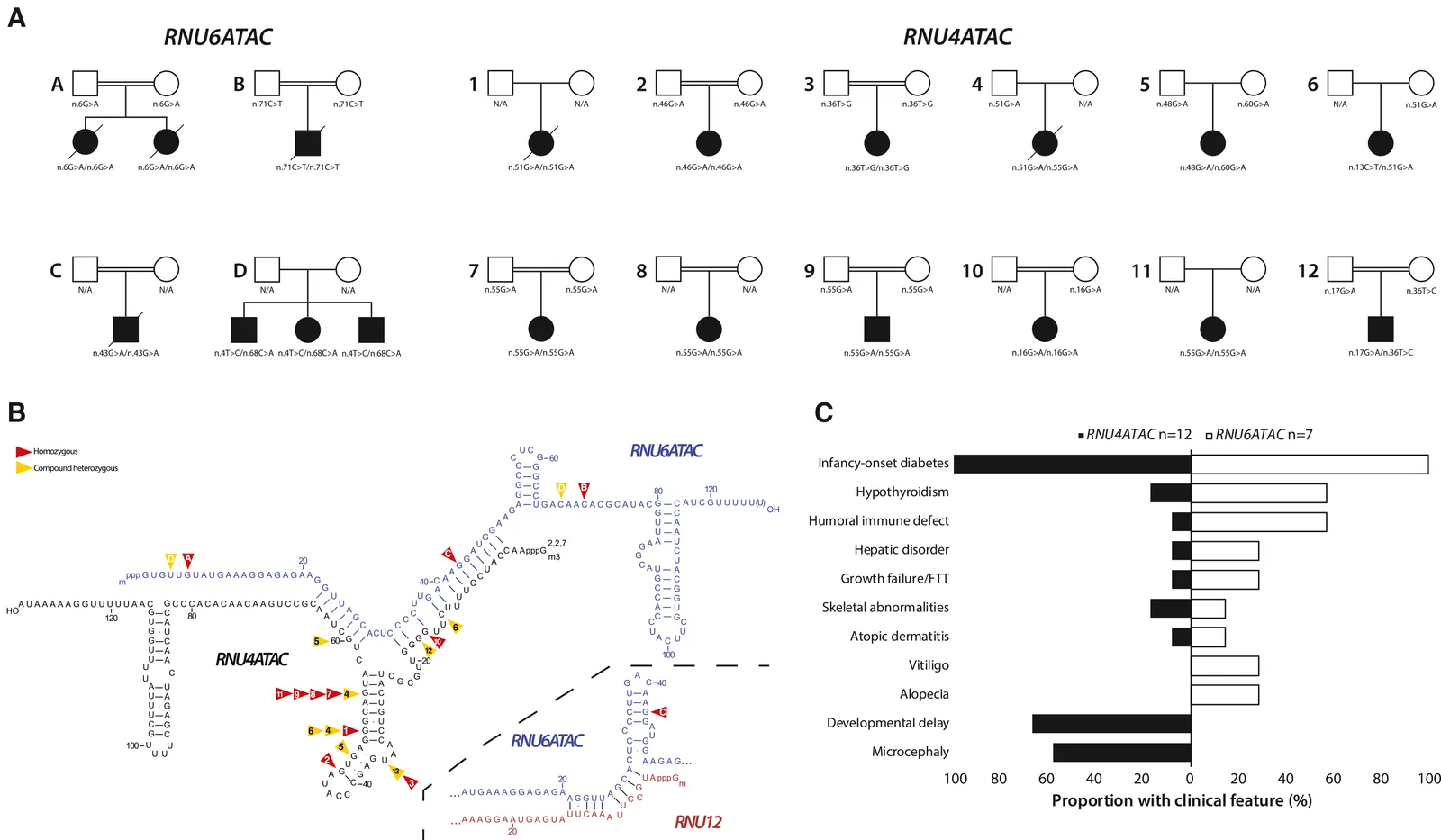
Genetic and clinical information for the RNU6ATAC and RNU4ATAC cohorts (original)

