# Supplementary material for: Bi-allelic variants in the non-protein-coding minor spliceosome components RNU6ATAC and RNU4ATAC cause syndromic monogenic autoimmune diabetes
Source: Am J Hum Genet. 2026 Mar 20;113(4):877–87. doi: 10.1016/j.ajhg.2026.02.017 (PMC13087456; doi:10.1016/j.ajhg.2026.02.017)
Supplement: Document S1. Figures S1–S6, Tables S1, S2, and S4–S7, and supplemental material and methods [file mmc1.pdf]

The American Journal of Human Genetics, Volume *113*

Supplemental information

Bi-allelic variants in the non-protein-coding minor spliceosome components *RNU6ATAC* and *RNU4ATAC* cause syndromic monogenic autoimmune diabetes

Matthew B. Johnson, James Russ-Silsby, Paul A. Blair, Molly Govier, Georgia Bonfield, Clara Domingo-Vila, EXE-T1D consortium, ATAC clinical consortium, Matthew N. Wakeling, Richard A. Oram, Sarah E. Flanagan, Timothy I.M. Tree, Kashyap A. Patel, Andrew T. Hattersley, and Elisa De Franco

1. EXE-T1D CONSORTIUM

R A Dobbs[1], E Williams[2], K M Gillespie[3], W A Hagopian[4,5], A M Luckett[1], M Hudson[1], T J McDonald[1], N G Morgan[1], K Murrall[1], S Ramchand[1], S J Richardson[1], B O Roep[6], B Dimos[7], M E Smithmyer[7], C Speake[7]

1. Department of Clinical & Biomedical Sciences, University of Exeter Medical School, Exeter, UK
2. Department of Immunobiology, School of Immunology & Microbial Sciences (SIMS), King's College, London, UK
3. Translational Health Sciences, Bristol Medical School, University of Bristol, Southmead Hospital, Bristol, UK
4. Department of Paediatrics, Indiana University School of Medicine, Indianapolis, IN, USA.
5. Department of Medicine, University of Washington, Seattle, WA, USA.
6. Department of Internal Medicine, Leids Universitair Medisch Centrum, Leiden, Netherlands
7. Center for Interventional Immunology, Benaroya Research Institute, Seattle, WA

2. ATAC CLINICAL CONSORTIUM

E Fink-Leinweber[1], M Lundgren[2,3], A Carlsson[4], G Fadiana[5], F Soesanti[5], E A Mann[6], M T Bekx[7], T Randell[8], T Kontbay Çetin[9], M M Amoli[10], Can Thi Bich Ngoc[11], D C Vu[11], N H Lan[11], S S Albayati [12], N Thuse[13], K Jog[13], C Yajnik[13], K N Humayun[14], P Willems[15], A Djermane[16, 17], Y Ouarezki[16, 17]

1 - Märkische Kliniken GmbH Klinikum Lüdenscheid, Lüdenscheid, Germany
2 - Department of clinical sciences Malmö, Lund university, Sweden
3 - Skåne university Hospital, Kristianstad, Sweden
4 - Department of Pediatrics, Skånes University Hospital, Lund University, Sweden
5 - Child Health Department, Faculty of Medicine, Universitas Indonesia, Cipto Mangunkusumo General Hospital, Jakarta, Indonesia
6- Division of Endocrinology and Diabetes, Department of Pediatrics, University Hospital, Madison, US
7 - American Family Children’s Hospital, University of Wisconsin-Madison, Madison Wisconsin
8 - Nottingham University Hospitals NHS Trust, Nottingham, UK
9 - Sanliurfa research and training hospital, Sanliurfa, Turkey
10 - Metabolic Disorders Research Centre, Endocrinology and Metabolism Molecular-Cellular Sciences Institute, Tehran University of Medical Sciences, Tehran, Iran.
11 - Center for Endocrinology, Metabolism, Genetics/Genomics and Molecular Therapy, National Children's Hospital, Hanoi, Vietnam
12 - Child central teaching hospital, Baghdad, Iraq
13 - Diabetes Unit, KEM Hospital and Research Centre, Pune, India
14 - Aga Khan University, Karachi, Pakistan
15 - Gendia, Antwerp, Belgium
16 - EPH Hassan Badi, El-Harrach, Algiers, Algeria
17 - Université de Sciences de la Santé, Faculté de médecine d'Alger, Algeria

2. Supplementary Figures

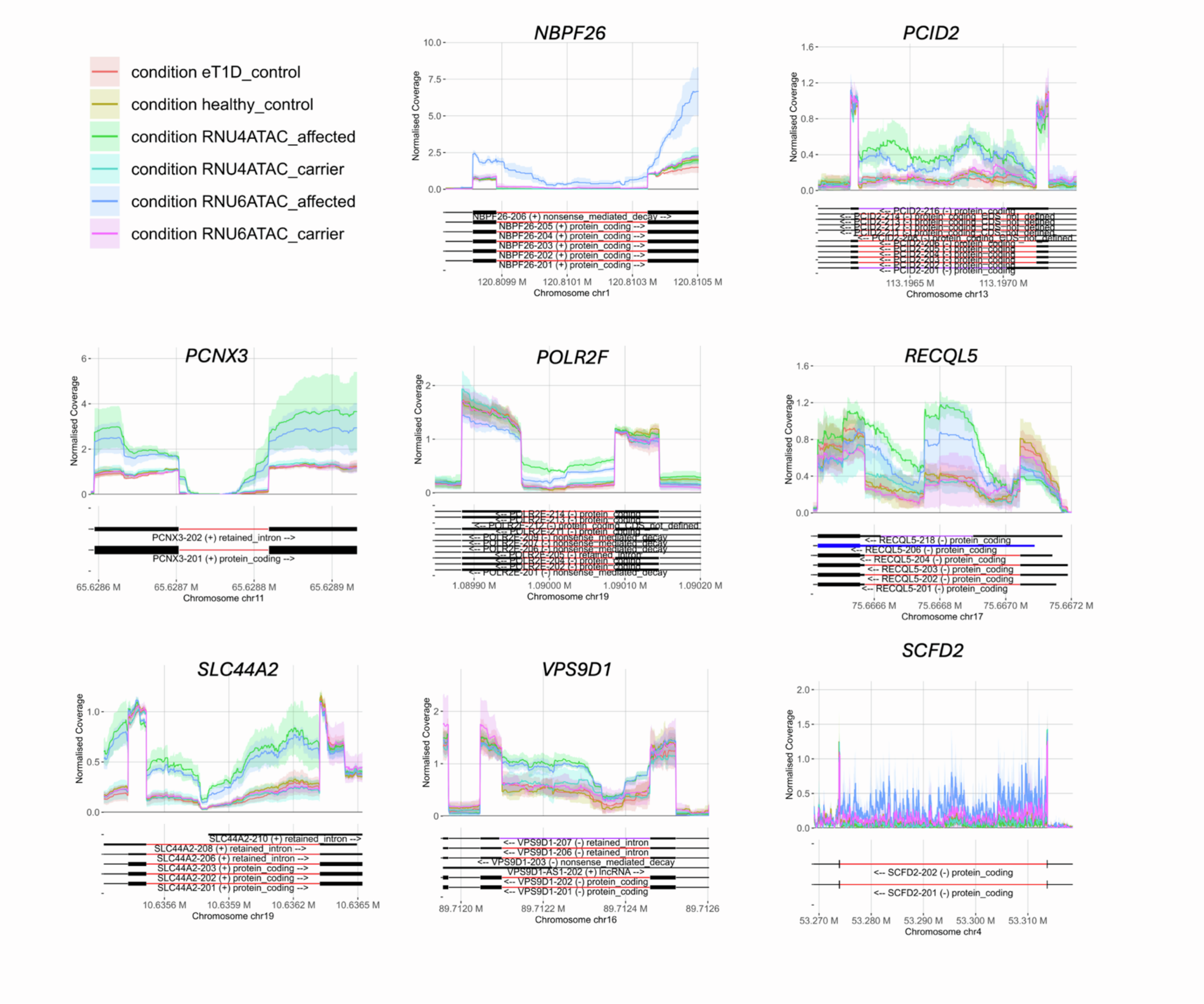

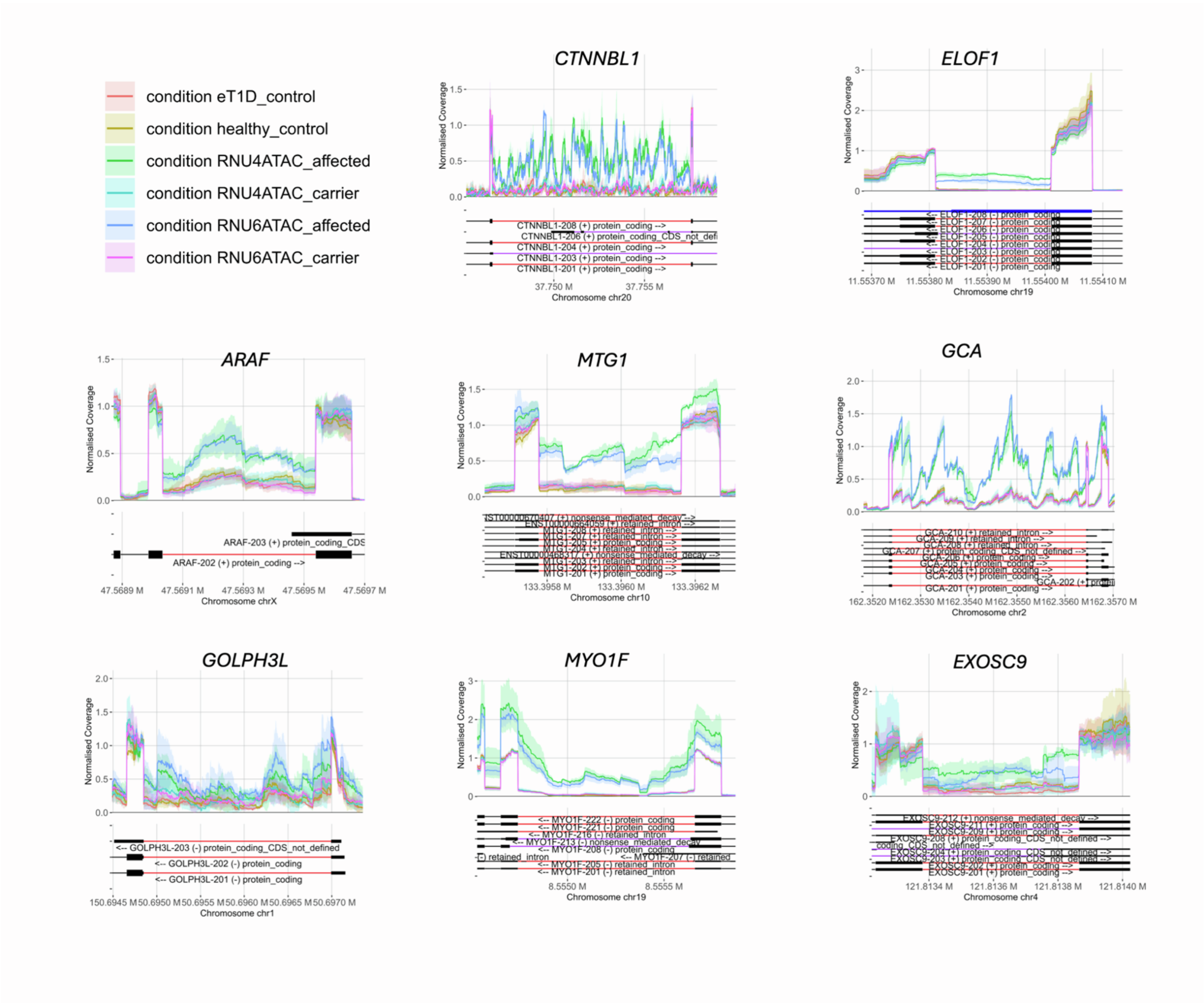

Figure S1: Coverage plots showing putative novel U12 genes. Genes with significant intron retention in whole blood RNA from individuals with biallelic pathogenic variants in *RNU4ATAC*, *RNU6ATAC* or both (table S3) but not present in the IAOD database are shown (https://introndb.lerner.ccf.org/)[3].

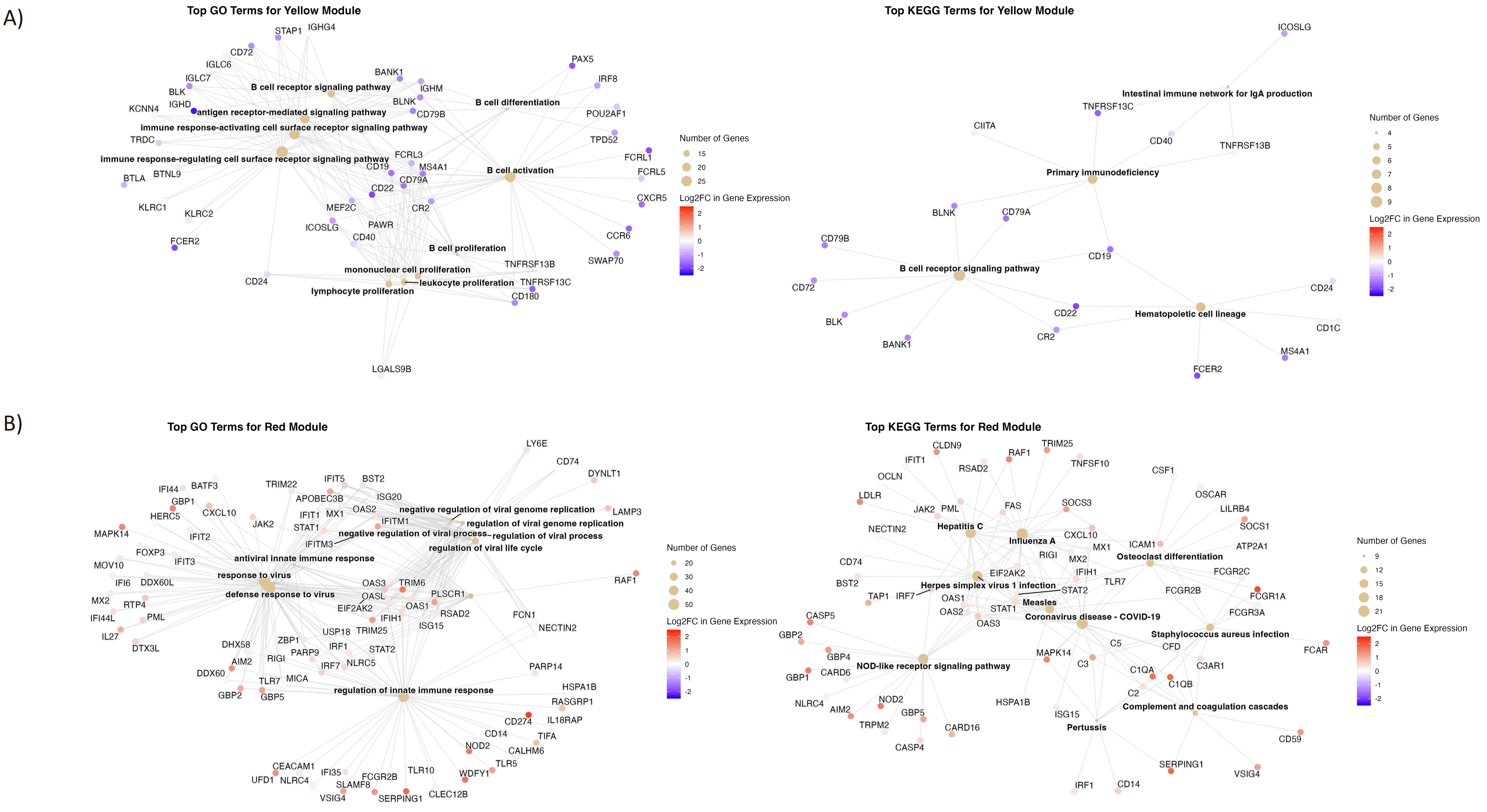

Figure S2: Enrichment analysis of WGCNA gene modules. For the two significant modules, GO and KEGG enrichment was used to identify pathways connected to the gene lists.

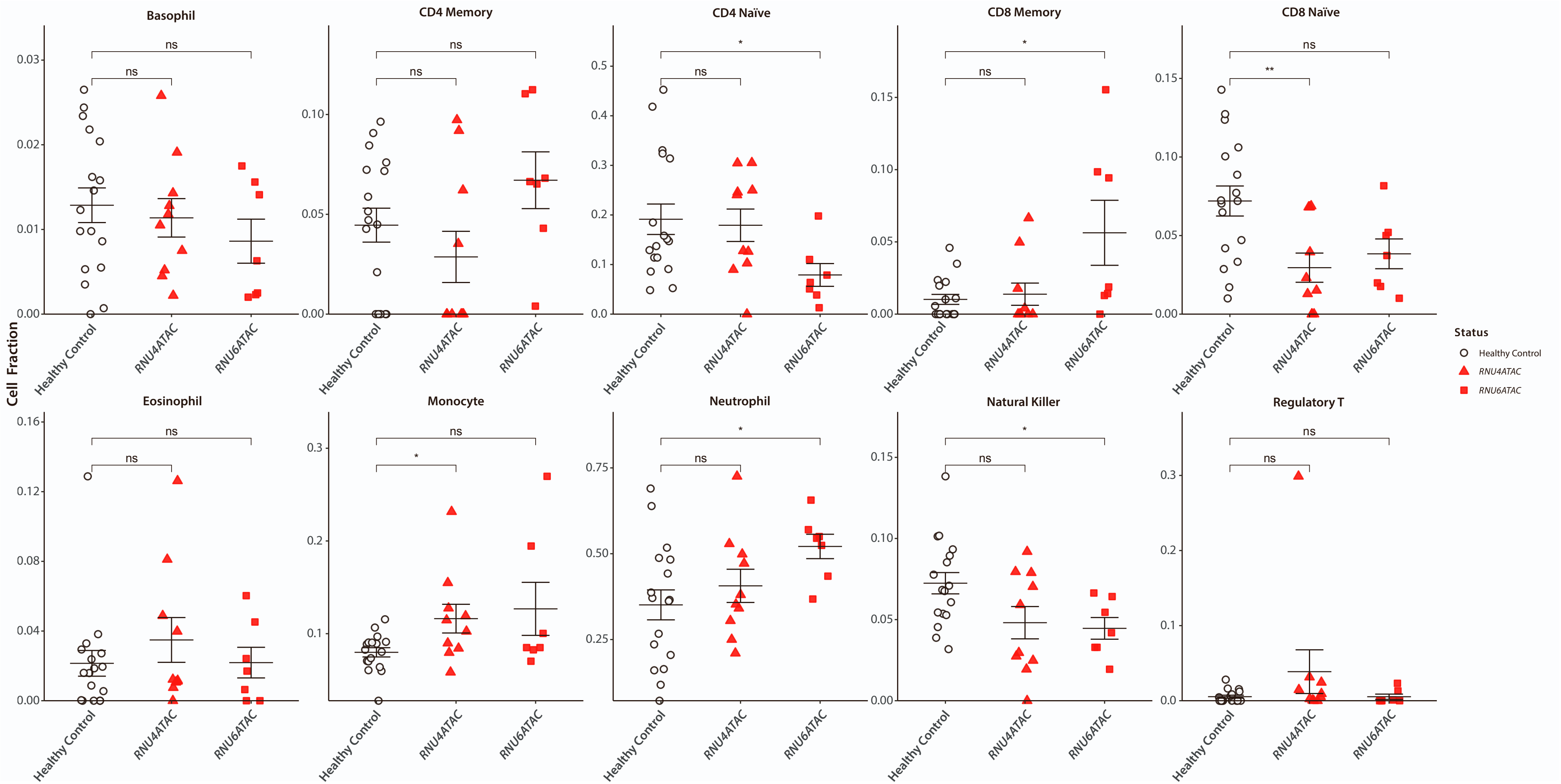

Figure S3: Estimates of immune cell proportions from deconvolution of EPIC array methylation analysis of whole blood-derived DNA.

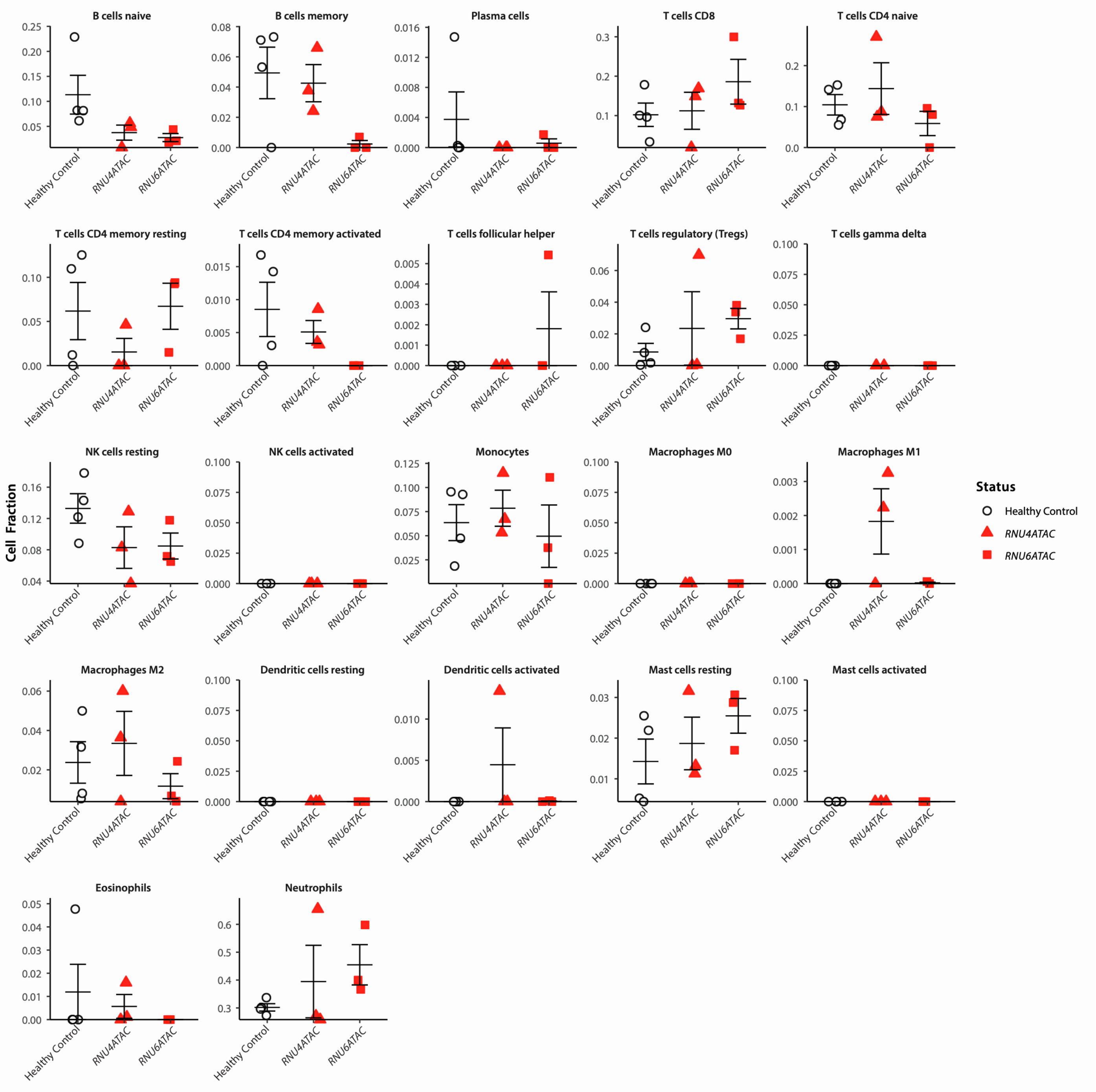

Figure S4: Estimates of immune cell proportions in individuals with biallelic variants in *RNU4ATAC* and *RNU6AAC* and healthy controls derived from deconvolution of whole blood RNA-Sequencing data deconvolution performed using CibersortX[4].

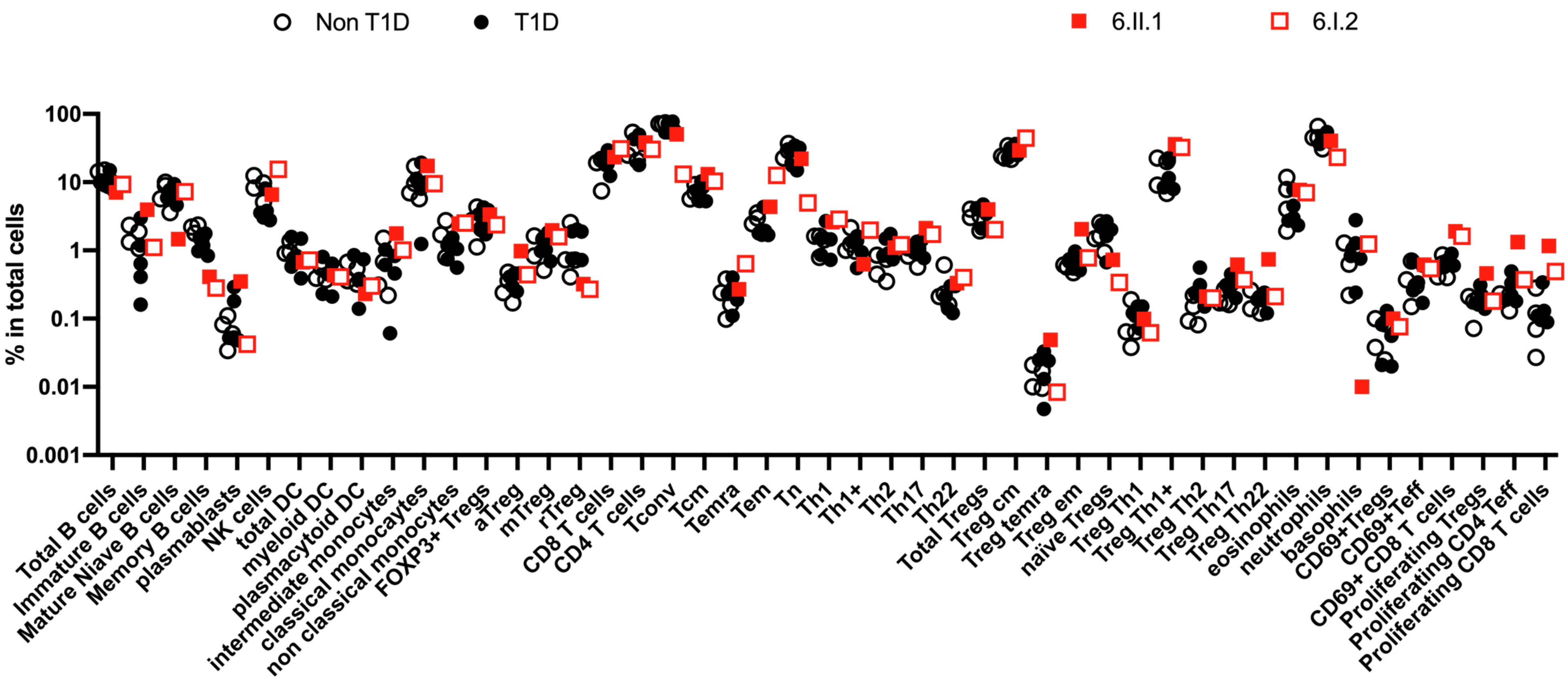

Figure S5: **Immune phenotyping of individual with biallelic *RNU4ATAC* variants, their unaffected heterozygous carrier mother, and age matched controls**. 6.I.2 – mother of 6.II.1.

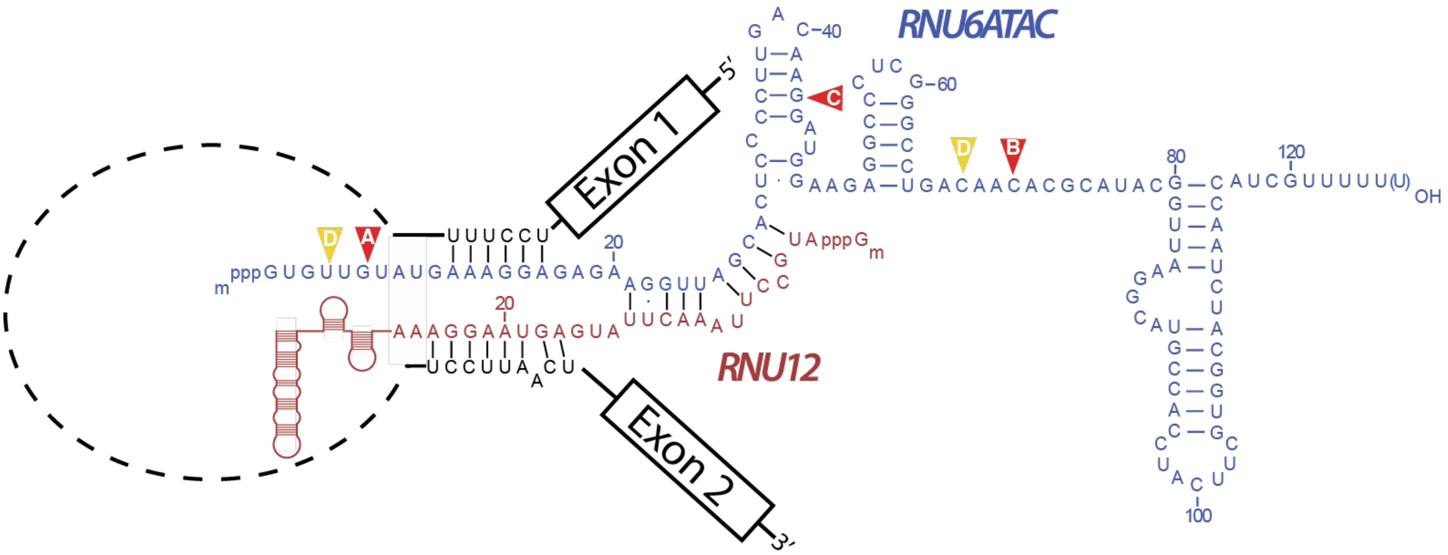

Figure S6 Positions of *RNU6ATAC* variants within the RNU6ATAC-RNU12-U12 intron complex. Dashed line shows U12 intron. Red triangles show homozygous variants, yellow triangles show compound heterozygous variants.

3. SUPPLEMENTARY TABLES

Protein	Subcomplex	Gene	Chr	Start	End	OMIM Inheritance	OMIM disease(s)
PRP8	U5 snRNP	*PRPF8*	17	1553923	1588176	Autosomal Dominant	RETINITIS PIGMENTOSA 13
SNU114	U5 snRNP	*EFTUD2*	17	42927311	42977030	Autosomal Dominant	MANDIBULOFACIAL DYSOSTOSIS, GUION-ALMEIDA TYPE
BRR2	U5 snRNP	*SNRNP200*	2	96940074	96971297	Autosomal Dominant	RETINITIS PIGMENTOSA 33
U5-40K	U5 snRNP	*SNRNP40*	1	31732417	31769662	-	None
PRP6	U5 snRNP	*PRPF6*	20	62612488	62664453	Autosomal Dominant	RETINITIS PIGMENTOSA 60
DIM2	U5 snRNP	*TXNL4B*	16	72078188	72128330	-	None
PRP28	U5 snRNP	*DDX23*	12	49223547	49246625	-	None
SmD3	U5 snRNP,U11 snRNP, U12 snRNP & U4atac/U6atac di-snRNP	*SNRPD3*	22	24951471	25005947	-	None
SmB	U5 snRNP,U11 snRNP, U12 snRNP & U4atac/U6atac di-snRNP	*SNRPB*	20	2442280	2451499	Autosomal Dominant	CEREBROCOSTOMANDIBULAR SYNDROME
SmD1	U5 snRNP,U11 snRNP, U12 snRNP & U4atac/U6atac di-snRNP	*SNRPD1*	18	19192228	19210417	-	None
SmD2	U5 snRNP,U11 snRNP, U12 snRNP & U4atac/U6atac di-snRNP	*SNRPD2*	19	46190712	46195827	-	None
SmF	U5 snRNP,U11 snRNP, U12 snRNP & U4atac/U6atac di-snRNP	*SNRPF*	12	96252706	96297606	-	None
SmE	U5 snRNP,U11 snRNP, U12 snRNP & U4atac/U6atac di-snRNP	*SNRPE*	1	203830731	203839678	Autosomal Dominant	Hypotrichosis 11 and Microcephaly(pending confirmation)
SmG	U5 snRNP,U11 snRNP, U12 snRNP & U4atac/U6atac di-snRNP	*SNRPG*	2	70508494	70520903	-	None
SCNM1	U12 snRNP	*SCNM1*	1	151129140	151142773	Autosomal Recessive	OROFACIODIGITAL SYNDROME XIX
SF3b155	U12 snRNP	*SF3B1*	2	198254508	198299815	Somatic	myelodysplastic syndrome(somatic mutations)
SF3b145	U12 snRNP	*SF3B2*	11	65818200	65836779	Autosomal Dominant	CRANIOFACIAL MICROSOMIA 1
SF3b130	U12 snRNP	*SF3B3*	16	70557691	70608820	-	None
SF3b49	U12 snRNP	*SF3B4*	1	149895209	149900236	Autosomal Dominant	ACROFACIAL DYSOSTOSIS 1, -GER TYPE
SF3b14a	U12 snRNP	*SF3B6*	2	24290454	24299313	-	None
SF3b14b	U12 snRNP	*PHF5A*	22	41855721	41864729	-	None
SF3b10	U12 snRNP	*SF3B5*	6	144416018	144416754	-	None
U11/U12-31K	U12 snRNP	*ZCRB1*	12	42705880	42719920	-	None
U11/U12-65K	U12 snRNP	*RNPC3*	1	104068313	104097861	Autosomal Recessive	PITUITARY HORMONE DEFICIENCY, COMBINED, 7
ZRSR2	U12 snRNP	*ZRSR2*	X	15808595	15841383	-	None
CDC5L	nineteen complex (NTC)	*CDC5L*	6	44355262	44418163	-	None
SYF3	nineteen complex (NTC)	*CRNKL1*	20	20015012	20036690	-	None
CWC15	NTC-related (NTR)	*CWC15*	11	94695787	94706776	-	No Entry
SKIP	NTC-related (NTR)	*SNW1*	14	78183942	78227550	-	None
PLRG1	NTC-related (NTR)	*PLRG1*	4	155456158	155471587	-	None
SNIP1	retention and splicing (RES) complex	*SNIP1*	1	38000050	38019903	Autosomal Recessive	NEURODEVELOPMENTAL DISORDER WITH HYPOTONIA, CRANIOFACIAL ABNORMALITIES, AND SEIZURES
RBMX2	retention and splicing (RES) complex	*RBMX2*	X	129535943	129547317	-	No entry
BUD13	retention and splicing (RES) complex	*BUD13*	11	116618886	116643704	Autosomal Recessive	Achalasia-progeroid syndrome
PPIL2	prolyl peptidyl isomerase (PPIase)–like proteins	*PPIL2*	22	22006559	22054304	-	None
CWC27	prolyl peptidyl isomerase (PPIase)–like proteins	*CWC27*	5	64064757	64314590	Autosomal Recessive	RETINITIS PIGMENTOSA WITH OR WITHOUT SKELETAL ANOMALIES
PRP2	Splicing Factors	*DHX16*	6	30620896	30640814	Autosomal Dominant	NEUROMUSCULAR OCULOAUDITORY SYNDROME
GPKOW	Splicing Factors	*GPKOW*	X	48970334	48980151	X-Linked Recessive	X-linked microhydranencephaly (unconfuirmed)
RNF113A	Splicing Factors	*RNF113A*	X	119004497	119005791	X-Linked Recessive	TRICHOTHIODYSTROPHY 5, NONPHOTOSENSITIVE
SRm300	Splicing Factors	*SRRM2*	16	2802330	2822539	Autosomal Dominant	INTELLECTUAL DEVELOPMENTAL DISORDER, AUTOSOMAL DOMINANT 72

CWC22	Splicing Factors	*CWC22*	2	180809603	180871840	-	None
SRm160	Splicing Factors	*SRRM1*	1	24958207	24999758	-	None
CRIPT	Splicing Factors	*CRIPT*	2	46843555	46852881	Autosomal Recessive	ROTHMUND-THOMSON SYNDROME, TYPE 3
RBM48	Splicing Factors	*RBM48*	7	92158087	92167319	-	None
ARMC7	Splicing Factors	*ARMC7*	17	73106047	73126360	-	No Entry
PRP3	U4atac/U6atac di-snRNP	*PRPF3*	1	150293925	150325671	Autosomal Dominant	RETINITIS PIGMENTOSA 18
PRP4	U4atac/U6atac di-snRNP	*PRPF4*	9	116037623	116055185	Autosomal Dominant	RETINITIS PIGMENTOSA 70
PRP31	U4atac/U6atac di-snRNP	*PRPF31*	19	54618837	54635140	Autosomal Dominant	RETINITIS PIGMENTOSA 11
SNU13	U4atac/U6atac di-snRNP	*SNU13*	22	42069934	42086508	-	None
CENATAC	U4atac/U6atac di-snRNP	*CE-TAC*	11	118868852	118886501	Autosomal Recessive	MOSAIC VARIEGATED ANEUPLOIDY SYNDROME 4
LSM2	U4atac/U6atac di-snRNP	*LSM2*	6	31765173	31774761	-	None
LSM3	U4atac/U6atac di-snRNP	*LSM3*	3	14219858	14242619	-	None
LSM4	U4atac/U6atac di-snRNP	*LSM4*	19	18417040	18434084	-	None
LSM5	U4atac/U6atac di-snRNP	*LSM5*	7	32524951	32534895	-	None
LSM6	U4atac/U6atac di-snRNP	*LSM6*	4	147096837	147121152	-	None
LSM7	U4atac/U6atac di-snRNP	*LSM7*	19	2321516	2328619	-	None
LSM8	U4atac/U6atac di-snRNP	*LSM8*	7	117824086	117832878	-	None
U11/U12-20K	U11 snRNP	*ZMAT5*	22	30126945	30163000	-	None
U11/U12-25K	U11 snRNP	*SNRNP25*	16	103010	107669	-	No Entry
U11/U12-35K	U11 snRNP	*SNRNP35*	12	123942188	123957701	-	None
U11/U12-48K	U11 snRNP	*SNRNP48*	6	7590432	7612200	-	No Entry
U11/U12-59K	U11 snRNP	*PDCD7*	15	65409717	65426174	-	None
SADI	Tri-snRNP specific	*USP39*	2	85829979	85876403	-	None
SNU66	Tri-snRNP specific	*SART1*	11	65729160	65747299	-	None
PRP4 ki-se	Pre-B specific	*PRP4K*	6	4021534	4065217	-	None

Table S1: minor spliceosome genes [1,2].

Sample	Condition	Sex	Age collected (years)	Age diagnosed with diabetes
T1D1	eT1D control	M	10	58
T1D2	eT1D control	M	5	20
T1D3	eT1D control	F	6	44
T1D4	eT1D control	F	8	56
HEAL1	Healthy control	F	4	-
HEAL2	Healthy control	M	5	-
HEAL3	Healthy control	M	3	-
HEAL4	Healthy control	F	5	-
8	*RNU4ATAC* affected	F	9	26
8 mother	*RNU4ATAC* carrier	M	44	-
8 sibling	*RNU4ATAC* carrier	F	18	
Ci	*RNU6ATAC* affected	M	3	5
C mother	*RNU6ATAC* carrier	F	35	-
C father	*RNU6ATAC* carrier	M	42	-
Ciii	*RNU6ATAC* affected	F	15	260
Cii	*RNU6ATAC* affected	M	8	104
A mother	*RNU6ATAC* carrier	F	27	-
A father	*RNU6ATAC* carrier	M	30	-
11	*RNU4ATAC* affected	F	4	20
11 mother	*RNU4ATAC* carrier	F	29	-
11 father	*RNU4ATAC* carrier	M	40	-
6	*RNU4ATAC* affected	F	11	10
6 mother	*RNU4ATAC* carrier	F	46	-

Table S2: Details of samples used for RNA-seq experiments. eT1D – early-onset type 1 diabetes. M – Male. F – Female.

ID	Status	Age collected (years)
1	*RNU4ATAC*	0.4
2	*RNU4ATAC*	1.6
3	*RNU4ATAC*	0.8
4	*RNU4ATAC*	0.2
5	*RNU4ATAC*	0.8
6	*RNU4ATAC*	0.2
7	*RNU4ATAC*	1.1
8	*RNU4ATAC*	9.7
9	*RNU4ATAC*	0.9
12	*RNU4ATAC*	0.4
Aii	*RNU6ATAC*	0.2
Aii	*RNU6ATAC*	24.3
B	*RNU6ATAC*	0.3
Ci	*RNU6ATAC*	0.1
Cii	*RNU6ATAC*	11.3
Ciii	*RNU6ATAC*	8.7
D	*RNU6ATAC*	5.0
H1	Healthy Control	0.2
H2	Healthy Control	0.3
H3	Healthy Control	0.3
H4	Healthy Control	0.3
H5	Healthy Control	0.7
H6	Healthy Control	0.7
H7	Healthy Control	0.8
H8	Healthy Control	1.3
H9	Healthy Control	1.7
H10	Healthy Control	2.4
H11	Healthy Control	3.3
H12	Healthy Control	4.6
H13	Healthy Control	6.5
H14	Healthy Control	10.2
H15	Healthy Control	12.8
H16	Healthy Control	19.2
H17	Healthy Control	25.7

Table S4: Samples and controls for methylation analysis

Marker (surface)	Fluorochrome	Clone	Company	Panel
Brilliant stain buffer	N/A	N/A	BD Biosciences	All
CD3	APCCy7	OKT3	BioLegend	T cell
CD4	BUV395	SK3	BD Biosciences	T cell
CD25	BB515	2A3	BD Biosciences	T cell
CD25	BB515	M-A251	BD Biosciences	T cell
CD127	APC	A019D5	BioLegend	T cell
CD45RA	BV785	HI100	BioLegend	T cell
CD197/CCR7	BV421	G043H7	BioLegend	T cell
CD183/CXCR3	BV510	G025H7	BioLegend	T cell
CD194/CCR4	BV605	L291H4	BioLegend	T cell
CD196/CCR6	BUV737	11A9	BD Biosciences	T cell
CD95	PE	DX2	BioLegend	T cell
CCR10	PerCP-Cy5.5	1B5	BD Biosciences	T cell
CD185/CXCR5	PE-Cy7	J252D4	BioLegend	T cell
CD278/ICOS	BV711	DX29	BD Biosciences	T cell
CD279/PD-1	PE-Dazzle 594	EH12.2H7	BioLegend	T cell
CD3	APC-Cy7	OKT3	BioLegend	B/DC/Monocyte
CD19	PE	HIB19	BioLegend	B/DC/Monocyte
CD14	PerCP-Cy5.5	HCD14	BioLegend	B/DC/Monocyte
CD16	PE-Cy7	3G8	BioLegend	B/DC/Monocyte
CD56	APC	HCD56	BioLegend	B/DC/Monocyte
HLA-DR	FITC	LN3	BioLegend	B/DC/Monocyte
CD123	BUV395	7G3	BD Biosciences	B/DC/Monocyte
CD11c	BV421	Bu15	BioLegend	B/DC/Monocyte
IgD	BV605	IA6-2	BioLegend	B/DC/Monocyte
CD27	BUV737	L128	BD Biosciences	B/DC/Monocyte
CD38	BV785	HIT2	BioLegend	B/DC/Monocyte
CD24	BV510	ML5	BioLegend	B/DC/Monocyte
CD45	PerCP	HI30	BioLegend	Granulocyte
CD3	APC-Cy7	OKT3	BioLegend	Granulocyte
CD19	APC-Cy7	HIB19	BioLegend	Granulocyte
CD56	APC-Cy7	HCD56	BioLegend	Granulocyte
CD14	AF488	HCD14	BioLegend	Granulocyte
CD15	BV605	W6D3	BioLegend	Granulocyte
CD64	BV421	10.1	BioLegend	Granulocyte
CD63	APC	HC56	BioLegend	Granulocyte
CD123	BUV395	7G3	BD Biosciences	Granulocyte
CD294	PE	BM16	Biolegend	Granulocyte
CD203c	BV510	NP4D6	BioLegend	Granulocyte
CD69	PE-Cy7	FN50	BioLegend	Granulocyte
CD45	PerCP	HI30	BioLegend	Lineage
CD3	APC-Cy7	OKT3	BioLegend	Lineage
CD4	BUV395	SK3	BD Biosciences	Lineage

CD8	BUV737	SK1	BD Biosciences	Lineage
CD19	PE	HIB19	BioLegend	Lineage
CD14	AF488	HCD14	BioLegend	Lineage
CD16	PE-Cy7	3G8	BioLegend	Lineage
CD15	BV605	W6D3	BioLegend	Lineage
CD56	APC	HCD56	BioLegend	Lineage
CD45RA	BV785	HI100	BioLegend	Treg
CD15s	BV510	CSLEX1	BD Biosciences	Treg
CD3	BV605	OKT3	BioLegend	Treg
CD4	BUV395	SK3	BD Biosciences	Treg
CD8	BUV737	SK1	BD Biosciences	Treg
CD25	PE	M-A251	BD Biosciences	Treg
FOXP3	AF647	259D	Beckman Coulter	Treg
Helios	Pacific Blue	22F6	BioLegend	Treg
Ki67	FITC	B56	BD Biosciences	Treg
CD69	PE-Cy7	FN50	BioLegend	Treg

Table S5: Monoclonal antibodies used in the flow cytometry panels

Total B cells	CD45+CD15-CD3-CD19+
IgD+ B cells	CD3-CD19+IgD+
Immature B cells	CD3-CD19+CD27-CD24hiCD38hi
Mature Naïve B cells	CD3-CD19+CD27-CD24+CD38h+
Memory B cells	CD3-CD19+CD27+CD24+CD38lo/-
Plasmablasts/Antibody secreting cells (ASCs)	CD3-CD19+CD27+CD24-CD38+/hi
Natural Killer (NK) cells	CD3CD19-CD14-CD56+
Total Dendritic Cells (DC)	CD3-CD19-CD14-CD16-CD56lo/-HLA-DR+
Myeloid DC	CD3-CD19-CD14-CD16-CD56lo/-HLA-DR+CD11c+CD123-
Plasmacytoid DC	CD3-CD19-CD14-CD16-CD56lo/-HLA-DR+CD11-CD123+
Intermediate monocytes	CD3-CD19-CD56-/loHLA-DR+CD14+CD16+
Classical monocytes	CD3-CD19-CD56-/loHLA-DR+CD14+CD16-
Non classical monocytes	CD3-CD19-CD56-/loHLA-DR+CD14lo/-CD16+
FOXP3+ Tregs	CD3+CD4+CD25+FOXP3+
Activated regulatory T cell (aTreg)	CD3+CD4+CD25+FOXP3hiCD45RAlo/-
Memory Treg (mTreg)	CD3+CD4+CD25+FOXP3+CD45RAlo/-
Resting Treg (rTreg)	CD3+CD4+CD25+FOXP3+ CD45RA+
CD8 T cells	CD45+CD15-CD19-CD56-CD3+CD4-CD8+
CD4 T cells	CD45+CD15-CD19-CD56-CD3+CD+CD8-
Conventional T cell (Tconv)	CD45+CD15-CD19-CD56-CD3+
Central memory T cell (Tcm)	CD3+CD4+CD127+/hiCD25lo/-CCR7+CD45RA-
Terminally differentiated effector memory T cell (Temra)	CD3+CD4+CD127+/hiCD25lo/-CCR7-CD45RA+
T effector memory (Tem)	CD3+CD4+CD127+/hiCD25lo/-CCR7-CD45RA-
Naïve T cell (Tn)	CD3+CD4+CD127+/hiCD25lo/-CD95-CCR7+CD45RAhi
T helper cell type 1 (Th1)	CD3+CD4+CD127+/hiCD25lo/-CCR7varCD45RAvarCXCR5-CCR4-CXCR3+CCR10-CCR6-
T helper cell type 1+ (Th1+)	CD3+CD4+CD127+/hiCD25lo/-CCR7varCD45RAvarCXCR5-CCR4-CXCR3+CCR10-CCR6+
T helper cell type 2 (Th2)	CD3+CD4+CD127+/hiCD25lo/-CCR7varCD45RAvarCXCR5-CCR4+CXCR3-CCR10-CCR6-
T helper cell type 17 (Th17)	CD3+CD4+CD127+/hiCD25lo/-CCR7varCD45RAvarCXCR5-CCR4+CXCR3-CCR10-CCR6+
T helper cell type 22 (Th22)	CD3+CD4+CD127+/hiCD25lo/-CCR7varCD45RAvarCXCR5-CCR4+CXCR3-CCR10+CCR6+
Total Tregs	CD3+CD4+CD127-CD25+
Treg central memory (cm)	CD3+CD4+CD127-CD25+CCR7+CD45RA-
Treg Temra	CD3+CD4+CD127-CD25+CCR7-CD45RA+
Treg effector memory (em)	CD3+CD4+CD127-CD25+CCR7-CD45R-
Naïve Tregs	CD3+CD4+CD127-CD25+CD95-CCR7+CD45RAhi
Treg Th1	CD3+CD4+CD127-CD25l+CCR7varCD45RAvarCXCR5-CCR4-CXCR3+CCR10-CCR6-
Treg Th1+	CD3+CD4+CD127-CD25l+CCR7varCD45RAvarCXCR5-CCR4-CXCR3+CCR10-CCR6+
Treg Th2	CD3+CD4+CD127-CD25l+CCR7varCD45RAvarCXCR5-CCR4+CXCR3-CCR10-CCR6-
Treg Th17	CD3+CD4+CD127-CD25l+CCR7varCD45RAvarCXCR5-CCR4+CXCR3-CCR10-CCR6+

Treg Th22	CD3+CD4+CD127-CD25l+CCR7varCD45RAvarCXCR5-CCR4+CXCR3-CCR10+CCR6+
Eosinophils	CD45+CD3-CD14-CD19-CD56-CD15+CD294+CD203c+
Neutrophils	CD45+CD3-CD14-CD19-CD56-CD15+CD294-CD203clo/-
Basophils	CD45+CD3-CD14-CD19-CD56-CD15-CD123+CD294+
CD69+Tregs	CD3+CD4+CD25+FOXP3+CD69+
CD69+ effector T cells (Teff)	CD3+CD4+CD25-FOXP3-CD69+
CD69+ CD8 T cells	CD3+CD-CD8+CD69+
Proliferating Tregs	CD3+CD4+CD25+FOXP3+Ki67+
Proliferating CD4 Teff	CD3+CD4+CD25-FOXP3- Ki67+
Proliferating CD8 T cells	CD3+CD4+CD25-FOXP3- Ki67+

Table S6: Markers used to define cell populations from flow cytometry

ID	Type	Age collected	Age diagnosed diabetes
HC1	Healthy	11	-
HC2	Healthy	10	-
HC3	Healthy	10	-
HC4	Healthy	11	-
T1D1	Type 1 diabetes	11	11
T1D2	Type 1 diabetes	11	11
T1D3	Type 1 diabetes	11	11
T1D4	Type 1 diabetes	11	11
T1D5	Type 1 diabetes	11	1

Table S7: Controls for flow cytometry

5 MATERIAL AND METHODS

Subjects

The study was conducted in accordance with the Declaration of Helsinki and all subjects, or their parents, gave informed consent for DNA extraction, genetic testing and sample storage in the Genetic Beta Cell Research Bank (https://www.diabetesgenes.org/current-research/genetic-beta-cell-research-bank/). The study was approved by the Wales Research Ethic Committee 5 Bangor (REC 17/WA/0327, IRAS project ID 231760). Individuals with neonatal diabetes (NDM: diagnosed <6 months) or early-onset diabetes (diagnosed <5 years) were recruited by their clinicians to the Exeter Genomics Laboratory for monogenic diabetes genetic testing through a dedicated referral form (https://www.diabetesgenes.org/download/3564/?tmstv=1715331423). Genetic ancestry was assigned using Procrustes analysis and random forest classification [5].

Genetic testing

Whole-genome sequencing of DNA extracted from peripheral blood leukocytes was completed on 181 individuals with NDM and 95 with early-onset diabetes (n=70 with Illumina HiSeq X10 [illumina, USA] n=206 with BGISeq-500 [BGI Europe, Poland]). The resulting sequence reads were aligned to the GRCh38.p14 assembly using BWA MEM version 0.7.15 [6], and processed with our bespoke pipeline based on GATK best practices (Picard version 2.7.1 and GATK version 3.7 [7]). All samples had mean coverage >30x and >95% coverage at >20x. Variants were annotated using Alamut batch standalone version 1.11 (SOPHiA Genetics, Switzerland). We separately analysed the 19,435 coding and 59,251 non-coding genes as annotated in the GENCODE reference [8]. We performed direct Sanger sequencing of *RNU4ATAC* and *RNU6ATAC* following PCR to identify further affected individuals and to perform confirmatory and family member testing. The primer sequences are provided below:

Primer ID	Sequence	Region amplified (Hg38)
RNU6ATAC forward	AGACAGTTCTTCCCGCCTTC	chr9:134164335-134164925
RNU6ATAC reverse	GGGGTGCAGGTTGTAGTGAG	
RNU4ATAC forward	GTGGAGGCTGGAGGTAAGC	chr2:121530763-121531119
RNU4ATAC reverse	GACACTAAAACCACGCGTCTTG	

Islet autoantibody testing

Serum Islet autoantibody testing (GADA, IA-2A, ZnT8A) was performed by Enzyme linked immunosorbent assay (ELISA), at Exeter Clinical Laboratory International (https://www.exeterlaboratory.com/blood-sciences/). This laboratory is UKAS accredited (ISO 15189:2012) and participates in UKNEQAS accreditation and the Islet Autoantibody Standardisation Program.

Transcriptomics

RNA was extracted from whole blood samples preserved in Tempus solution (Applied Biosystems, USA). RNA-seq experiments were performed at the Exeter Sequencing Facility (University of Exeter, UK). Briefly, 100ng RNA was prepared using the Illumina RiboZero library preparation kit to manufacturer's instructions, followed by paired-end 100bp sequencing on an Illumina NovaSeq (Illumina, USA). The resulting reads were then aligned to the GRCh38 reference using STAR v2.7.11b [9]. Transcripts were quantified using RSEM v1.33 [10] and intron retention was measured using SpliceWiz v1.10.1[11]. Weighted gene co-expression analysis was performed using the WGCNA[12] package in R to identify gene modules that were significantly correlated with affected status for minor spliceosomeopathies. Gene ontology [13,14] and KEGG pathway analysis [15] was then performed on the gene sets for these modules using the clusterProfiler package. Differential splicing analysis was performed on the intron retention values from SpliceWiz using EdgeR v4.6.2 [16].

Methylation analysis

To investigate the original immune cell components of cohort whole blood DNA samples we performed methylation array analysis using the EPIC v2 array on an iScan instrument (Illumina, USA). Initial QC was performed to ensure median methylated and unmethylated signal intensities were greater than 1000, that median bisulfite conversion percentage was above 80 and that sample sex determined from methylation data matched reported sex. Normalisation was performed using the Subset-quantile Within Array Normalisation (SWAN) method with the Minfi package [17]. Finally, blood cell deconvolution was performed using the deconvolution tool and optimized blood cell methylation reference libraries from Salas et al.[18]. This method estimates the proportion of cells in the original whole blood from 12 immune cell subsets (neutrophils, eosinophils, basophils, monocytes, naïve and memory B cells, naïve and memory CD4+ and CD8+ T cells, natural killer, and T regulatory cells) using measurement of methylation of cell-type specific loci.

Flow cytometry

Leukocyte populations were characterised from fresh whole blood (collected in 2.7 mL EDTA tubes) using five multi-parameter flow cytometry panels previously validated and assessed for technical reproducibility across multiple laboratories [19-21]. In brief, surface marker staining (Table S5) of 100-200µL well-mixed fresh whole blood was performed for 45 min at RT, followed by red blood cell lysis during 8 min at RT (10x BD FACS lysing solution diluted in ddH2O, BD Biosciences, US). Staining for the lineage flow cytometry panel was conducted on a BD Trucount tube (BD Biosciences, US) enabling the calculation of absolute cell numbers alongside cell frequencies. The lineage tube was vortexed to ensure homogenisation and left on ice until acquisition (lyse, no wash). All other staining panel tubes were centrifuged at 500g for 5 min, cells were washed in 2 mL of FACS buffer [1x PBS (Invitrogen, US) containing 0.2% BSA (Sigma-Aldrich, US) and 2 mM EDTA (Sigma-Aldrich, US)] by centrifugation at 500g for 5 min, resuspended in 200 µL of FACS buffer and kept on ice until acquisition. Intracellular marker staining of 100 µL of fresh whole blood consisted of two 15 min incubations at RT with CD45RA-BV785 and 10 µL of fixative reagent (buffer 1 from PerFix-nc kit, Beckman Coulter, USA), respectively. Permeabilising reagent (buffer 2 from PerFix-nc kit, Beckman Coulter, USA) was added to the intracellular master mix of fluorescently labelled antibodies prepared previously, and cells stained intracellularly for 60 min at RT. After incubation, 3 mL of plain 1x PBS

(Invitrogen, USA) was added for 5 min followed by centrifugation at 500g for 5 min. Cells were washed in 3 mL of 1x R3 reagent (10x buffer 3 diluted in ddH2O from PerFix-nc kit, Beckman Coulter, US), resuspended in 200 μL of 1x R3 reagent and kept on ice until acquisition. Cells were acquired on a BD LSRFortessa™ Cell Analyzer. A minimum of 400,000 events were acquired per sample per staining panel. Phenotypes used to define cell subtypes are provided in table S6. The resulting flow cytometry data was analysed using FlowJo (BD Biosciences, USA) and compared to data from 4 age matched healthy controls and 5 age-matched T1D controls (table S7).

Supplementary References:

1. Bai, R., Yuan, M., Zhang, P., Luo, T., Shi, Y., and Wan, R. (2024). Structural basis of U12-type intron engagement by the fully assembled human minor spliceosome. Science 383, 1245–1252. https://doi.org/10.1126/science.adn7272.

2. Bai, R., Wan, R., Wang, L., Xu, K., Zhang, Q., Lei, J., and Shi, Y. (2021). Structure of the activated human minor spliceosome. Science 371, eabg0879. https://doi.org/10.1126/science.abg0879.

3. Moyer, D.C., Larue, G.E., Hershberger, C.E., Roy, S.W., and Padgett, R.A. (2020). Comprehensive database and evolutionary dynamics of U12-type introns. Nucleic Acids Res 48, 7066–7078. https://doi.org/10.1093/nar/gkaa464.

4. Newman, A.M., Steen, C.B., Liu, C.L., Gentles, A.J., Chaudhuri, A.A., Scherer, F., Khodadoust, M.S., Esfahani, M.S., Luca, B.A., Steiner, D., et al. (2019). Determining cell type abundance and expression from bulk tissues with digital cytometry. Nat Biotechnol 37, 773–782. https://doi.org/10.1038/s41587-019-0114-2.

5. De Franco E, Russ-Silsby J, Batage MH, Thomas L, Wakeling M, Johnson M, et al. Population labels can be generated directly from targeted next-generation sequencing data. 2024; https://doi.org/10.21203/rs.3.rs-5282595/v1

6. Li, H., and Durbin, R. (2009). Fast and accurate short read alignment with Burrows–Wheeler transform. Bioinformatics 25, 1754–1760. https://doi.org/10.1093/bioinformatics/btp324.

7. Auwera G van der, O'Connor BD. Genomics in the cloud : using Docker, GATK, and WDL in Terra. First edition. Sebastopol, CA: O'Reilly Media; 2020.

8. Mudge, J.M., Carbonell-Sala, S., Diekhans, M., Martinez, J.G., Hunt, T., Jungreis, I., Loveland, J.E., Arnan, C., Barnes, I., Bennett, R., et al. (2024). GENCODE 2025: reference gene annotation for human and mouse. Nucleic Acids Res *53*, D966–D975. https://doi.org/10.1093/nar/gkae1078.

9. Dobin, A., Davis, C.A., Schlesinger, F., Drenkow, J., Zaleski, C., Jha, S., Batut, P., Chaisson, M., and Gingeras, T.R. (2013). STAR: ultrafast universal RNA-seq aligner. Bioinformatics 29, 15–21. https://doi.org/10.1093/bioinformatics/bts635.

10. Li, B., and Dewey, C.N. (2011). RSEM: accurate transcript quantification from RNA-Seq data with or without a reference genome. BMC Bioinformatics 12, 323. https://doi.org/10.1186/1471-2105-12-323.

11. Wong, A.C.H., Wong, J.J.-L., Rasko, J.E.J., and Schmitz, U. (2024). SpliceWiz: interactive analysis and visualization of alternative splicing in R. Brief Bioinform 25, bbad468. https://doi.org/10.1093/bib/bbad468.

12. Langfelder, P., and Horvath, S. (2008). WGCNA: an R package for weighted correlation network analysis. BMC Bioinformatics 9, 559. https://doi.org/10.1186/1471-2105-9-559.

13. Ashburner, M., Ball, C.A., Blake, J.A., Botstein, D., Butler, H., Cherry, J.M., Davis, A.P., Dolinski, K., Dwight, S.S., Eppig, J.T., et al. (2000). Gene Ontology: tool for the unification of biology. Nat Genet 25, 25–29. https://doi.org/10.1038/75556.

14. Aleksander, S.A., Balhoff, J., Carbon, S., Cherry, J.M., Drabkin, H.J., Ebert, D., Feuermann, M., Gaudet, P., Harris, N.L., Hill, D.P., et al. (2023). The Gene Ontology knowledgebase in 2023. Genetics 224, iyad031. https://doi.org/10.1093/genetics/iyad031.7

15. Kanehisa, M., and Goto, S. (2000). KEGG: kyoto encyclopedia of genes and genomes. Nucleic Acids Res *28*, 27–30. https://doi.org/10.1093/nar/28.1.27.

16. Chen, Y., Chen, L., Lun, A.T.L., Baldoni, P.L., and Smyth, G.K. (2025). edgeR v4: powerful differential analysis of sequencing data with expanded functionality and improved support for small counts and larger datasets. Nucleic Acids Res 53, gkaf018. https://doi.org/10.1093/nar/gkaf018.

17. Aryee, M.J., Jaffe, A.E., Corrada-Bravo, H., Ladd-Acosta, C., Feinberg, A.P., Hansen, K.D., and Irizarry, R.A. (2014). Minfi: a flexible and comprehensive Bioconductor package for the analysis of Infinium DNA methylation microarrays. Bioinformatics 30, 1363–1369. https://doi.org/10.1093/bioinformatics/btu049.

18. Salas, L.A., Zhang, Z., Koestler, D.C., Butler, R.A., Hansen, H.M., Molinaro, A.M., Wiencke, J.K., Kelsey, K.T., and Christensen, B.C. (2022). Enhanced cell deconvolution of peripheral blood using DNA methylation for high-resolution immune profiling. Nat Commun 13, 761. https://doi.org/10.1038/s41467-021-27864-7.

19. Yang JHM, Ward-Hartstonge KA, Perry DJ, Blanchfield JL, Posgai AL, Wiedeman AE, et al. Guidelines for standardizing T-cell cytometry assays to link biomarkers, mechanisms, and disease outcomes in type 1 diabetes. Eur J Immunol. 2022 Mar;52(3):372–88.

20. Sanz, I., Wei, C., Jenks, S.A., Cashman, K.S., Tipton, C., Woodruff, M.C., Hom, J., and Lee, F.E.-H. (2019). Challenges and Opportunities for Consistent Classification of Human B Cell and Plasma Cell Populations. Front Immunol 10, 2458. https://doi.org/10.3389/fimmu.2019.02458.

21. Yang, J.H.M., Khatri, L., Mickunas, M., Williams, E., Tatovic, D., Alhadj Ali, M., Young, P., Moyle, P., Sahni, V., Wang, R., et al. (2019). Phenotypic Analysis of Human Lymph Nodes in Subjects With New-Onset Type 1 Diabetes and Healthy Individuals by Flow Cytometry. Front Immunol 10, 2547. https://doi.org/10.3389/fimmu.2019.02547.
