# Supplementary material for: Bi-allelic variants in the non-protein-coding minor spliceosome components RNU6ATAC and RNU4ATAC cause syndromic monogenic autoimmune diabetes
Source: Am J Hum Genet. 2026 Mar 20;113(4):877–87. doi: 10.1016/j.ajhg.2026.02.017 (PMC13087456; doi:10.1016/j.ajhg.2026.02.017)
Supplement: Document S2. Article plus supplemental information [file mmc3.pdf]

Bi-allelic variants in the non-protein-coding minor spliceosome components *RNU6ATAC* and *RNU4ATAC* cause syndromic monogenic autoimmune diabetes

Graphical abstract

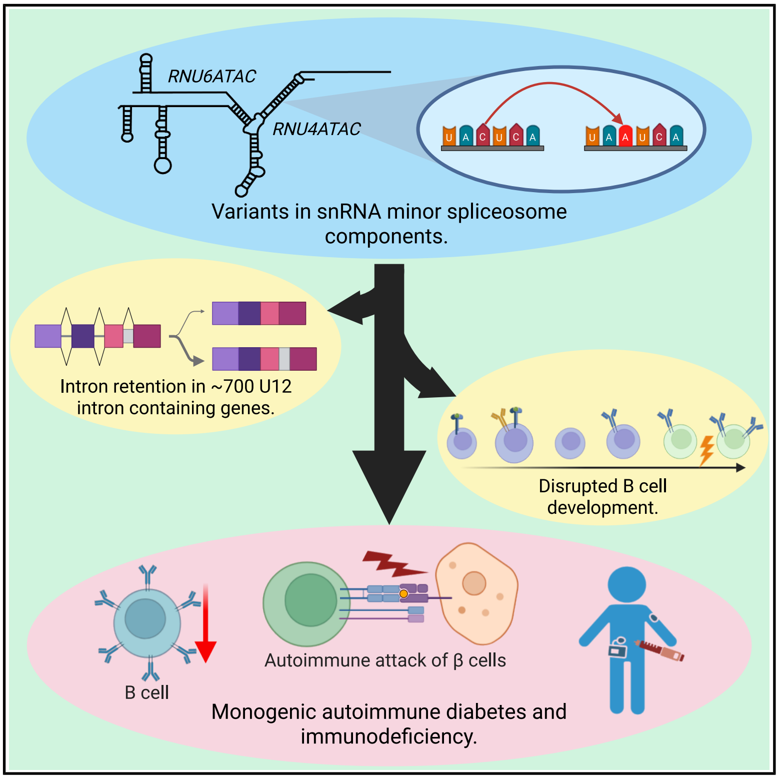

Authors

Matthew B. Johnson, James Russ-Silsby, Paul A. Blair, ..., Kashyap A. Patel, Andrew T. Hattersley, Elisa De Franco

Correspondence

e.de-franco@exeter.ac.uk

We show that bi-allelic variants in the minor spliceosome snRNAs *RNU6ATAC* and *RNU4ATAC* cause early-onset autoimmune diabetes with immune dysregulation. We define a shared mechanism involving U12 intron retention and impaired B cell development, expanding the phenotypic spectrum of RNU4ATAC-opathy and implicating minor splicing in beta cell autoimmunity.

Johnson et al., 2026, The American Journal of Human Genetics *113*, 877–887
April 2, 2026 © 2026 The Author(s). Published by Elsevier Inc. on behalf of American Society of Human Genetics.
https://doi.org/10.1016/j.ajhg.2026.02.017

Bi-allelic variants in the non-protein-coding minor spliceosome components *RNU6ATAC* and *RNU4ATAC* cause syndromic monogenic autoimmune diabetes

Matthew B. Johnson,[1,3] James Russ-Silsby,[1,3] Paul A. Blair,[2] Molly Govier,[1] Georgia Bonfield,[1] Clara Domingo-Vila,[2] EXE-T1D consortium,[1] ATAC clinical consortium, Matthew N. Wakeling,[1] Richard A. Oram,[1] Sarah E. Flanagan,[1] Timothy I.M. Tree,[2] Kashyap A. Patel,[1] Andrew T. Hattersley,[1] and Elisa De Franco[1,*]

Summary

Non-protein-coding genes are emerging as critical contributors to the etiology of rare diseases, providing key insights into human biology and uncovering novel disease mechanisms. We identified 7 individuals from 4 families with early-onset diabetes (diagnosed aged <5 years) and immune dysregulatory features caused by bi-allelic variants in *RNU6ATAC*. *RNU6ATAC* encodes a small nuclear RNA (snRNA) that acts as a catalytic component of the minor spliceosome, a protein-RNA complex that mediates the splicing of ~700 genes containing U12/minor-type introns. Variant screening of the other 64 minor spliceosome genes in 276 infants with diabetes identified 12 unrelated individuals with bi-allelic disease-causing variants in *RNU4ATAC*. Bi-allelic pathogenic *RNU4ATAC* variants are known to cause a variable spectrum of clinical features, which until now did not include diabetes. Clinically, 12/19 *RNU6ATAC/RNU4ATAC* affected individuals had additional immune dysregulatory features, and 50% of individuals tested were islet-autoantibody positive, strongly supporting an autoimmune etiology for their diabetes. RNA sequencing (RNA-seq) in 3 individuals with bi-allelic *RNU6ATAC* variants showed a pattern of intron retention in U12-intron-containing genes similar to that seen in *RNU4ATAC* individuals ($n = 3$), supporting a shared disease mechanism. Analysis of affected individuals' transcriptomic, methylation, and immune data revealed impaired B cell development and maturation. We conclude that bi-allelic *RNU6ATAC* variants cause a syndrome of early-onset autoimmune diabetes and immune dysregulation. We further show that infancy-onset diabetes is a feature of RNU4ATAC-opathy. Our work highlights the important role of two snRNAs critical to minor spliceosome function in immune system regulation, providing insights into the pathogenesis of autoimmune diabetes.

Uncovering genetic causes of human disease provides key insights into the regulation of core biological processes. Despite significant advances ever since the introduction of exome and genome sequencing, up to half of individuals affected by a rare disease remain without a genetic diagnosis.[1,2] Variants in the non-protein-coding genome—including regulatory elements such as promoters, enhancers, and untranslated regions—may account for a considerable fraction of this diagnostic gap.[3–5] The recent discovery that ReNU syndrome (MIM: 620851)—caused by variants in the non-protein-coding gene *RNU4-2* (MIM: 620823)—explains ~0.4% of individuals with undiagnosed neurodevelopmental delay has highlighted the importance of non-protein-coding genes in rare disease.[6–8]

Here, we report the identification of variants in the non-protein-coding gene *RNU6ATAC* (MIM: 601429), encoding a catalytic component of the minor spliceosome, as the genetic cause of a syndrome defined by early-onset autoimmune diabetes, hypogammaglobulinemia, and additional immune dysregulation. Through screening of the genes encoding the other minor spliceosome components, we also extend the phenotype associated with bi-allelic variants in another non-coding minor spliceosome component, *RNU4ATAC* (MIM: 601428), to include early-onset autoimmune diabetes.

To identify genetic causes of autoimmune diabetes and immune dysregulation, we performed genome sequencing in three consanguineous individuals with infancy-onset diabetes (diagnosed at 13, 17, and 36 weeks) and hypogammaglobulinemia. All known genetic causes of infancy-onset diabetes had been previously excluded. No shared genes with ultra-rare homozygous coding variants (gnomAD v.4.1.0[9] minor-allele frequency [MAF] $< 1 \times 10^{-5}$) in all 3 individuals were identified. We next looked for ultra-rare homozygous variants in non-coding genes. This identified a single common gene, the small nuclear RNA (snRNA) *RNU6ATAC* (Figure 1A; Table 1). All three individuals had a different *RNU6ATAC* homozygous variant that was absent from gnomAD v.4.1.0 (~75,000 individuals) and ClinVar.[10] The affected sibling of one of the individuals (individual A.II-2) was also homozygous for the *RNU6ATAC* rare variant. *RNU6ATAC* variants have not previously been reported as causing human monogenic disease.

To identify further individuals, we screened existing genome sequencing data from 276 individuals with

[1]Clinical and Biomedical Science, Faculty of Health and Life Sciences, University of Exeter, Exeter, UK; [2]Department of Immunobiology, King's College London, London, UK
[3]These authors contributed equally
*Correspondence: e.de-franco@exeter.ac.uk
https://doi.org/10.1016/j.ajhg.2026.02.017.
© 2026 The Author(s). Published by Elsevier Inc. on behalf of American Society of Human Genetics.
This is an open access article under the CC BY license (http://creativecommons.org/licenses/by/4.0/).

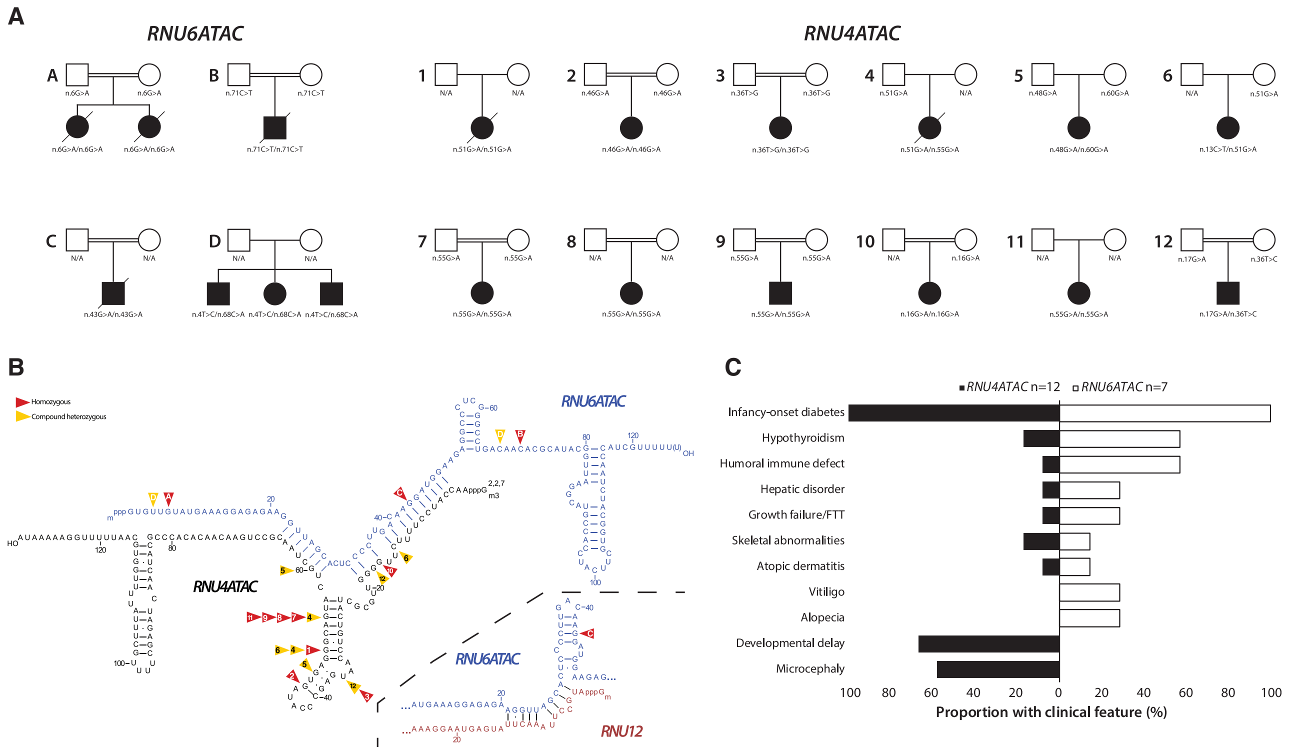

Figure 1. Genetic and clinical information for the RNU6ATAC and RNU4ATAC cohorts
(A) Partial pedigrees of the individuals with *RNU6ATAC* and *RNU4ATAC* bi-allelic variants.
(B) Secondary structure of RNU4ATAC and RNU6ATAC snRNA duplex with variant positions highlighted and number/letter by the affected individual. Heterozygous variants identified in *trans* with another pathogenic variant are shown with yellow arrows. Homozygous variants are shown with red arrows. Hydrogen bonds between bases in stem I and stem II are shown with lines. The image was adapted from Almentina Ramos Shidi et al.[11]
(C) Tornado plot showing clinical features in *RNU6ATAC* vs. *RNU4ATAC* cohorts.

neonatal diabetes (diagnosed aged <6 months) or early-onset diabetes with additional autoimmune disease (both diagnosed aged <5 years) in whom known causes of monogenic diabetes had been excluded.[12] This analysis identified 3 further individuals from 1 family with compound heterozygous variants in *RNU6ATAC* (Figure 1A; Table 1), taking the total to 7 individuals from 4 families. Sanger sequencing of *RNU6ATAC* (see the supplemental material and methods for primer sequences and amplified region) in 196 individuals with neonatal diabetes of unknown cause who did not have genome sequencing data did not identify any additional individuals.

RNU6ATAC forms part of the catalytic core of the minor spliceosome.[13,14] This fundamental and highly evolutionarily conserved protein-RNA complex mediates the excision of introns containing the U12-type sequence motif (<0.5% of all human introns) to create mature mRNA.[15] Disruption of the spliceosome causes intron retention that leads to downstream impacts, including transcript loss due to nonsense-mediated decay or disruption to protein function through protein truncation or protein elongation.[16]

We next investigated if bi-allelic variants in the other 64 genes encoding components of the minor spliceosome were implicated in the etiology of early-onset diabetes (Table S1).[14,17] We screened for ultra-rare variants in these genes using the same genome sequencing dataset used for the *RNU6ATAC* analysis described above. This identified 7 unrelated individuals with homozygous or compound heterozygous variants in *RNU4ATAC* (Figure 1A; Table 2). We did not identify ultra-rare bi-allelic variants in any of the 63 remaining genes. We then Sanger sequenced *RNU4ATAC* in 196 individuals with neonatal diabetes of unknown cause who had not undergone genome sequencing and identified 5 additional individuals. In total, we identified 12 individuals with bi-allelic likely pathogenic/pathogenic *RNU4ATAC* variants in our early-onset diabetes cohort.

RNU4ATAC encodes an snRNA that binds directly to *RNU6ATAC* to stabilize the pre-catalytic configuration of the minor spliceosome, sequestering *RNU6ATAC*'s catalytic elements.[13,14] Recessively inherited variants in *RNU4ATAC* are a known cause of "RNU4ATAC-opathies,"[18] encompassing a highly variable clinical spectrum that commonly includes microcephaly, developmental delay, and intra-uterine growth restriction (IUGR) and can include immune dysregulatory features. Diabetes has not been reported as a feature of RNU4ATAC-opathies.

In total, we therefore identified 19 individuals from 16 families with defects in 2 minor spliceosome snRNAs

Table 1. Demographic, genetic, and clinical features of individuals with bi-allelic *RNU6ATAC* disease-causing variants

Individual	A.II-1	A.II-2	B.II-1	C.II-1	D.II-1	D.II-2	D.II-3
Age at last assessment (years[a])	0.3 (deceased)	0.3 (deceased)	0.3 (deceased)	5	4	14	9
Sex	female	female	male	male	male	female	male
Genetic ancestry	EAS	EAS	SAS	MID	MID	MID	MID
BW *Z* score	−2.26	−3.17	−1.72	N/R	0.06	0.06	N/R
Age diagnosed with diabetes (weeks)	13	11	17	36	5	110	260
Glucose at diagnosis (mmol/L)	28.3	33	41	N/R	25	25	N/R
HbA1c (mmol/mol)	N/A[b]	N/A[b]	N/A[b]	N/R	111	108	118
Insulin dose U/kg/day	N/A, on infusion before death	N/A, on infusion before death	1.5	N/R	0.7	N/R	N/R
Islet autoantibodies (titer, threshold, IU)	N/A	N/A	GADA positive (52, >11)	N/R	negative	negative	GADA positive (98, >11)
Immune dysregulatory features	sepsis, atopic dermatitis, B cell lymphopenia, low IgA, low IgG, hypothyroidism	B cell lymphopenia, hypothyroidism	agammaglobulinemia	hypogammaglobulinemia, immunodeficiency	thyroiditis (1.5 years), alopecia (3.5 years)	N/R	alopecia and vitiligo (4.5 years), thyroiditis (4 years)
Microcephaly	no	no	no	N/R	no	no	no
Developmental delay	N/A, died early infancy	N/A, died early infancy	NA, died early infancy	N/R	no	no	no
Additional features	diarrhea, facial dysmorphism	jaundice, elevated unconjugated bilirubin	none reported	epiphyseal dysplasia, elevated liver enzymes	none reported	severe growth retardation (not GH deficient), delayed puberty	none reported
RNU6ATAC variant	n.[6G>A]; [6G>A] (GenBank: NR_023344.1)	n.[6G>A]; [6G>A] (GenBank: NR_023344.1)	n.[71C>T]; [71C>T] (GenBank: NR_023344.1)	n.[43G>A]; [43G>A] (GenBank: NR_023344.1)	n.[4T>C]; [68C>A] (GenBank: NR_023344.1)	n.[4T>C]; [68C>A] (GenBank: NR_023344.1)	n.[4T>C]; [68C>A] (GenBank: NR_023344.1)
Genomic co-ordinate (Hg38)	g.[134164559C>T]; [134164559C>T] (GenBank: NC_000009.12)	g.[134164559C>T]; [134164559C>T] (GenBank: NC_000009.12)	g.[134164494G>A]; [134164494 G>A] (GenBank: NC_000009.12)	g.[134164522C>T]; [134164522C>T] (GenBank: NC_000009.12)	g.[134164561A>G]; [134164497G>T] [GenBank: NC_000009.12]	g.[134164561A>G]; [134164497G>T] [GenBank: NC_000009.12]	g.[134164561A>G]; [134164497G>T] [GenBank: NC_000009.12]

BW, birthweight; N/A, not applicable; N/R, not recorded; EAS, East Asian; SAS, South Asian; MID, Middle Eastern; F, female; M, male; GADA, glutamic acid decarboxylase; GH, growth hormone.
[a]Presented as years and fractions of a year.
[b]Due to the presence of fetal hemoglobin, HbA1c measurement is not reliable in the first 6 months of life.

Table 2. Demographic, genetic, and clinical features of individuals with bi-allelic *RNU4ATAC* disease-causing variants

Individual	1.II-1	2.II-1	3.II-1	4.II-1	5.II-1	6.II-1	7.II-1	8.II-1	9.II-1	10.II-1	11.II-1	12.II-1
Age at last assessment (years[a])	6.8 (deceased)	12 (deceased)	0.9	1.4 (deceased)	8.7	11	5.9	9.5	1.0	0.8	1.3	0.4
Sex	F	F	F	F	F	F	F	F	F	F	F	M
Genetic ancestry	MID	OTH	MID	EAS	OTH	AFR	MID	OTH	EAS	MID	SAS	MID
BW Z score	−3.07	−3.04	−4.39	−4.29	−1.71	−0.97	1.14	−2.15	−1.56	−0.98	−0.14	−2.71
Age diagnosed with diabetes (weeks)	20	61	8	10	21	10	51	26	40	20	20	1
Glucose at diagnosis (mmol/L)	38.1	25	21	20.3	39	32	N/R	33	55	33	44	24
HbA1c (mmol/mol)	64	73	N/A	47.5	43.2	50	61	N/A	57	N/A	90	45
Insulin dose U/kg/day	1.5	3	0.82	1.5	0.8	0.72	N/R	1.2	1.0	1	1.4	0.5
Islet autoantibodies (titer, threshold for positivity)	negative	negative	N/A	negative	GADA positive (93, >11)	GADA positive (1,581, >11)	GADA positive (657, >11)	N/A	N/A	N/A	N/A	N/A
Immune dysregulatory features	recurrent infections, IgA deficiency	recurrent infections, myelodysplastic syndrome	N/R	N/R	raised immature granulocytes	severe atopic dermatitis, autoimmune hypothyroidism	N/R	recurrent infections	N/R	N/R	N/R	autoimmune hypothyroidism
Microcephaly	yes	yes	yes	yes	N/R	yes	yes	yes	yes	N/R	yes	N/R
Developmental delay	yes	yes	N/R	yes	yes	yes	yes	yes	N/R	N/R	yes	N/R
Additional clinical features	hip dislocation, muscle weakness, respiratory failure, seizures	triple X syndrome, insulin resistance, muscle weakness, dysmorphic features, growth retardation, diabetic nephropathy	facial dysmorphism, peaked nose, small head, downward slanting palpebral fissures, small mandible	muscle weakness, corpus callosum agenesis, bilateral knee dislocation	muscle weakness, epilepsy, cholestasis and direct hyperbilirubinemia (resolved), ASD, congenital cataracts, sensorineural hearing loss	loose skin folds, laryngomalacia tracheomalacia, multiple epiphyseal dysplasia, high myopia, alternating exotropia	high arched palate, abnormal object eye tracking, dysmorphic features	epilepsy, musculoskeletal abnormalities, premature menarche (9 years, suppressed), mitral valve prolapse; older sister affected with microcephaly and diabetes (11 m), died age 15 m	N/R	N/R	dysmorphism	muscle weakness
RNU4ATAC variant	n.[51G>A]; [51G>A] (GenBank: NR_023343.1)	n.[46G>A]; [46G>A] (GenBank: NR_023343.1)	n.[36T>G]; [36T>G] (GenBank: NR_023343.1)	n.[51G>A]; [55G>A] (GenBank: NR_023343.1)	n.[48G>A]; [60G>A] (GenBank: NR_023343.1)	n.[13C>T]; [51G>A] (GenBank: NR_023343.1)	n.[55G>A]; [55G>A] (GenBank: NR_023343.1)	n.[55G>A]; [55G>A] (GenBank: NR_023343.1)	n.[55G>A]; [55G>A] (GenBank: NR_023343.1)	n.[16G>A]; [16G>A] (GenBank: NR_023343.1)	n.[55G>A]; [55G>A] (GenBank: NR_023343.1)	n.[17G>A]; [36T>C] (GenBank: NR_023343.1)
Genomic co-ordinate (Hg38)	g.[121530930G>A]; [121530930G>A] (GenBank: NC_000002.12)	g.[121530925G>A]; [121530925G>A] (GenBank: NC_000002.12)	g.[121530915T>G]; [121530915T>G] (GenBank: NC_000002.12)	g.[121530930G>A]; [121530934G>A] (GenBank: NC_000002.12)	g.[121530927G>A]; [121530939G>A] (GenBank: NC_000002.12)	g.[121530892C>T]; [121530930G>A] (GenBank: NC_000002.12)	g.[121530934G>A]; [121530934G>A] (GenBank: NC_000002.12)	g.[121530934G>A]; [121530934G>A] (GenBank: NC_000002.12)	g.[121530934G>A]; [121530934G>A] (GenBank: NC_000002.12)	g.[121530895G>A]; [121530895G>A] (GenBank: NC_000002.12)	g.[121530934G>A]; [121530934G>A] (GenBank: NC_000002.12)	g.[121530896G>A]; [121530915T>C] (GenBank: NC_000002.12)

BW, birthweight; N/A, not applicable; N/R, not recorded; MID, Middle Eastern; OTH, other; EAS, East Asian; AFR, African; SAS, South East Asian; ASD, atrial septal defect; F, female; M, male; GADA, glutamic acid decarboxylase autoantibody.

[a]Presented as years and fractions of a year.

and early-onset diabetes (Figures 1A and 1B): 7 individuals from 4 families with bi-allelic *RNU6ATAC* variants (Table 1, denoted by letters) and 12 unrelated individuals with bi-allelic *RNU4ATAC* variants (Table 2, denoted by numbers). Variant testing confirmed carrier status in all the parents available for testing ($n = 19$).

Bi-allelic *RNU6ATAC* variants caused early-onset diabetes (median onset: 17 weeks) with additional immune dysregulation in 6/7 individuals (Table 1). Common features included humoral immune defects (4/7; B cell lymphopenia and a/hypogammaglobulinemia) and autoimmunity (4/7; hypothyroidism [$n = 4$], alopecia [$n = 2$], and vitiligo [$n = 1$]). Cholestatic jaundice and elevated liver enzymes were observed in two individuals. Among individuals with reported gestation, 3/5 had IUGR (Z score < −1.28[19]).

Individuals with bi-allelic *RNU4ATAC* variants often displayed classic RNU4ATAC-opathy features,[18] including microcephaly, IUGR, and developmental delay, which were seen in 10/12 individuals (Table 2). Six had immune dysregulation (including hypothyroidism [$n = 2$] and recurrent infections [$n = 3$]). Autoimmunity (e.g., Addison's disease and autoimmune hypothyroidism) has been reported in some individuals with RNU4ATAC-opthies,[20] including a single individual with type 1 diabetes (T1D) and Addison's disease.[21] In our cohort, 12 unrelated individuals had early-onset diabetes (median onset: 20 weeks), confirming that diabetes is part of the RNU4ATAC-opathy spectrum. Diabetes is unlikely to be attributable to specific variants in *RNU4ATAC*, as 12/15 variants were previously reported to cause RNU4ATAC-opathies in individuals who were not diagnosed with diabetes.[10]

There was substantial overlap in clinical features between individuals with variants in *RNU6ATAC* and *RNU4ATAC* but also some notable differences (Figure 1C). In our cohort, variants in both genes were associated with early-onset diabetes and immune dysregulation. Developmental abnormalities were common in individuals with *RNU4ATAC* variants, consistent with previous reports, but not in those with variants in *RNU6ATAC*, although later-onset developmental issues in the latter group cannot be excluded. A recent study identified an individual with compound heterozygous ultra-rare *RNU6ATAC* variants of unknown clinical significance (n.[36T>G]; [28C>T] [GenBank: NR_023344.1]) who had IUGR, postnatal growth failure, microcephaly, epilepsy, intellectual disability, and ataxia but no diabetes.[22]

To investigate the diabetes mechanism, we first performed islet autoantibody testing on available serum samples ($n = 4$ *RNU6ATAC* and $n = 6$ *RNU4ATAC*). Five of the ten individuals (50%) were positive for GADA (glutamic acid decarboxylase) antibodies (threshold is >97.5th centile of the non-diabetic population). This rate of positive islet autoantibodies is similar to that seen in age-matched individuals with T1D and monogenic autoimmune diabetes,[23,24] indicating that the diabetes in these individuals is likely driven by islet autoimmunity. Furthermore, all individuals with available clinical data presented in infancy/early-childhood with very high glucose values (median: 32 mmol/L, interquartile range [IQR]: 25–39, $n = 15$, Table 1) and were insulin treated with full replacement doses (median: 1.0 U/kg/day, IQR: 0.7–1.5, $n = 13$, Table 1), indicative of profound and rapid loss of endogenous insulin secretion.

To assess the impact of the identified *RNU6ATAC* variants, we performed whole-blood RNA sequencing (RNA-seq) on samples from 3 individuals with bi-allelic variants and compared intron retention to our individuals with bi-allelic *RNU4ATAC* variants ($n = 3$), unaffected parents ($n =$ 4 *RNU6ATAC* and 5 *RNU4ATAC*), healthy age-matched control subjects ($n = 4$), and age-matched individuals with early-onset T1D ($n = 4$) (Table S2). This identified significant intron retention in individuals with bi-allelic *RNU6ATAC* variants, similar to the pattern detected in individuals with bi-allelic *RNU4ATAC* variants, in 274 genes (Figure 2A). Most genes with significant intron retention ($n = 258/274$, 94%) were known U12-intron-containing genes listed in the Intron Annotation and Orthology U12 Database (IAOD) (Figure 2B; Table S3).[25] The remaining 16 genes likely represent previously undescribed U12 genes, given we found significant intron retention in individuals with bi-allelic variants in *RNU4ATAC* ($n = 1$), *RNU6ATAC* ($n = 3$), or both ($n = 12$) (Figures 2A and S1; Table S3). The high level of intron retention of U12 genes identified in our cohort was comparable to that previously reported in individuals with RNU4ATAC-opthies.[20,26,27]

To investigate potential mechanisms for autoimmune diabetes and additional immune dysregulatory features, we performed weighted gene co-expression network analysis (WGCNA) on our RNA-seq data.[28] This identified 2 significantly differentially regulated gene modules in individuals with bi-allelic variants in *RNU4ATAC*/*RNU6ATAC* compared to control subjects (Figure 3A). Enrichment analysis through Gene Ontology[29,30] and KEGG[31] converged on significant enrichment for genes involved in B cell signaling, development, or proliferation and innate immune responses, as well as other immune pathways (Figures 3B and S2).

To investigate the impact on B cells, we performed deconvolution analysis of whole-blood genome-wide methylation data from 7 individuals with bi-allelic *RNU6ATAC* variants, 10 individuals with bi-allelic *RNU4ATAC* variants, and 17 age-matched healthy control subjects (Table S4).[32] This showed significantly reduced estimated naive B cells (Figure 4A), while memory B cells and 10 other immune cell subsets did not show significant differences from control subjects (Figure S3). Deconvolution of RNA-seq data into immune cell subsets also showed reduced naive B cells (Figure S4). To validate these findings, we performed in-depth immune profiling on an individual with bi-allelic *RNU4ATAC* variants (individual 6.II-1, Figure 1A), from whom we were able to obtain a fresh whole-blood sample. This showed a striking B cell

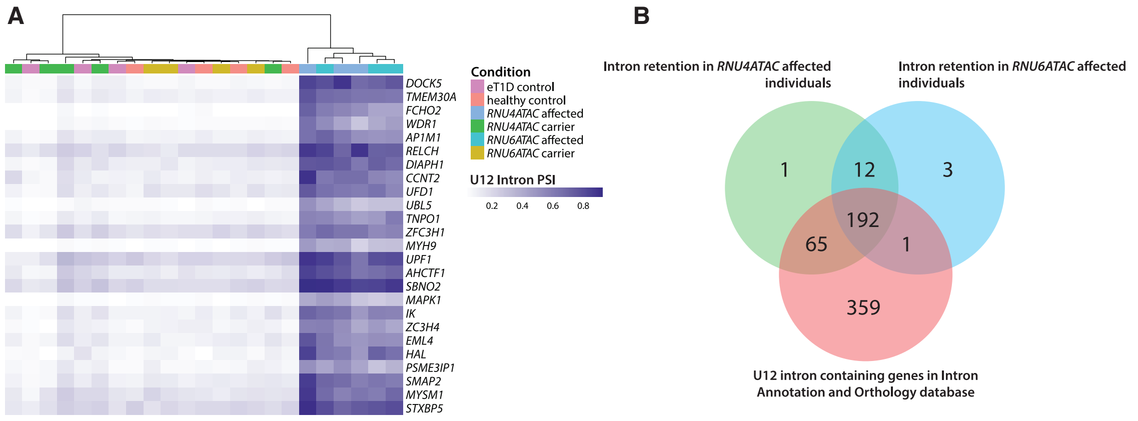

Figure 2. RNA-seq identifies significant intron retention in individuals with variants in *RNU6ATAC* and *RNU4ATAC*

(A) Heatmap showing top 25 genes with significant intron retention. Individuals with variants in *RNU4ATAC* and *RNU6ATAC* cluster together, showing similar profiles of intron retention across U12-intron-containing genes. PSI, percent spliced in.

(B) Venn diagram showing the intersection of known U12-intron-containing genes (IAOD[25]; pink), and empirically defined genes with significant intron retention in whole-blood RNA from individuals with variants in *RNU6ATAC* ($n = 3$, blue) and individuals with variants in *RNU4ATAC* ($n = 3$, green). Thirteen genes were identified that showed significant intron retention in both cohorts but were not present in the IAOD database, most likely reflecting previously undescribed U12 genes.

developmental defect, with reduced naive and memory B cells and increased transitional B cells and antibody-secreting cells vs. age-matched healthy and T1D control subjects (Figures 4B and 4C). This suggests impaired B cell development and maturation, as has previously been seen in some individuals with RNU4ATAC-opathy.[33–35] We also found reduced basophils and increased proliferating $CD8^+$ and $CD4^+$ T cells (i.e., expressing Ki67), while other cell types were similar to healthy and T1D control subjects (Figure S5). The individual was not known to have an infection when the sample was taken, but we are unable to rule out a nascent infection that could explain these findings.

We report bi-allelic pathogenic variants in *RNU6ATAC* as the cause of a genetic syndrome characterized by monogenic autoimmune diabetes with additional immune dysregulation. We also extend the phenotype associated with bi-allelic variants in *RNU4ATAC* to include early-onset autoimmune diabetes. These represent the first causes of monogenic diabetes resulting from pathogenic variants in non-protein-coding genes.

Our results highlight a key role for the minor spliceosome's components *RNU6ATAC* and *RNU4ATAC* in immune regulation. We identified 19 individuals with defects in minor spliceosome components; 7 with the *RNU6ATAC*-associated syndrome and 12 with *RNU4ATAC* variants. All had early-onset diabetes, and 12 (63%) had additional immune dysregulatory features. Using a multi-omic approach, combined with islet autoantibody testing, we provide evidence that the diabetes in these individuals is autoimmune and identify a shared B cell developmental defect across both monogenic disorders.

The *RNU6ATAC* and *RNU4ATAC* variants we have identified are predicted to impact U12 intron splicing through several potential mechanisms. Of the 5 different *RNU6ATAC* variants, 2 variants are predicted to impact U12 intron binding directly (n.4T>C andn.6G>A [GenBank: NR_023344.1]; Figure S6), 1 is within the region of *RNU6ATAC* predicted to bind *RNU4ATAC* (n.43G>A [GenBank: NR_023344.1]) and thus may prevent recruitment of *RNU6ATAC* during spliceosome assembly, and 2 have less clear functional consequences but may induce misfolding or instability of the snRNA or its affinity to other components of the minor spliceosome.[13,17] None of the *RNU6ATAC* variants were within the region that is predicted to bind *RNU12* (MIM: 620204).[36] Of the 10 different *RNU4ATAC* variants, 4 were predicted to affect the *RNU6ATAC* binding region (n.13C>T, n.16G>A, n.17G>A, and n.60G>A [GenBank: NR_023343.1]), with the remaining 6 affecting the 5′ stem loop.[13,17]

Our data support a profound B cell defect resulting from pathogenic variants in *RNU6ATAC* (from affected individuals' RNA-seq and methylation data) or *RNU4ATAC* (from affected individuals' RNA-seq, methylation, and flow cytometry data). This is consistent with humoral immune deficiencies and B cell developmental defects previously reported in some individuals with RNU4ATAC-opathies but without diabetes.[18,33] The role of B cells in the pathogenesis of islet autoimmunity is debated; some evidence points to a direct pathogenic role, while other evidence suggests that B cell dysregulation is secondary to islet autoimmunity.[37] Postmortem pancreases of individuals with the young onset “TIDE1” endotype of T1D show increased insulitic B cells,[38] and B cell depletion with the monoclonal antibody rituximab has shown some success in delaying T1D progression.[39] However, an individual with absent B cells due to X-linked agammaglobulinemia (XLA) developed autoimmune diabetes, implying that B cells or autoantibodies are not required for diabetes development.[40] Further study of individuals with both B cell defects and autoimmune

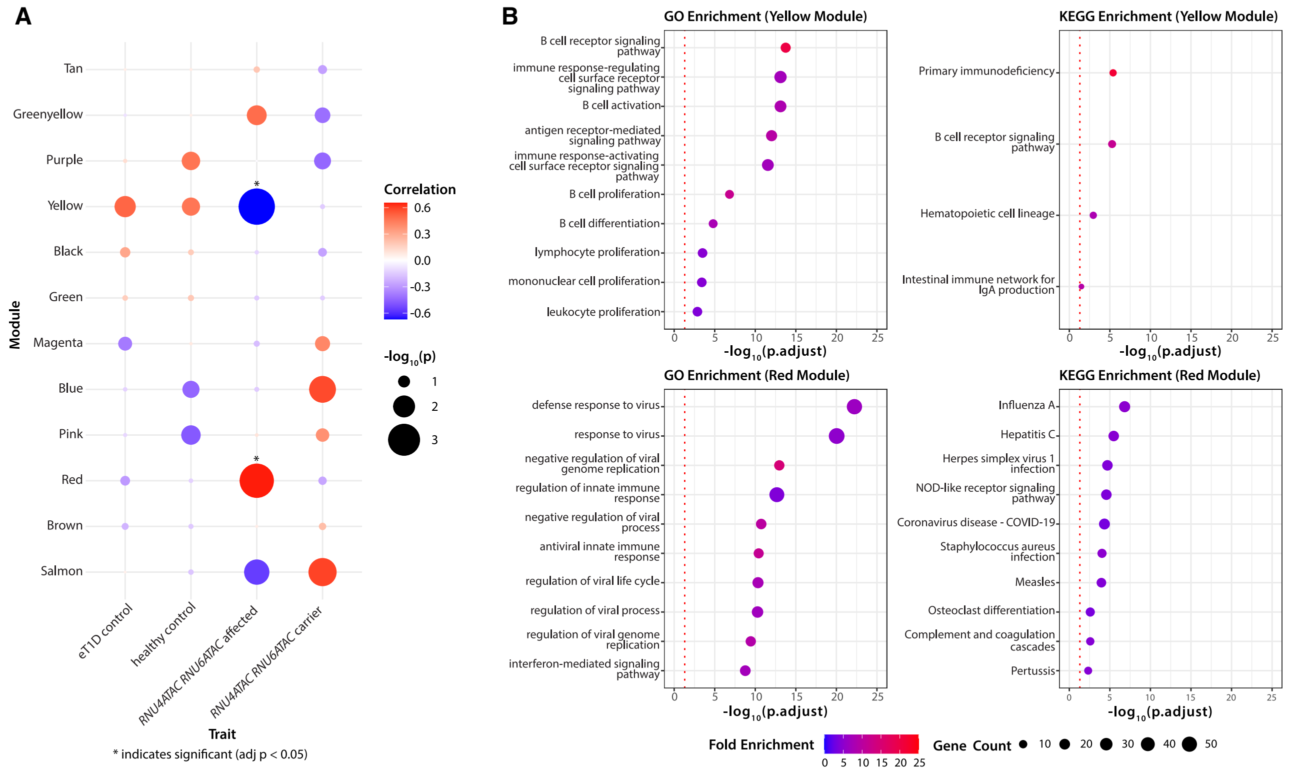

Figure 3. Weighted gene co-expression network analysis and gene enrichment analysis of resulting enriched gene modules from disease cohort RNA-seq data

(A) Correlation between weighted gene co-expression network analysis (WGCNA) and derived gene modules and disease status, which included affected individuals with *RNU6ATAC* and *RNU4ATAC* variants, carriers, age-matched T1D control subjects, and age-matched healthy control subjects. Colors are used as arbitrarily defined module names. Significant correlation after Bonferroni correction for multiple testing is shown with *.

(B) GO and KEGG enrichment analysis of significantly correlated gene modules with log-transformed adjusted p values shown. The red dotted line represents adjusted $p < 0.05$. Enriched pathways converge on immune defects, particularly B cell and other humoral immunity.

diabetes is warranted to understand the role of B cell regulation, maturation, and development in autoimmune diabetes.

The minor spliceosome is found across many eukaryote genera, though it has been lost in some.[15] While it is involved in the splicing of a small proportion of genes, many have essential functions, and minor intron splicing may play a role in temporal regulation of protein expression through slower mRNA processing.[41] This is supported by incomplete intron retention of U12 genes, as seen in the individuals analyzed in this study and previously in RNU4ATAC-opathy.[11,26] Further work is needed to untangle the role of genes undergoing minor splicing in the development of beta cell autoimmunity, as it is possible that a subset of these genes has direct pathogenic roles.

We performed methylation analysis on 17 affected individuals (7 *RNU6ATAC* and 10 *RNU4ATAC*) and RNA-seq on 6 affected individuals (3 and 3). The results of these studies supported all individuals having B cell defects, which were further validated in a single individual with bi-allelic *RNU4ATAC* variants using gold-standard immune analyses through flow cytometry. Although we would have liked to study more individuals, the geographic diversity of the cohort and the severity of disease (6 individuals deceased in early life) prevented sample collection from the remaining individuals.

In conclusion, we report bi-allelic pathogenic variants in *RNU6ATAC* as a cause of monogenic autoimmune diabetes with additional immune dysregulation and extend the phenotype of RNU4ATAC-opathies to include autoimmune diabetes. Our work provides insights into the role of these snRNAs in human immune regulation and beta cell autoimmunity and crucial new diagnoses to families.

Data and code availability

Anonymized RNA-seq data are available through application at the European Genome-phenome Archive web portal (https://ega-archive.org). The accession number for the data reported in this paper is EGA Archive: EGAS50000001565. Access to these data will be granted for appropriate use in research and will be governed by the provisions laid out in the terms contained in the data access agreement. All other non-clinical data analyzed during this study are included in this published article and supplemental information. Additional clinical, methylation array, and genotype data can be used to identify individuals

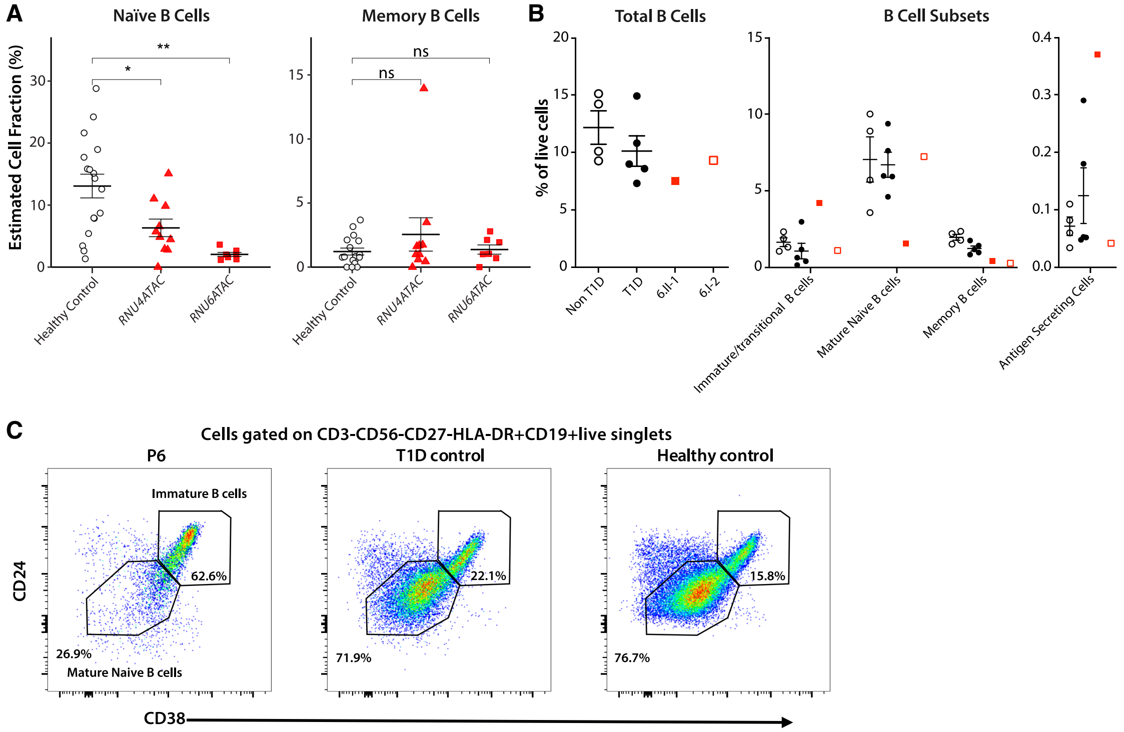

Figure 4. Immune profile analysis of affected individuals using methylation and flow cytometry
(A) Deconvolution of whole-blood-derived DNA using the IDOL library identified normal estimates of memory B cells but significantly reduced naive B cells in individuals with variants in both *RNU4ATAC* ($n = 10$) and *RNU6ATAC* ($n = 7$) compared to age-matched control subjects. Bars show mean and standard error. $*p < 0.05$, $**p < 0.01$.
(B) Flow cytometry on fresh blood cells of an *RNU4ATAC* affected individual (red triangle) identified reduced naive and increased transitional B cells, as well as elevated antigen-secreting cells (ASCs), compared to healthy control subjects (white circles) and age-matched T1D control subjects (black circles). Bars show mean and standard error.
(C) Dot plots of flow cytometry data showing markedly increased immature B cell compartment and reduced mature naive B cells in the individual with bi-allelic variants in *RNU4ATAC* (6.II-2 in Figure 1; Table 2) vs. representative control subjects.
Each plotted point for the flow cytometry data in (B) and (C) is from a single blood draw for control subjects, the affected individual, and the carrier parent.

and are therefore available through collaboration with experienced teams working on approved studies examining the mechanisms, causes, diagnosis, and treatment of diabetes and other beta cell disorders. Requests for collaboration will be considered by a steering committee following an application to the Genetic Beta Cell Research Bank (https://www.diabetesgenes.org/current-research/genetic-beta-cell-research-bank/). Contact by email should be directed to Prof. Elisa De Franco (e.de-franco@exeter.ac.uk). All requests for access to data will be responded to within 14 days. The code used in the analysis of the data is publicly available on GitHub (https://github.com/JamesR-S/RNU6ATAC_RNU4ATAC_Monogenic_Diabetes).

Consortia

The members of the EXE-T1D consortium are Rebecca A. Dobbs, Evangelina Williams, Kathleen M. Gillespie, William A. Hagopian, Amber M. Luckett, Michelle Hudson, Timothy J. McDonald, Noel G. Morgan, Kathryn Murrall, Suraj Ramchand, Sarah J. Richardson, Bart O. Roep, Bradford Dimos, Megan E. Smithmyer, and Cate Speake

The members of the ATAC clinical consortium are Elke Fink-Leinweber, Markus Lundgren, Annelie Carlsson, Ghaisani Fadiana, Frida Soesanti, Elizabeth A. Mann, M. Tracy Bekx, Tabitha Randell, Tugba Kontbay Çetin, Mahsa M. Amoli, Can Thi Bich Ngoc, Dung Chi Vu, Nguyan Hoang Lan, Saif S. Albayati, Nileema Thuse, Kalpana Jog, Chitteranjan Yajnik, Khadija N. Humayun, Patrick Willems, Adel Djermane, and Yasmine Ouarezki

Acknowledgments

We are grateful to the patients and their families for taking part in our gene discovery study. We thank Sabrina Wright and the Exeter Sequencing Facility (University of Exeter) for technical assistance and Joe Burrage and Dr. Emma Dempster for generating DNA methylation data. We are grateful to Dr. Patrick Willems and the GENDIA (Antwerp, Belgium) team and Dr. Majedah AbdulRasoul and Dr. Maria Al Mahdi (Dasman Institute, Kuwait) for patient referral and for providing clinical details. This study was supported by the National Institute for Health and Care Research Exeter Biomedical Research Centre and the National Institute for Health and Care Research Exeter Clinical Research Facility. The views expressed are those of the authors and not necessarily those of the National Institute for Health and Care Research or the Department of Health and Social

Care. M.B.J. is a Diabetes UK and Breakthrough T1D RD Lawrence Fellow (23/0006516). E.D.F. is a Diabetes UK RD Lawrence Fellow (19/005971) and the recipient of a European Foundation for the Study of Diabetes/Novo Nordisk Foundation Future Leaders Award (NNF23SA0087432). K.A.P. has a Wellcome Trust Research Fellowship (219606/Z/19/Z). S.E.F. has a Wellcome Trust Senior Research Fellowship (223187/Z/21/Z). This study was supported by The Leona M. and Harry B. Helmsley Charitable Trust (grants 2016PG-T1D049, 2018PG-T1D049, 2103–05059, and G-2404-06858) and a Wellcome Trust Collaborative Award in Science to E.D.F. and A.T.H. (grant no. 224600/Z/21/Z). For the purpose of open access, the author has applied a CC BY public copyright license to any author-accepted manuscript version arising from this submission.

Author contributions

M.B.J., J.R.-S., A.T.H., and E.D.F. designed the study, analyzed and interpreted the data, wrote the manuscript, and directed the project. P.A.B., C.D.-V., and T.I.M.T. performed the immunological experiments, interpreted the resulting data, and wrote the manuscript. M.G. and G.B. performed experiments and interpreted the resulting data. M.N.W. and J.R.-S. wrote the scripts to analyze genome sequencing data. M.B.J., S.E.F., K.A.P., E.D.F., A.T.H., and the ATAC clinical consortium recruited patients and interpreted the clinical data. M.J. and E.D.F. interpreted the sequence variants. R.A.O. interpreted the clinical data and recruited patients for flow. The EXE-T1D consortium contributed to the recruitment of individuals for immune studies. All authors contributed to drafting the final manuscript. E.D.F. is the guarantor of the study and data.

Declaration of interests

The authors declare no competing interests.

Web resources

European Genome-phenome Archive, https://ega-archive.org
GenBank, https://www.ncbi.nlm.nih.gov/genbank/
OMIM, https://www.omim.org

Supplemental information

Supplemental information can be found online at https://doi.org/10.1016/j.ajhg.2026.02.017.

Received: December 1, 2025
Accepted: February 25, 2026
Published: March 20, 2026

References

1. Turro, E., Astle, W.J., Megy, K., Gräf, S., Greene, D., Shamardina, O., Allen, H.L., Sanchis-Juan, A., Frontini, M., Thys, C., et al. (2020). Whole-genome sequencing of patients with rare diseases in a national health system. Nature *583*, 96–102. https://doi.org/10.1038/s41586-020-2434-2.
2. Graessner, H., Zurek, B., Hoischen, A., and Beltran, S. (2021). Solving the unsolved rare diseases in Europe. Eur. J. Hum. Genet. *29*, 1319–1320. https://doi.org/10.1038/s41431-021-00924-8.
3. Wakeling, M.N., Owens, N.D.L., Hopkinson, J.R., Johnson, M.B., Houghton, J.A.L., Dastamani, A., Flaxman, C.S., Wyatt, R.C., Hewat, T.I., Hopkins, J.J., et al. (2022). Non-coding variants disrupting a tissue-specific regulatory element in HK1 cause congenital hyperinsulinism. Nat. Genet. *54*, 1615–1620. https://doi.org/10.1038/s41588-022-01204-x.
4. Weedon, M.N., Cebola, I., Patch, A.-M., Flanagan, S.E., De Franco, E., Caswell, R., Rodríguez-Seguí, S.A., Shaw-Smith, C., Cho, C.H.-H., Allen, H.L., et al. (2014). Recessive mutations in a distal PTF1A enhancer cause isolated pancreatic agenesis. Nat. Genet. *46*, 61–64. https://doi.org/10.1038/ng.2826.
5. Whiffin, N., Karczewski, K.J., Zhang, X., Chothani, S., Smith, M.J., Evans, D.G., Roberts, A.M., Quaife, N.M., Schafer, S., Rackham, O., et al. (2020). Characterising the loss-of-function impact of 5′ untranslated region variants in 15,708 individuals. Nat. Commun. *11*, 2523. https://doi.org/10.1038/s41467-019-10717-9.
6. Ganesh, V.S., Riquin, K., Chatron, N., Yoon, E., Lamar, K.-M., Aziz, M.C., Monin, P., O'Leary, M.C., Goodrich, J.K., Garimella, K.V., et al. (2024). Neurodevelopmental Disorder Caused by Deletion of CHASERR, a lncRNA Gene. N. Engl. J. Med. *391*, 1511–1518. https://doi.org/10.1056/NEJMoa2400718.
7. Chen, Y., Dawes, R., Kim, H.C., Ljungdahl, A., Stenton, S.L., Walker, S., Lord, J., Lemire, G., Martin-Geary, A.C., Ganesh, V.S., et al. (2024). De novo variants in the RNU4-2 snRNA cause a frequent neurodevelopmental syndrome. Nature *632*, 832–840. https://doi.org/10.1038/s41586-024-07773-7.
8. Greene, D., Thys, C., Berry, I.R., Jarvis, J., Ortibus, E., Mumford, A.D., Freson, K., and Turro, E. (2024). Mutations in the U4 snRNA gene RNU4-2 cause one of the most prevalent monogenic neurodevelopmental disorders. Nat. Med. *30*, 2165–2169. https://doi.org/10.1038/s41591-024-03085-5.
9. Chen, S., Francioli, L.C., Goodrich, J.K., Collins, R.L., Kanai, M., Wang, Q., Alföldi, J., Watts, N.A., Vittal, C., Gauthier, L.D., et al. (2024). A genomic mutational constraint map using variation in 76,156 human genomes. Nature *625*, 92–100. https://doi.org/10.1038/s41586-023-06045-0.
10. Landrum, M.J., Lee, J.M., Riley, G.R., Jang, W., Rubinstein, W.S., Church, D.M., and Maglott, D.R. (2014). ClinVar: public archive of relationships among sequence variation and human phenotype. Nucleic Acids Res. *42*, D980–D985. https://doi.org/10.1093/nar/gkt1113.
11. Almentina Ramos Shidi, F., Cologne, A., Delous, M., Besson, A., Putoux, A., Leutenegger, A.-L., Lacroix, V., Edery, P., Mazoyer, S., and Bordonné, R. (2023). Mutations in the non-coding RNU4ATAC gene affect the homeostasis and function of the Integrator complex. Nucleic Acids Res. *51*, 712–727. https://doi.org/10.1093/nar/gkac1182.
12. Ellard, S., Lango Allen, H., De Franco, E., Flanagan, S.E., Hysenaj, G., Colclough, K., Houghton, J.A.L., Shepherd, M., Hattersley, A.T., Weedon, M.N., and Caswell, R. (2013). Improved genetic testing for monogenic diabetes using targeted next-generation sequencing. Diabetologia *56*, 1958–1963. https://doi.org/10.1007/s00125-013-2962-5.
13. Singh, J., Sikand, K., Conrad, H., Will, C.L., Komar, A.A., and Shukla, G.C. (2016). U6atac snRNA stem-loop interacts

with U12 p65 RNA binding protein and is functionally interchangeable with the U12 apical stem-loop III. Sci. Rep. *6*, 31393. https://doi.org/10.1038/srep31393.
14. Bai, R., Yuan, M., Zhang, P., Luo, T., Shi, Y., and Wan, R. (2024). Structural basis of U12-type intron engagement by the fully assembled human minor spliceosome. Science *383*, 1245–1252. https://doi.org/10.1126/science.adn7272.
15. Turunen, J.J., Niemelä, E.H., Verma, B., and Frilander, M.J. (2013). The significant other: splicing by the minor spliceosome. Wiley Interdiscip. Rev. RNA *4*, 61–76. https://doi.org/10.1002/wrna.1141.
16. Verma, B., Akinyi, M.V., Norppa, A.J., and Frilander, M.J. (2018). Minor spliceosome and disease. Semin. Cell Dev. Biol. *79*, 103–112. https://doi.org/10.1016/j.semcdb.2017.09.036.
17. Bai, R., Wan, R., Wang, L., Xu, K., Zhang, Q., Lei, J., and Shi, Y. (2021). Structure of the activated human minor spliceosome. Science *371*, eabg0879. https://doi.org/10.1126/science.abg0879.
18. Duker, A., Velasco, D., Robertson, N., Jackson, A., DeFelice, M., and Bober, M.B. (1993). RNU4atac-opathy. In GeneReviews®, M.P. Adam, J. Feldman, G.M. Mirzaa, R.A. Pagon, S.E. Wallace, and A. Amemiya, eds. (University of Washington).
19. Zhang, J., Merialdi, M., Platt, L.D., and Kramer, M.S. (2010). Defining normal and abnormal fetal growth: promises and challenges. Am. J. Obstet. Gynecol. *202*, 522–528. https://doi.org/10.1016/j.ajog.2009.10.889.
20. Dinur Schejter, Y., Ovadia, A., Alexandrova, R., Thiruvahindrapuram, B., Pereira, S.L., Manson, D.E., Vincent, A., Merico, D., and Roifman, C.M. (2017). A homozygous mutation in the stem II domain of RNU4ATAC causes typical Roifman syndrome. npj Genom. Med. *2*, 23. https://doi.org/10.1038/s41525-017-0024-5.
21. Xi, Q., Plaza Enriquez, L.J., Tanni, N.U., and Patsias, I. (2023). Underdiagnosed Roifman syndrome manifested as non-ischaemic cardiomyopathy: a case report. ESC Heart Fail. *10*, 3195–3198. https://doi.org/10.1002/ehf2.14518.
22. Arriaga, M.T., Mendez, R., Ungar, R.A., Bonner, D.E., Matalon, D.R., Lemire, G., Goddard, P.C., Padhi, E.M., Miller, A.M., Nguyen, J.V., et al. (2025). Transcriptome-wide outlier approach identifies individuals with minor spliceopathies. Am. J. Hum. Genet. *112*, 2458–2475. https://doi.org/10.1016/j.ajhg.2025.08.018.
23. Johnson, M.B., Patel, K.A., De Franco, E., Houghton, J.A.L., McDonald, T.J., Ellard, S., Flanagan, S.E., and Hattersley, A.T. (2018). A type 1 diabetes genetic risk score can discriminate monogenic autoimmunity with diabetes from early-onset clustering of polygenic autoimmunity with diabetes. Diabetologia *61*, 862–869. https://doi.org/10.1007/s00125-018-4551-0.
24. Johnson, M.B., Patel, K.A., De Franco, E., Hagopian, W., Killian, M., McDonald, T.J., Tree, T.I.M., Domingo-Vila, C., Hudson, M., Hammersley, S., et al. (2020). Type 1 diabetes can present before the age of 6 months and is characterised by autoimmunity and rapid loss of beta cells. Diabetologia *63*, 2605–2615. https://doi.org/10.1007/s00125-020-05276-4.
25. Moyer, D.C., Larue, G.E., Hershberger, C.E., Roy, S.W., and Padgett, R.A. (2020). Comprehensive database and evolutionary dynamics of U12-type introns. Nucleic Acids Res. *48*, 7066–7078. https://doi.org/10.1093/nar/gkaa464.
26. Merico, D., Roifman, M., Braunschweig, U., Yuen, R.K.C., Alexandrova, R., Bates, A., Reid, B., Nalpathamkalam, T., Wang, Z., Thiruvahindrapuram, B., et al. (2015). Compound heterozygous mutations in the noncoding RNU4ATAC cause Roifman Syndrome by disrupting minor intron splicing. Nat. Commun. *6*, 8718. https://doi.org/10.1038/ncomms9718.
27. Khatri, D., Putoux, A., Cologne, A., Kaltenbach, S., Besson, A., Bertiaux, E., Guguin, J., Fendler, A., Dupont, M.A., Benoit-Pilven, C., et al. (2023). Deficiency of the minor spliceosome component U4atac snRNA secondarily results in ciliary defects in human and zebrafish. Proc. Natl. Acad. Sci. USA *120*, e2102569120. https://doi.org/10.1073/pnas.2102569120.
28. Zhang, B., and Horvath, S. (2005). A general framework for weighted gene co-expression network analysis. Stat. Appl. Genet. Mol. Biol. *4*, Article17. https://doi.org/10.2202/1544-6115.1128.
29. Ashburner, M., Ball, C.A., Blake, J.A., Botstein, D., Butler, H., Cherry, J.M., Davis, A.P., Dolinski, K., Dwight, S.S., Eppig, J.T., et al. (2000). Gene Ontology: tool for the unification of biology. Nat. Genet. *25*, 25–29. https://doi.org/10.1038/75556.
30. Gene Ontology Consortium, Aleksander, S.A., Balhoff, J., Carbon, S., Cherry, J.M., Drabkin, H.J., Ebert, D., Feuermann, M., Gaudet, P., Harris, N.L., and et al.. (2023). The Gene Ontology knowledgebase in 2023. Genetics *224*, iyad031. https://doi.org/10.1093/genetics/iyad031.
31. Kanehisa, M., and Goto, S. (2000). KEGG: kyoto encyclopedia of genes and genomes. Nucleic Acids Res. *28*, 27–30. https://doi.org/10.1093/nar/28.1.27.
32. Salas, L.A., Zhang, Z., Koestler, D.C., Butler, R.A., Hansen, H.M., Molinaro, A.M., Wiencke, J.K., Kelsey, K.T., and Christensen, B.C. (2022). Enhanced cell deconvolution of peripheral blood using DNA methylation for high-resolution immune profiling. Nat. Commun. *13*, 761. https://doi.org/10.1038/s41467-021-27864-7.
33. Heremans, J., Garcia-Perez, J.E., Turro, E., Schlenner, S.M., Casteels, I., Collin, R., de Zegher, F., Greene, D., Humblet-Baron, S., Lesage, S., et al. (2018). Abnormal differentiation of B cells and megakaryocytes in patients with Roifman syndrome. J. Allergy Clin. Immunol. *142*, 630–646. https://doi.org/10.1016/j.jaci.2017.11.061.
34. Robertson, N., Joshi, A., Ritchie, F., Schim van der Loeff, I., Royan, D., Duker, A.L., Rice, G.I., Bober, M.B., Mansour, S., Campbell, D.I., et al. (2025). Mutations in RNU4ATAC Are Associated With Chilblain-Like Lesions and Enhanced Type I Interferon Signalling. Eur. J. Immunol. *55*, e202451518. https://doi.org/10.1002/eji.202451518.
35. Gauthier, L.W., Gossez, M., Malcus, C., Viel, S., Monneret, G., Bordonné, R., Pons, L., Cabet, S., Delous, M., Mazoyer, S., et al. (2024). B-cell immune deficiency in twin sisters expands the phenotype of MOPDI. Clin. Genet. *106*, 476–482. https://doi.org/10.1111/cge.14571.
36. Ciavarella, J., Perea, W., and Greenbaum, N.L. (2020). Topology of the U12-U6(atac) snRNA Complex of the Minor Spliceosome and Binding by NTC-Related Protein RBM22. ACS Omega *5*, 23549–23558. https://doi.org/10.1021/acsomega.0c01674.
37. Bloem, S.J., and Roep, B.O. (2017). The elusive role of B lymphocytes and islet autoantibodies in (human) type 1

diabetes. Diabetologia *60*, 1185–1189. https://doi.org/10.1007/s00125-017-4284-5.

38. Arif, S., Leete, P., Nguyen, V., Marks, K., Nor, N.M., Estorninho, M., Kronenberg-Versteeg, D., Bingley, P.J., Todd, J.A., Guy, C., et al. (2014). Blood and islet phenotypes indicate immunological heterogeneity in type 1 diabetes. Diabetes *63*, 3835–3845. https://doi.org/10.2337/db14-0365.
39. Pescovitz, M.D., Greenbaum, C.J., Krause-Steinrauf, H., Becker, D.J., Gitelman, S.E., Goland, R., Gottlieb, P.A., Marks, J.B., McGee, P.F., Moran, A.M., et al. (2009). Rituximab, B-lymphocyte depletion, and preservation of beta-cell function. N. Engl. J. Med. *361*, 2143–2152. https://doi.org/10.1056/NEJMoa0904452.
40. Martin, S., Wolf-Eichbaum, D., Duinkerken, G., Scherbaum, W.A., Kolb, H., Noordzij, J.G., and Roep, B.O. (2001). Development of type 1 diabetes despite severe hereditary B-cell deficiency. N. Engl. J. Med. *345*, 1036–1040. https://doi.org/10.1056/NEJMoa010465.
41. Patel, A.A., McCarthy, M., and Steitz, J.A. (2002). The splicing of U12-type introns can be a rate-limiting step in gene expression. EMBO J. *21*, 3804–3815. https://doi.org/10.1093/emboj/cdf297.

The American Journal of Human Genetics, Volume *113*

Supplemental information

Bi-allelic variants in the non-protein-coding minor spliceosome components *RNU6ATAC* and *RNU4ATAC* cause syndromic monogenic autoimmune diabetes

Matthew B. Johnson, James Russ-Silsby, Paul A. Blair, Molly Govier, Georgia Bonfield, Clara Domingo-Vila, EXE-T1D consortium, ATAC clinical consortium, Matthew N. Wakeling, Richard A. Oram, Sarah E. Flanagan, Timothy I.M. Tree, Kashyap A. Patel, Andrew T. Hattersley, and Elisa De Franco

1. EXE-T1D CONSORTIUM

R A Dobbs[1], E Williams[2], K M Gillespie[3], W A Hagopian[4,5], A M Luckett[1], M Hudson[1], T J McDonald[1], N G Morgan[1], K Murrall[1], S Ramchand[1], S J Richardson[1], B O Roep[6], B Dimos[7], M E Smithmyer[7], C Speake[7]

1. Department of Clinical & Biomedical Sciences, University of Exeter Medical School, Exeter, UK
2. Department of Immunobiology, School of Immunology & Microbial Sciences (SIMS), King's College, London, UK
3. Translational Health Sciences, Bristol Medical School, University of Bristol, Southmead Hospital, Bristol, UK
4. Department of Paediatrics, Indiana University School of Medicine, Indianapolis, IN, USA.
5. Department of Medicine, University of Washington, Seattle, WA, USA.
6. Department of Internal Medicine, Leids Universitair Medisch Centrum, Leiden, Netherlands
7. Center for Interventional Immunology, Benaroya Research Institute, Seattle, WA

2. ATAC CLINICAL CONSORTIUM

E Fink-Leinweber[1], M Lundgren[2,3], A Carlsson[4], G Fadiana[5], F Soesanti[5], E A Mann[6], M T Bekx[7], T Randell[8], T Kontbay Çetin[9], M M Amoli[10], Can Thi Bich Ngoc[11], D C Vu[11], N H Lan[11], S S Albayati [12], N Thuse[13], K Jog[13], C Yajnik[13], K N Humayun[14], P Willems[15], A Djermane[16, 17], Y Ouarezki[16, 17]

1 - Märkische Kliniken GmbH Klinikum Lüdenscheid, Lüdenscheid, Germany
2 - Department of clinical sciences Malmö, Lund university, Sweden
3 - Skåne university Hospital, Kristianstad, Sweden
4 - Department of Pediatrics, Skånes University Hospital, Lund University, Sweden
5 - Child Health Department, Faculty of Medicine, Universitas Indonesia, Cipto Mangunkusumo General Hospital, Jakarta, Indonesia
6- Division of Endocrinology and Diabetes, Department of Pediatrics, University Hospital, Madison, US
7 - American Family Children's Hospital, University of Wisconsin-Madison, Madison Wisconsin
8 - Nottingham University Hospitals NHS Trust, Nottingham, UK
9 - Sanliurfa research and training hospital, Sanliurfa, Turkey
10 - Metabolic Disorders Research Centre, Endocrinology and Metabolism Molecular-Cellular Sciences Institute, Tehran University of Medical Sciences, Tehran, Iran.
11 - Center for Endocrinology, Metabolism, Genetics/Genomics and Molecular Therapy, National Children's Hospital, Hanoi, Vietnam
12 - Child central teaching hospital, Baghdad, Iraq
13 - Diabetes Unit, KEM Hospital and Research Centre, Pune, India
14 - Aga Khan University, Karachi, Pakistan
15 - Gendia, Antwerp, Belgium
16 - EPH Hassan Badi, El-Harrach, Algiers, Algeria
17 - Université de Sciences de la Santé, Faculté de médecine d'Alger, Algeria

2. Supplementary Figures

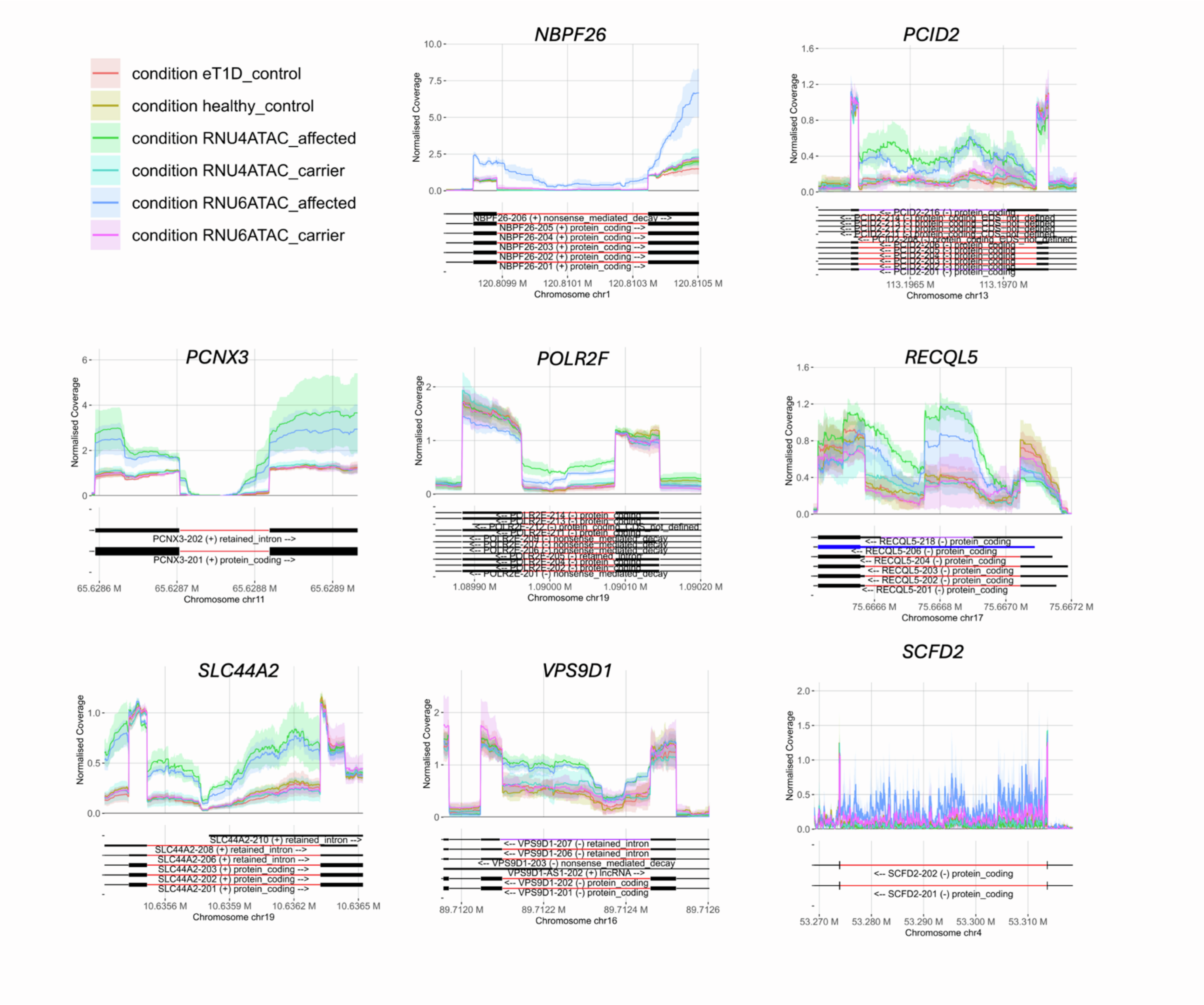

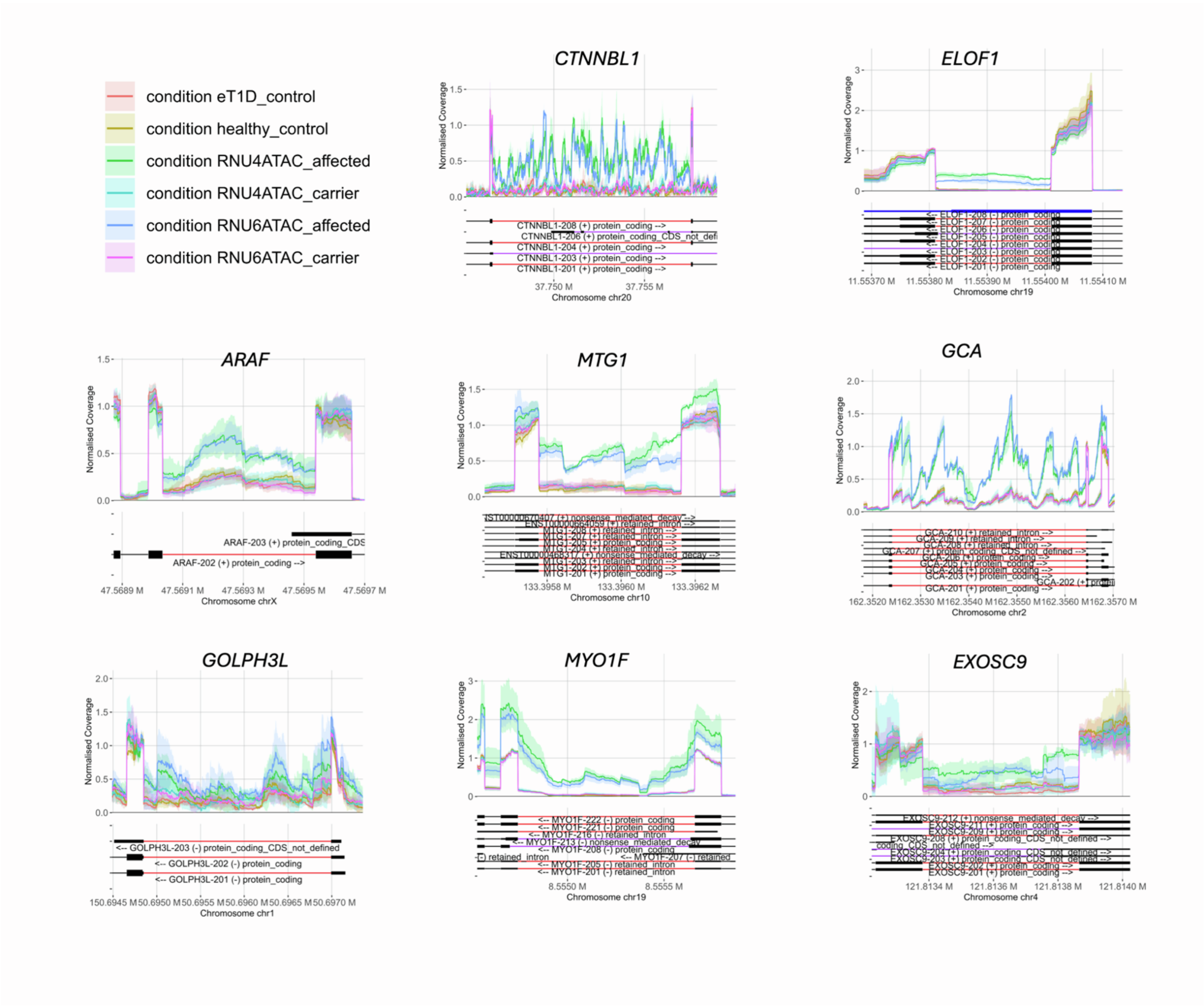

Figure S1: Coverage plots showing putative novel U12 genes. Genes with significant intron retention in whole blood RNA from individuals with biallelic pathogenic variants in *RNU4ATAC*, *RNU6ATAC* or both (table S3) but not present in the IAOD database are shown (https://introndb.lerner.ccf.org/)[3].

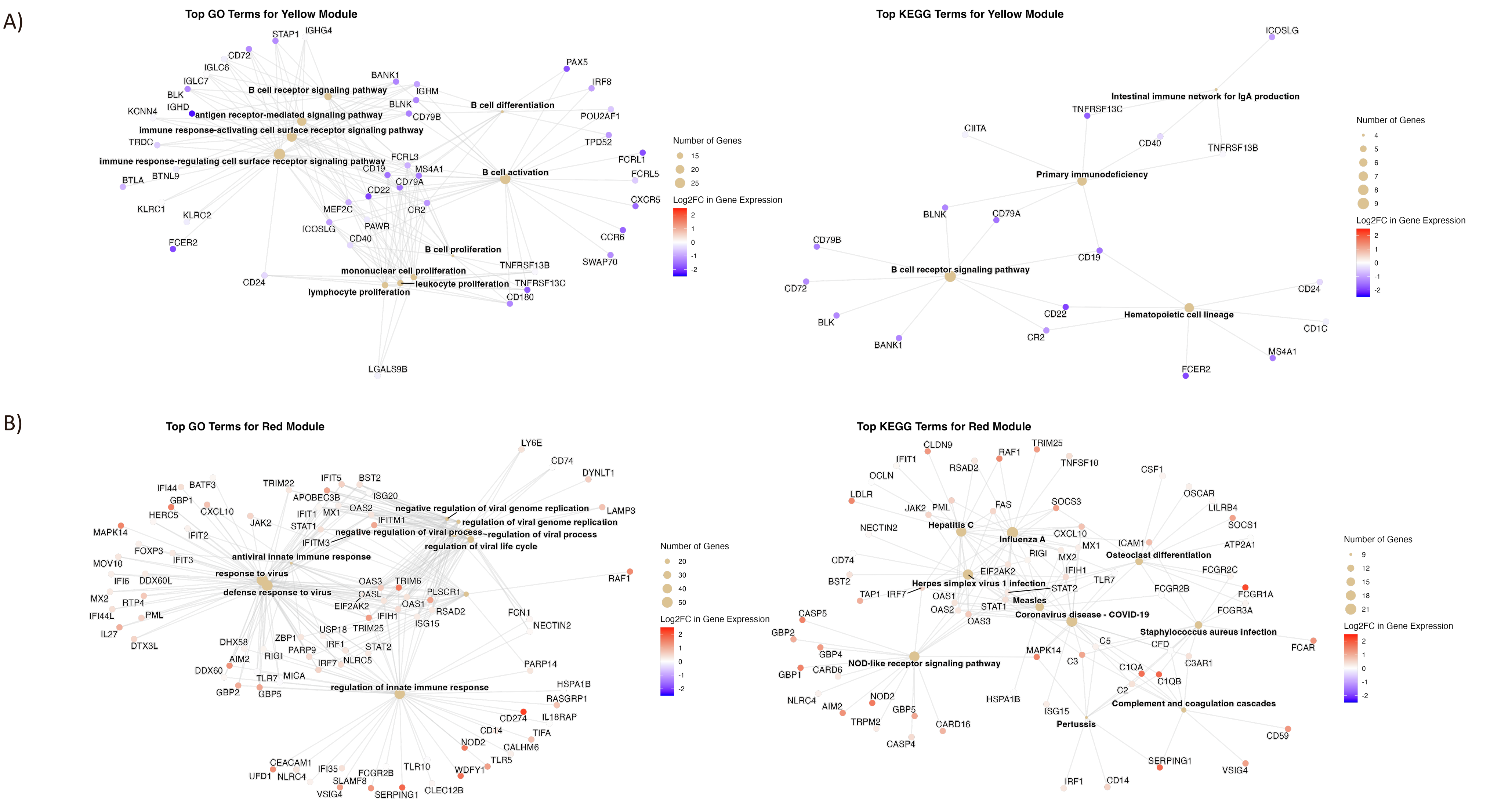

Figure S2: Enrichment analysis of WGCNA gene modules. For the two significant modules, GO and KEGG enrichment was used to identify pathways connected to the gene lists.

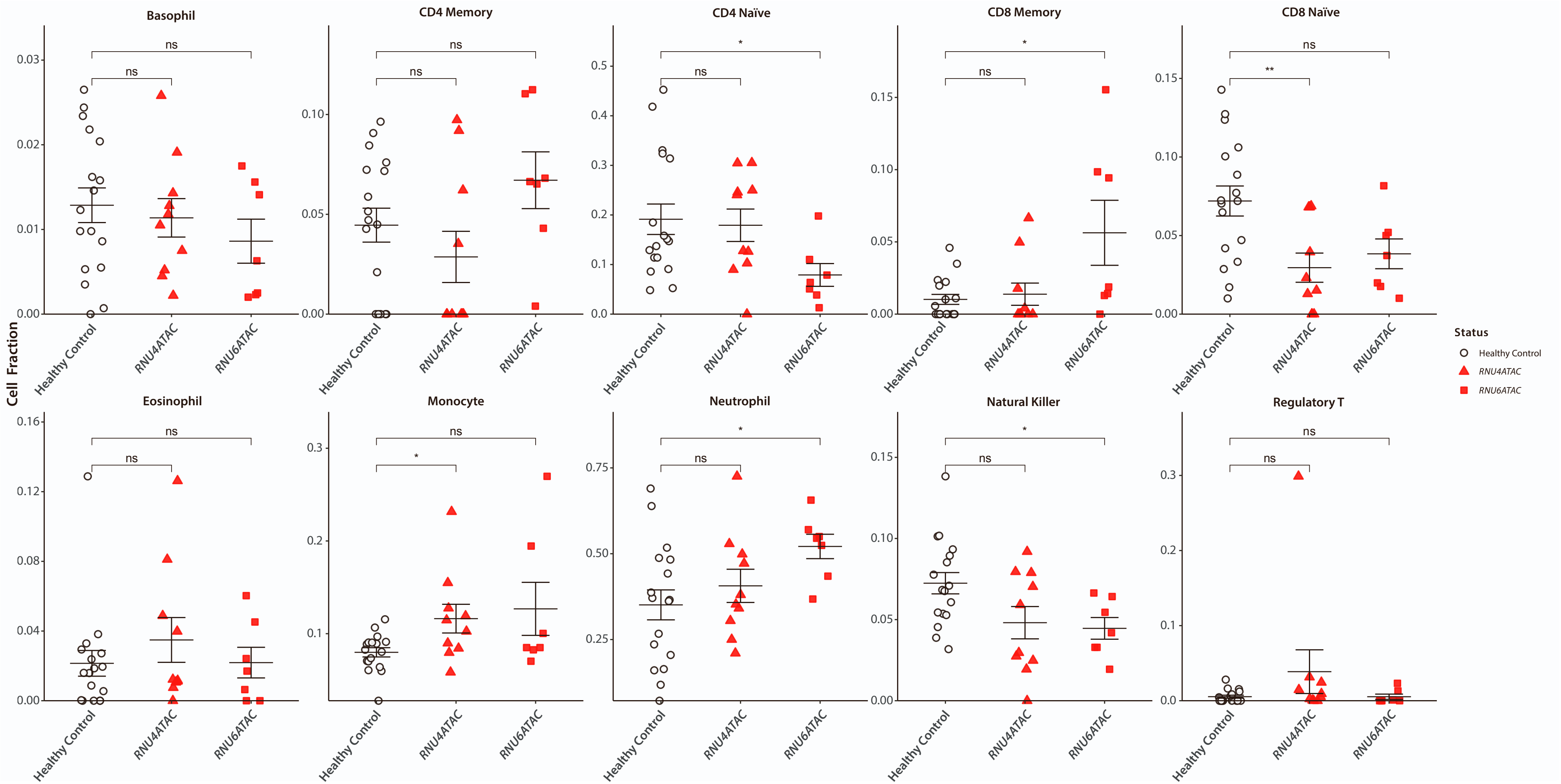

Figure S3: Estimates of immune cell proportions from deconvolution of EPIC array methylation analysis of whole blood-derived DNA.

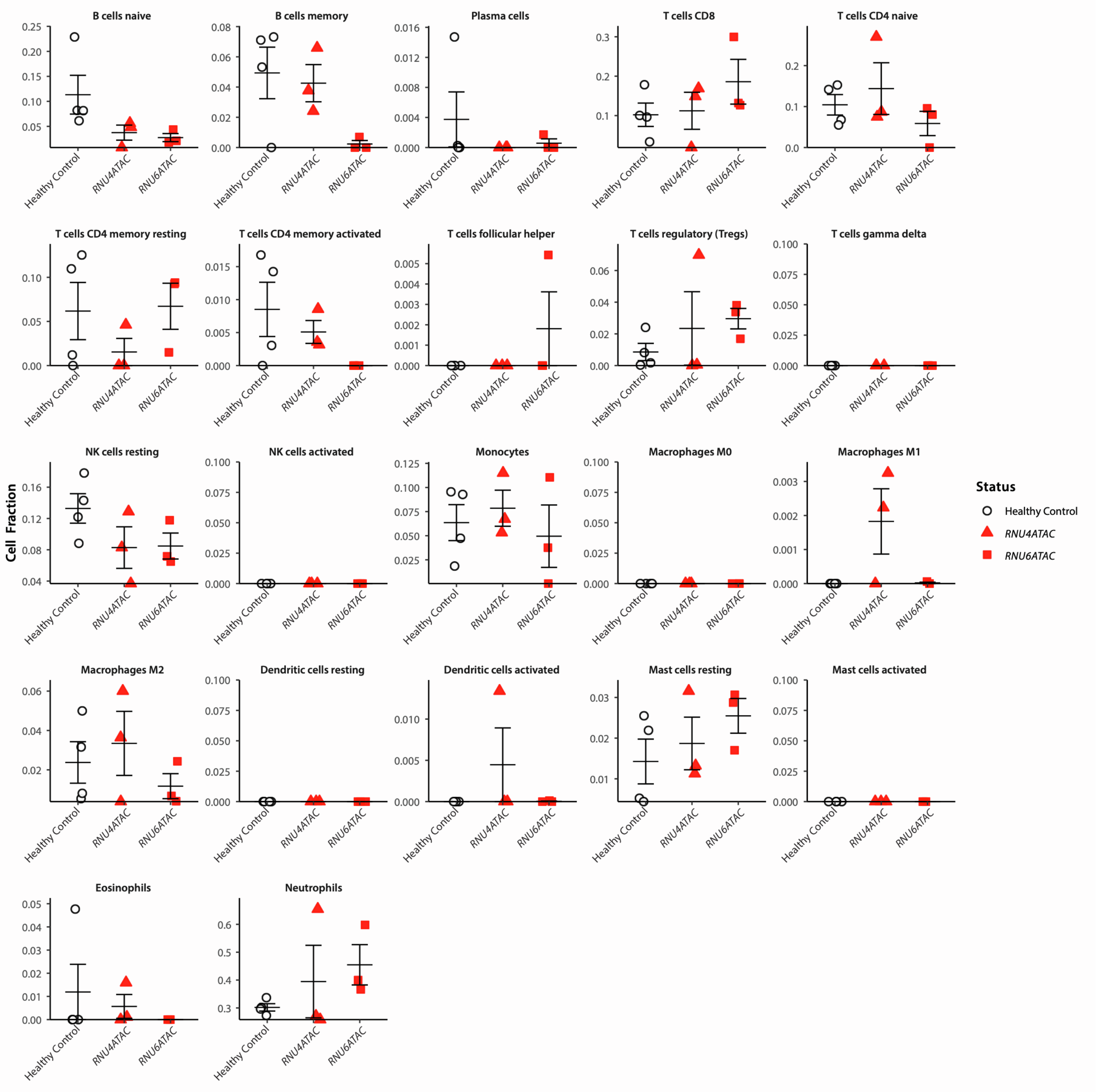

Figure S4: Estimates of immune cell proportions in individuals with biallelic variants in *RNU4ATAC* and *RNU6AAC* and healthy controls derived from deconvolution of whole blood RNA-Sequencing data deconvolution performed using CibersortX[4].

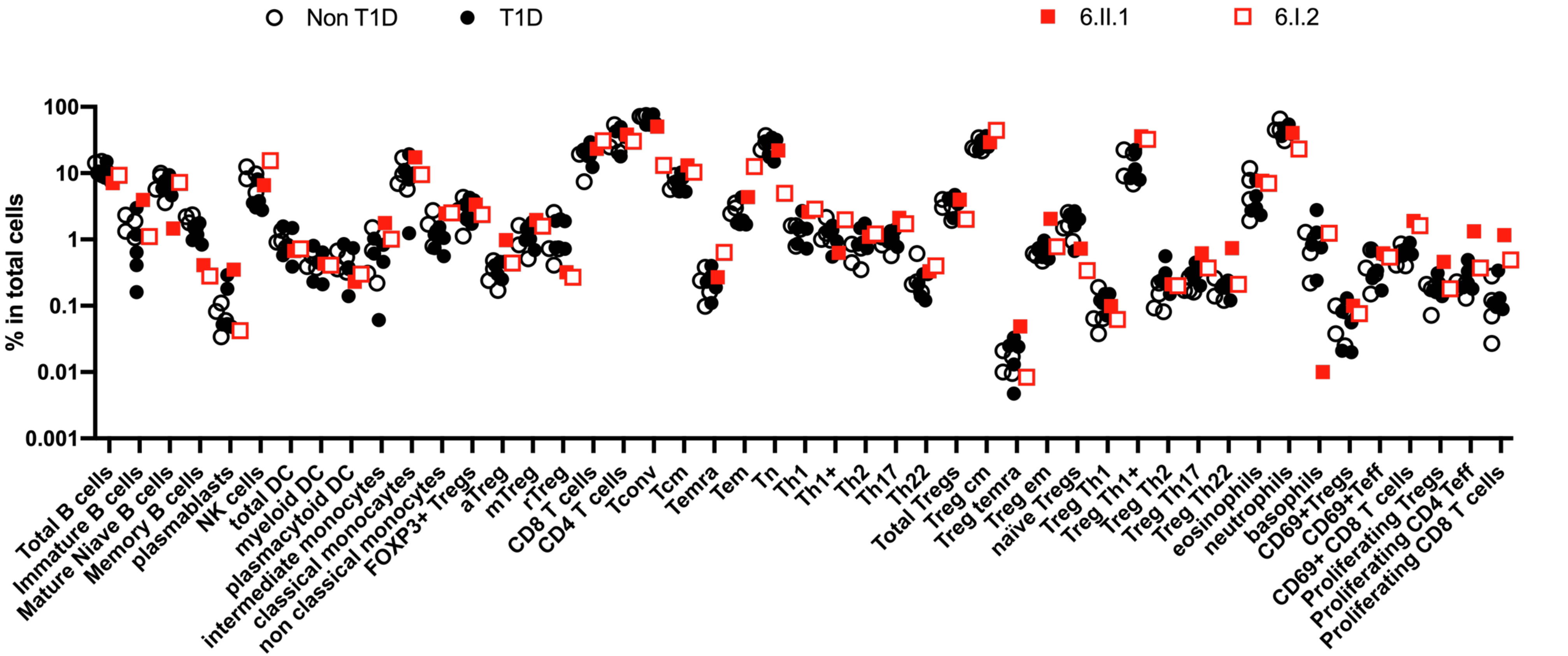

Figure S5: **Immune phenotyping of individual with biallelic *RNU4ATAC* variants, their unaffected heterozygous carrier mother, and age matched controls**.
6.I.2 – mother of 6.II.1.

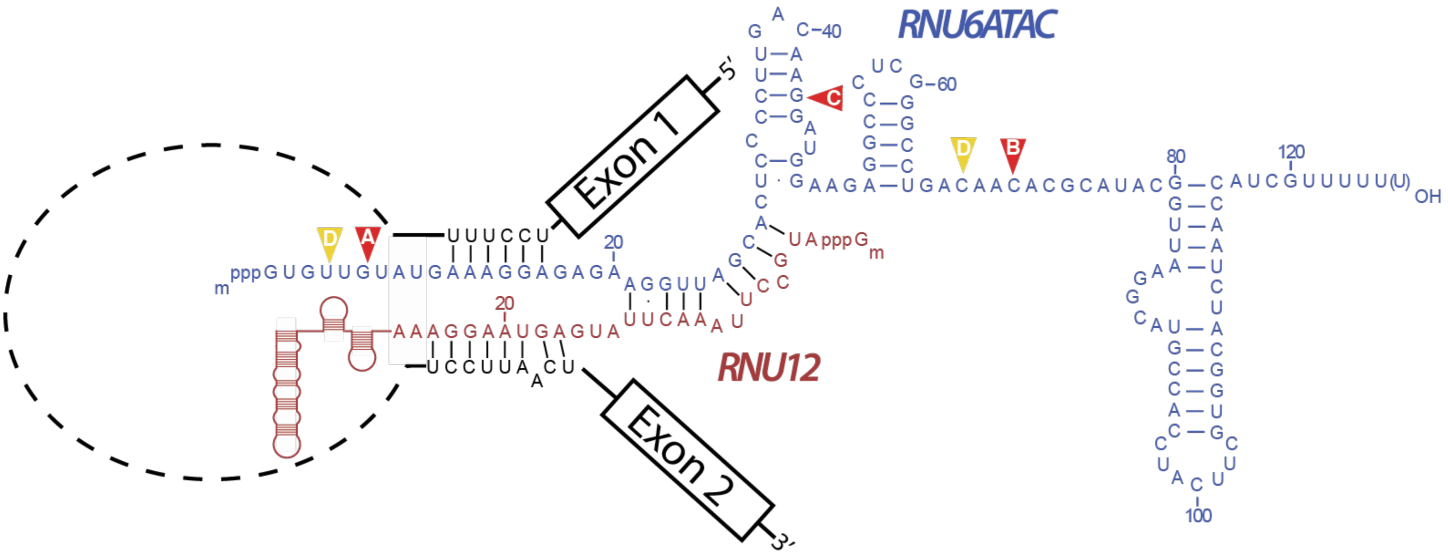

Figure S6 Positions of *RNU6ATAC* variants within the RNU6ATAC-RNU12-U12 intron complex. Dashed line shows U12 intron. Red triangles show homozygous variants, yellow triangles show compound heterozygous variants.

3. SUPPLEMENTARY TABLES

Protein	Subcomplex	Gene	Chr	Start	End	OMIM Inheritance	OMIM disease(s)
PRP8	U5 snRNP	*PRPF8*	17	1553923	1588176	Autosomal Dominant	RETINITIS PIGMENTOSA 13
SNU114	U5 snRNP	*EFTUD2*	17	42927311	42977030	Autosomal Dominant	MANDIBULOFACIAL DYSOSTOSIS, GUION-ALMEIDA TYPE
BRR2	U5 snRNP	*SNRNP200*	2	96940074	96971297	Autosomal Dominant	RETINITIS PIGMENTOSA 33
U5-40K	U5 snRNP	*SNRNP40*	1	31732417	31769662	-	None
PRP6	U5 snRNP	*PRPF6*	20	62612488	62664453	Autosomal Dominant	RETINITIS PIGMENTOSA 60
DIM2	U5 snRNP	*TXNL4B*	16	72078188	72128330	-	None
PRP28	U5 snRNP	*DDX23*	12	49223547	49246625	-	None
SmD3	U5 snRNP,U11 snRNP, U12 snRNP & U4atac/U6atac di-snRNP	*SNRPD3*	22	24951471	25005947	-	None
SmB	U5 snRNP,U11 snRNP, U12 snRNP & U4atac/U6atac di-snRNP	*SNRPB*	20	2442280	2451499	Autosomal Dominant	CEREBROCOSTOMANDIBULAR SYNDROME
SmD1	U5 snRNP,U11 snRNP, U12 snRNP & U4atac/U6atac di-snRNP	*SNRPD1*	18	19192228	19210417	-	None
SmD2	U5 snRNP,U11 snRNP, U12 snRNP & U4atac/U6atac di-snRNP	*SNRPD2*	19	46190712	46195827	-	None
SmF	U5 snRNP,U11 snRNP, U12 snRNP & U4atac/U6atac di-snRNP	*SNRPF*	12	96252706	96297606	-	None
SmE	U5 snRNP,U11 snRNP, U12 snRNP & U4atac/U6atac di-snRNP	*SNRPE*	1	203830731	203839678	Autosomal Dominant	Hypotrichosis 11 and Microcephaly(pending confirmation)
SmG	U5 snRNP,U11 snRNP, U12 snRNP & U4atac/U6atac di-snRNP	*SNRPG*	2	70508494	70520903	-	None
SCNM1	U12 snRNP	*SCNM1*	1	151129140	151142773	Autosomal Recessive	OROFACIODIGITAL SYNDROME XIX
SF3b155	U12 snRNP	*SF3B1*	2	198254508	198299815	Somatic	myelodysplastic syndrome(somatic mutations)
SF3b145	U12 snRNP	*SF3B2*	11	65818200	65836779	Autosomal Dominant	CRANIOFACIAL MICROSOMIA 1
SF3b130	U12 snRNP	*SF3B3*	16	70557691	70608820	-	None
SF3b49	U12 snRNP	*SF3B4*	1	149895209	149900236	Autosomal Dominant	ACROFACIAL DYSOSTOSIS 1, -GER TYPE
SF3b14a	U12 snRNP	*SF3B6*	2	24290454	24299313	-	None
SF3b14b	U12 snRNP	*PHF5A*	22	41855721	41864729	-	None
SF3b10	U12 snRNP	*SF3B5*	6	144416018	144416754	-	None
U11/U12-31K	U12 snRNP	*ZCRB1*	12	42705880	42719920	-	None
U11/U12-65K	U12 snRNP	*RNPC3*	1	104068313	104097861	Autosomal Recessive	PITUITARY HORMONE DEFICIENCY, COMBINED, 7
ZRSR2	U12 snRNP	*ZRSR2*	X	15808595	15841383	-	None
CDC5L	nineteen complex (NTC)	*CDC5L*	6	44355262	44418163	-	None
SYF3	nineteen complex (NTC)	*CRNKL1*	20	20015012	20036690	-	None
CWC15	NTC-related (NTR)	*CWC15*	11	94695787	94706776	-	No Entry
SKIP	NTC-related (NTR)	*SNW1*	14	78183942	78227550	-	None
PLRG1	NTC-related (NTR)	*PLRG1*	4	155456158	155471587	-	None
SNIP1	retention and splicing (RES) complex	*SNIP1*	1	38000050	38019903	Autosomal Recessive	NEURODEVELOPMENTAL DISORDER WITH HYPOTONIA, CRANIOFACIAL ABNORMALITIES, AND SEIZURES
RBMX2	retention and splicing (RES) complex	*RBMX2*	X	129535943	129547317	-	No entry
BUD13	retention and splicing (RES) complex	*BUD13*	11	116618886	116643704	Autosomal Recessive	Achalasia-progeroid syndrome
PPIL2	prolyl peptidyl isomerase (PPIase)–like proteins	*PPIL2*	22	22006559	22054304	-	None
CWC27	prolyl peptidyl isomerase (PPIase)–like proteins	*CWC27*	5	64064757	64314590	Autosomal Recessive	RETINITIS PIGMENTOSA WITH OR WITHOUT SKELETAL ANOMALIES
PRP2	Splicing Factors	*DHX16*	6	30620896	30640814	Autosomal Dominant	NEUROMUSCULAR OCULOAUDITORY SYNDROME
GPKOW	Splicing Factors	*GPKOW*	X	48970334	48980151	X-Linked Recessive	X-linked microhydranencephaly (unconfuirmed)
RNF113A	Splicing Factors	*RNF113A*	X	119004497	119005791	X-Linked Recessive	TRICHOTHIODYSTROPHY 5, NONPHOTOSENSITIVE
SRm300	Splicing Factors	*SRRM2*	16	2802330	2822539	Autosomal Dominant	INTELLECTUAL DEVELOPMENTAL DISORDER, AUTOSOMAL DOMINANT 72

CWC22	Splicing Factors	*CWC22*	2	18080960 3	180871840	-	None
SRm160	Splicing Factors	*SRRM1*	1	24958207	24999758	-	None
CRIPT	Splicing Factors	*CRIPT*	2	46843555	46852881	Autosomal Recessive	ROTHMUND-THOMSON SYNDROME, TYPE 3
RBM48	Splicing Factors	*RBM48*	7	92158087	92167319	-	None
ARMC7	Splicing Factors	*ARMC7*	17	73106047	73126360	-	No Entry
PRP3	U4atac/U6atac di-snRNP	*PRPF3*	1	15029392 5	150325671	Autosomal Dominant	RETINITIS PIGMENTOSA 18
PRP4	U4atac/U6atac di-snRNP	*PRPF4*	9	11603762 3	116055185	Autosomal Dominant	RETINITIS PIGMENTOSA 70
PRP31	U4atac/U6atac di-snRNP	*PRPF31*	19	54618837	54635140	Autosomal Dominant	RETINITIS PIGMENTOSA 11
SNU13	U4atac/U6atac di-snRNP	*SNU13*	22	42069934	42086508	-	None
CENATAC	U4atac/U6atac di-snRNP	*CE-TAC*	11	11886885 2	118886501	Autosomal Recessive	MOSAIC VARIEGATED ANEUPLOIDY SYNDROME 4
LSM2	U4atac/U6atac di-snRNP	*LSM2*	6	31765173	31774761	-	None
LSM3	U4atac/U6atac di-snRNP	*LSM3*	3	14219858	14242619	-	None
LSM4	U4atac/U6atac di-snRNP	*LSM4*	19	18417040	18434084	-	None
LSM5	U4atac/U6atac di-snRNP	*LSM5*	7	32524951	32534895	-	None
LSM6	U4atac/U6atac di-snRNP	*LSM6*	4	14709683 7	147121152	-	None
LSM7	U4atac/U6atac di-snRNP	*LSM7*	19	2321516	2328619	-	None
LSM8	U4atac/U6atac di-snRNP	*LSM8*	7	11782408 6	117832878	-	None
U11/U12-20K	U11 snRNP	*ZMAT5*	22	30126945	30163000	-	None
U11/U12-25K	U11 snRNP	*SNRNP25*	16	103010	107669	-	No Entry
U11/U12-35K	U11 snRNP	*SNRNP35*	12	12394218 8	123957701	-	None
U11/U12-48K	U11 snRNP	*SNRNP48*	6	7590432	7612200	-	No Entry
U11/U12-59K	U11 snRNP	*PDCD7*	15	65409717	65426174	-	None
SADI	Tri-snRNP specific	*USP39*	2	85829979	85876403	-	None
SNU66	Tri-snRNP specific	*SART1*	11	65729160	65747299	-	None
PRP4 ki-se	Pre-B specific	*PRP4K*	6	4021534	4065217	-	None

Table S1: minor spliceosome genes [1,2].

Sample	Condition	Sex	Age collected (years)	Age diagnosed with diabetes
T1D1	eT1D control	M	10	58
T1D2	eT1D control	M	5	20
T1D3	eT1D control	F	6	44
T1D4	eT1D control	F	8	56
HEAL1	Healthy control	F	4	-
HEAL2	Healthy control	M	5	-
HEAL3	Healthy control	M	3	-
HEAL4	Healthy control	F	5	-
8	*RNU4ATAC* affected	F	9	26
8 mother	*RNU4ATAC* carrier	M	44	-
8 sibling	*RNU4ATAC* carrier	F	18	
Ci	*RNU6ATAC* affected	M	3	5
C mother	*RNU6ATAC* carrier	F	35	-
C father	*RNU6ATAC* carrier	M	42	-
Ciii	*RNU6ATAC* affected	F	15	260
Cii	*RNU6ATAC* affected	M	8	104
A mother	*RNU6ATAC* carrier	F	27	-
A father	*RNU6ATAC* carrier	M	30	-
11	*RNU4ATAC* affected	F	4	20
11 mother	*RNU4ATAC* carrier	F	29	-
11 father	*RNU4ATAC* carrier	M	40	-
6	*RNU4ATAC* affected	F	11	10
6 mother	*RNU4ATAC* carrier	F	46	-

Table S2: Details of samples used for RNA-seq experiments. eT1D – early-onset type 1 diabetes. M – Male. F – Female.

ID	Status	Age collected (years)
1	*RNU4ATAC*	0.4
2	*RNU4ATAC*	1.6
3	*RNU4ATAC*	0.8
4	*RNU4ATAC*	0.2
5	*RNU4ATAC*	0.8
6	*RNU4ATAC*	0.2
7	*RNU4ATAC*	1.1
8	*RNU4ATAC*	9.7
9	*RNU4ATAC*	0.9
12	*RNU4ATAC*	0.4
Aii	*RNU6ATAC*	0.2
Aii	*RNU6ATAC*	24.3
B	*RNU6ATAC*	0.3
Ci	*RNU6ATAC*	0.1
Cii	*RNU6ATAC*	11.3
Ciii	*RNU6ATAC*	8.7
D	*RNU6ATAC*	5.0
H1	Healthy Control	0.2
H2	Healthy Control	0.3
H3	Healthy Control	0.3
H4	Healthy Control	0.3
H5	Healthy Control	0.7
H6	Healthy Control	0.7
H7	Healthy Control	0.8
H8	Healthy Control	1.3
H9	Healthy Control	1.7
H10	Healthy Control	2.4
H11	Healthy Control	3.3
H12	Healthy Control	4.6
H13	Healthy Control	6.5
H14	Healthy Control	10.2
H15	Healthy Control	12.8
H16	Healthy Control	19.2
H17	Healthy Control	25.7

Table S4: Samples and controls for methylation analysis

Marker (surface)	Fluorochrome	Clone	Company	Panel
Brilliant stain buffer	N/A	N/A	BD Biosciences	All
CD3	APCCy7	OKT3	BioLegend	T cell
CD4	BUV395	SK3	BD Biosciences	T cell
CD25	BB515	2A3	BD Biosciences	T cell
CD25	BB515	M-A251	BD Biosciences	T cell
CD127	APC	A019D5	BioLegend	T cell
CD45RA	BV785	HI100	BioLegend	T cell
CD197/CCR7	BV421	G043H7	BioLegend	T cell
CD183/CXCR3	BV510	G025H7	BioLegend	T cell
CD194/CCR4	BV605	L291H4	BioLegend	T cell
CD196/CCR6	BUV737	11A9	BD Biosciences	T cell
CD95	PE	DX2	BioLegend	T cell
CCR10	PerCP-Cy5.5	1B5	BD Biosciences	T cell
CD185/CXCR5	PE-Cy7	J252D4	BioLegend	T cell
CD278/ICOS	BV711	DX29	BD Biosciences	T cell
CD279/PD-1	PE-Dazzle 594	EH12.2H7	BioLegend	T cell
CD3	APC-Cy7	OKT3	BioLegend	B/DC/Monocyte
CD19	PE	HIB19	BioLegend	B/DC/Monocyte
CD14	PerCP-Cy5.5	HCD14	BioLegend	B/DC/Monocyte
CD16	PE-Cy7	3G8	BioLegend	B/DC/Monocyte
CD56	APC	HCD56	BioLegend	B/DC/Monocyte
HLA-DR	FITC	LN3	BioLegend	B/DC/Monocyte
CD123	BUV395	7G3	BD Biosciences	B/DC/Monocyte
CD11c	BV421	Bu15	BioLegend	B/DC/Monocyte
IgD	BV605	IA6-2	BioLegend	B/DC/Monocyte
CD27	BUV737	L128	BD Biosciences	B/DC/Monocyte
CD38	BV785	HIT2	BioLegend	B/DC/Monocyte
CD24	BV510	ML5	BioLegend	B/DC/Monocyte
CD45	PerCP	HI30	BioLegend	Granulocyte
CD3	APC-Cy7	OKT3	BioLegend	Granulocyte
CD19	APC-Cy7	HIB19	BioLegend	Granulocyte
CD56	APC-Cy7	HCD56	BioLegend	Granulocyte
CD14	AF488	HCD14	BioLegend	Granulocyte
CD15	BV605	W6D3	BioLegend	Granulocyte
CD64	BV421	10.1	BioLegend	Granulocyte
CD63	APC	HC56	BioLegend	Granulocyte
CD123	BUV395	7G3	BD Biosciences	Granulocyte
CD294	PE	BM16	Biolegend	Granulocyte
CD203c	BV510	NP4D6	BioLegend	Granulocyte
CD69	PE-Cy7	FN50	BioLegend	Granulocyte
CD45	PerCP	HI30	BioLegend	Lineage
CD3	APC-Cy7	OKT3	BioLegend	Lineage
CD4	BUV395	SK3	BD Biosciences	Lineage

CD8	BUV737	SK1	BD Biosciences	Lineage
CD19	PE	HIB19	BioLegend	Lineage
CD14	AF488	HCD14	BioLegend	Lineage
CD16	PE-Cy7	3G8	BioLegend	Lineage
CD15	BV605	W6D3	BioLegend	Lineage
CD56	APC	HCD56	BioLegend	Lineage
CD45RA	BV785	HI100	BioLegend	Treg
CD15s	BV510	CSLEX1	BD Biosciences	Treg
CD3	BV605	OKT3	BioLegend	Treg
CD4	BUV395	SK3	BD Biosciences	Treg
CD8	BUV737	SK1	BD Biosciences	Treg
CD25	PE	M-A251	BD Biosciences	Treg
FOXP3	AF647	259D	Beckman Coulter	Treg
Helios	Pacific Blue	22F6	BioLegend	Treg
Ki67	FITC	B56	BD Biosciences	Treg
CD69	PE-Cy7	FN50	BioLegend	Treg

Table S5: Monoclonal antibodies used in the flow cytometry panels

Total B cells	CD45+CD15-CD3-CD19+
IgD+ B cells	CD3-CD19+IgD+
Immature B cells	CD3-CD19+CD27-CD24hiCD38hi
Mature Naïve B cells	CD3-CD19+CD27-CD24+CD38h+
Memory B cells	CD3-CD19+CD27+CD24+CD38lo/-
Plasmablasts/Antibody secreting cells (ASCs)	CD3-CD19+CD27+CD24-CD38+/hi
Natural Killer (NK) cells	CD3CD19-CD14-CD56+
Total Dendritic Cells (DC)	CD3-CD19-CD14-CD16-CD56lo/-HLA-DR+
Myeloid DC	CD3-CD19-CD14-CD16-CD56lo/-HLA-DR+CD11c+CD123-
Plasmacytoid DC	CD3-CD19-CD14-CD16-CD56lo/-HLA-DR+CD11-CD123+
Intermediate monocytes	CD3-CD19-CD56-/loHLA-DR+CD14+CD16+
Classical monocytes	CD3-CD19-CD56-/loHLA-DR+CD14+CD16-
Non classical monocytes	CD3-CD19-CD56-/loHLA-DR+CD14lo/-CD16+
FOXP3+ Tregs	CD3+CD4+CD25+FOXP3+
Activated regulatory T cell (aTreg)	CD3+CD4+CD25+FOXP3hiCD45RAlo/-
Memory Treg (mTreg)	CD3+CD4+CD25+FOXP3+CD45RAlo/-
Resting Treg (rTreg)	CD3+CD4+CD25+FOXP3+ CD45RA+
CD8 T cells	CD45+CD15-CD19-CD56-CD3+CD4-CD8+
CD4 T cells	CD45+CD15-CD19-CD56-CD3+CD+CD8-
Conventional T cell (Tconv)	CD45+CD15-CD19-CD56-CD3+
Central memory T cell (Tcm)	CD3+CD4+CD127+/hiCD25lo/-CCR7+CD45RA-
Terminally differentiated effector memory T cell (Temra)	CD3+CD4+CD127+/hiCD25lo/-CCR7-CD45RA+
T effector memory (Tem)	CD3+CD4+CD127+/hiCD25lo/-CCR7-CD45RA-
Naïve T cell (Tn)	CD3+CD4+CD127+/hiCD25lo/-CD95-CCR7+CD45RAhi
T helper cell type 1 (Th1)	CD3+CD4+CD127+/hiCD25lo/-CCR7varCD45RAvarCXCR5-CCR4-CXCR3+CCR10-CCR6-
T helper cell type 1+ (Th1+)	CD3+CD4+CD127+/hiCD25lo/-CCR7varCD45RAvarCXCR5-CCR4-CXCR3+CCR10-CCR6+
T helper cell type 2 (Th2)	CD3+CD4+CD127+/hiCD25lo/-CCR7varCD45RAvarCXCR5-CCR4+CXCR3-CCR10-CCR6-
T helper cell type 17 (Th17)	CD3+CD4+CD127+/hiCD25lo/-CCR7varCD45RAvarCXCR5-CCR4+CXCR3-CCR10-CCR6+
T helper cell type 22 (Th22)	CD3+CD4+CD127+/hiCD25lo/-CCR7varCD45RAvarCXCR5-CCR4+CXCR3-CCR10+CCR6+
Total Tregs	CD3+CD4+CD127-CD25+
Treg central memory (cm)	CD3+CD4+CD127-CD25+CCR7+CD45RA-
Treg Temra	CD3+CD4+CD127-CD25+CCR7-CD45RA+
Treg effector memory (em)	CD3+CD4+CD127-CD25+CCR7-CD45R-
Naïve Tregs	CD3+CD4+CD127-CD25+CD95-CCR7+CD45RAhi
Treg Th1	CD3+CD4+CD127-CD25l+CCR7varCD45RAvarCXCR5-CCR4-CXCR3+CCR10-CCR6-
Treg Th1+	CD3+CD4+CD127-CD25l+CCR7varCD45RAvarCXCR5-CCR4-CXCR3+CCR10-CCR6+
Treg Th2	CD3+CD4+CD127-CD25l+CCR7varCD45RAvarCXCR5-CCR4+CXCR3-CCR10-CCR6-
Treg Th17	CD3+CD4+CD127-CD25l+CCR7varCD45RAvarCXCR5-CCR4+CXCR3-CCR10-CCR6+

Treg Th22	CD3+CD4+CD127-CD25l+CCR7varCD45RAvarCXCR5-CCR4+CXCR3-CCR10+CCR6+
Eosinophils	CD45+CD3-CD14-CD19-CD56-CD15+CD294+CD203c+
Neutrophils	CD45+CD3-CD14-CD19-CD56-CD15+CD294-CD203clo/-
Basophils	CD45+CD3-CD14-CD19-CD56-CD15-CD123+CD294+
CD69+Tregs	CD3+CD4+CD25+FOXP3+CD69+
CD69+ effector T cells (Teff)	CD3+CD4+CD25-FOXP3-CD69+
CD69+ CD8 T cells	CD3+CD-CD8+CD69+
Proliferating Tregs	CD3+CD4+CD25+FOXP3+Ki67+
Proliferating CD4 Teff	CD3+CD4+CD25-FOXP3- Ki67+
Proliferating CD8 T cells	CD3+CD4+CD25-FOXP3- Ki67+

Table S6: Markers used to define cell populations from flow cytometry

ID	Type	Age collected	Age diagnosed diabetes
HC1	Healthy	11	-
HC2	Healthy	10	-
HC3	Healthy	10	-
HC4	Healthy	11	-
T1D1	Type 1 diabetes	11	11
T1D2	Type 1 diabetes	11	11
T1D3	Type 1 diabetes	11	11
T1D4	Type 1 diabetes	11	11
T1D5	Type 1 diabetes	11	1

Table S7: Controls for flow cytometry

5 MATERIAL AND METHODS

Subjects

The study was conducted in accordance with the Declaration of Helsinki and all subjects, or their parents, gave informed consent for DNA extraction, genetic testing and sample storage in the Genetic Beta Cell Research Bank (https://www.diabetesgenes.org/current-research/genetic-beta-cell-research-bank/). The study was approved by the Wales Research Ethic Committee 5 Bangor (REC 17/WA/0327, IRAS project ID 231760). Individuals with neonatal diabetes (NDM: diagnosed <6 months) or early-onset diabetes (diagnosed <5 years) were recruited by their clinicians to the Exeter Genomics Laboratory for monogenic diabetes genetic testing through a dedicated referral form (https://www.diabetesgenes.org/download/3564/?tmstv=1715331423). Genetic ancestry was assigned using Procrustes analysis and random forest classification [5].

Genetic testing

Whole-genome sequencing of DNA extracted from peripheral blood leukocytes was completed on 181 individuals with NDM and 95 with early-onset diabetes (n=70 with Illumina HiSeq X10 [illumina, USA] n=206 with BGISeq-500 [BGI Europe, Poland]). The resulting sequence reads were aligned to the GRCh38.p14 assembly using BWA MEM version 0.7.15 [6], and processed with our bespoke pipeline based on GATK best practices (Picard version 2.7.1 and GATK version 3.7 [7]). All samples had mean coverage >30x and >95% coverage at >20x. Variants were annotated using Alamut batch standalone version 1.11 (SOPHiA Genetics, Switzerland). We separately analysed the 19,435 coding and 59,251 non-coding genes as annotated in the GENCODE reference [8]. We performed direct Sanger sequencing of *RNU4ATAC* and *RNU6ATAC* following PCR to identify further affected individuals and to perform confirmatory and family member testing. The primer sequences are provided below:

Primer ID	Sequence	Region amplified (Hg38)
RNU6ATAC forward	AGACAGTTCTTCCCGCCTTC	chr9:134164335-134164925
RNU6ATAC reverse	GGGGTGCAGGTTGTAGTGAG	
RNU4ATAC forward	GTGGAGGCTGGAGGTAAGC	chr2:121530763-121531119
RNU4ATAC reverse	GACACTAAAACCACGCGTCTTG	

Islet autoantibody testing

Serum Islet autoantibody testing (GADA, IA-2A, ZnT8A) was performed by Enzyme linked immunosorbent assay (ELISA), at Exeter Clinical Laboratory International (https://www.exeterlaboratory.com/blood-sciences/). This laboratory is UKAS accredited (ISO 15189:2012) and participates in UKNEQAS accreditation and the Islet Autoantibody Standardisation Program.

Transcriptomics

RNA was extracted from whole blood samples preserved in Tempus solution (Applied Biosystems, USA). RNA-seq experiments were performed at the Exeter Sequencing Facility (University of Exeter, UK). Briefly, 100ng RNA was prepared using the Illumina RiboZero library preparation kit to manufacturer's instructions, followed by paired-end 100bp sequencing on an Illumina NovaSeq (Illumina, USA). The resulting reads were then aligned to the GRCh38 reference using STAR v2.7.11b [9]. Transcripts were quantified using RSEM v1.33 [10] and intron retention was measured using SpliceWiz v1.10.1[11]. Weighted gene co-expression analysis was performed using the WGCNA[12] package in R to identify gene modules that were significantly correlated with affected status for minor spliceosomeopathies. Gene ontology [13,14] and KEGG pathway analysis [15] was then performed on the gene sets for these modules using the clusterProfiler package. Differential splicing analysis was performed on the intron retention values from SpliceWiz using EdgeR v4.6.2 [16].

Methylation analysis

To investigate the original immune cell components of cohort whole blood DNA samples we performed methylation array analysis using the EPIC v2 array on an iScan instrument (Illumina, USA). Initial QC was performed to ensure median methylated and unmethylated signal intensities were greater than 1000, that median bisulfite conversion percentage was above 80 and that sample sex determined from methylation data matched reported sex. Normalisation was performed using the Subset-quantile Within Array Normalisation (SWAN) method with the Minfi package [17]. Finally, blood cell deconvolution was performed using the deconvolution tool and optimized blood cell methylation reference libraries from Salas et al.[18]. This method estimates the proportion of cells in the original whole blood from 12 immune cell subsets (neutrophils, eosinophils, basophils, monocytes, naïve and memory B cells, naïve and memory CD4+ and CD8+ T cells, natural killer, and T regulatory cells) using measurement of methylation of cell-type specific loci.

Flow cytometry

Leukocyte populations were characterised from fresh whole blood (collected in 2.7 mL EDTA tubes) using five multi-parameter flow cytometry panels previously validated and assessed for technical reproducibility across multiple laboratories [19-21]. In brief, surface marker staining (Table S5) of 100-200μL well-mixed fresh whole blood was performed for 45 min at RT, followed by red blood cell lysis during 8 min at RT (10x BD FACS lysing solution diluted in ddH2O, BD Biosciences, US). Staining for the lineage flow cytometry panel was conducted on a BD Trucount tube (BD Biosciences, US) enabling the calculation of absolute cell numbers alongside cell frequencies. The lineage tube was vortexed to ensure homogenisation and left on ice until acquisition (lyse, no wash). All other staining panel tubes were centrifuged at 500g for 5 min, cells were washed in 2 mL of FACS buffer [1x PBS (Invitrogen, US) containing 0.2% BSA (Sigma-Aldrich, US) and 2 mM EDTA (Sigma-Aldrich, US)] by centrifugation at 500g for 5 min, resuspended in 200 μL of FACS buffer and kept on ice until acquisition. Intracellular marker staining of 100 μL of fresh whole blood consisted of two 15 min incubations at RT with CD45RA-BV785 and 10 μL of fixative reagent (buffer 1 from PerFix-nc kit, Beckman Coulter, USA), respectively. Permeabilising reagent (buffer 2 from PerFix-nc kit, Beckman Coulter, USA) was added to the intracellular master mix of fluorescently labelled antibodies prepared previously, and cells stained intracellularly for 60 min at RT. After incubation, 3 mL of plain 1x PBS

(Invitrogen, USA) was added for 5 min followed by centrifugation at 500g for 5 min. Cells were washed in 3 mL of 1x R3 reagent (10x buffer 3 diluted in ddH2O from PerFix-nc kit, Beckman Coulter, US), resuspended in 200 μL of 1x R3 reagent and kept on ice until acquisition. Cells were acquired on a BD LSRFortessa™ Cell Analyzer. A minimum of 400,000 events were acquired per sample per staining panel. Phenotypes used to define cell subtypes are provided in table S6. The resulting flow cytometry data was analysed using FlowJo (BD Biosciences, USA) and compared to data from 4 age matched healthy controls and 5 age-matched T1D controls (table S7).

Supplementary References:

1. Bai, R., Yuan, M., Zhang, P., Luo, T., Shi, Y., and Wan, R. (2024). Structural basis of U12-type intron engagement by the fully assembled human minor spliceosome. Science 383, 1245–1252. https://doi.org/10.1126/science.adn7272.

2. Bai, R., Wan, R., Wang, L., Xu, K., Zhang, Q., Lei, J., and Shi, Y. (2021). Structure of the activated human minor spliceosome. Science 371, eabg0879. https://doi.org/10.1126/science.abg0879.

3. Moyer, D.C., Larue, G.E., Hershberger, C.E., Roy, S.W., and Padgett, R.A. (2020). Comprehensive database and evolutionary dynamics of U12-type introns. Nucleic Acids Res 48, 7066–7078. https://doi.org/10.1093/nar/gkaa464.

4. Newman, A.M., Steen, C.B., Liu, C.L., Gentles, A.J., Chaudhuri, A.A., Scherer, F., Khodadoust, M.S., Esfahani, M.S., Luca, B.A., Steiner, D., et al. (2019). Determining cell type abundance and expression from bulk tissues with digital cytometry. Nat Biotechnol 37, 773–782. https://doi.org/10.1038/s41587-019-0114-2.

5. De Franco E, Russ-Silsby J, Batage MH, Thomas L, Wakeling M, Johnson M, et al. Population labels can be generated directly from targeted next-generation sequencing data. 2024; https://doi.org/10.21203/rs.3.rs-5282595/v1

6. Li, H., and Durbin, R. (2009). Fast and accurate short read alignment with Burrows–Wheeler transform. Bioinformatics 25, 1754–1760. https://doi.org/10.1093/bioinformatics/btp324.

7. Auwera G van der, O'Connor BD. Genomics in the cloud : using Docker, GATK, and WDL in Terra. First edition. Sebastopol, CA: O'Reilly Media; 2020.

8. Mudge, J.M., Carbonell-Sala, S., Diekhans, M., Martinez, J.G., Hunt, T., Jungreis, I., Loveland, J.E., Arnan, C., Barnes, I., Bennett, R., et al. (2024). GENCODE 2025: reference gene annotation for human and mouse. Nucleic Acids Res *53*, D966–D975. https://doi.org/10.1093/nar/gkae1078.

9. Dobin, A., Davis, C.A., Schlesinger, F., Drenkow, J., Zaleski, C., Jha, S., Batut, P., Chaisson, M., and Gingeras, T.R. (2013). STAR: ultrafast universal RNA-seq aligner. Bioinformatics 29, 15–21. https://doi.org/10.1093/bioinformatics/bts635.

10. Li, B., and Dewey, C.N. (2011). RSEM: accurate transcript quantification from RNA-Seq data with or without a reference genome. BMC Bioinformatics 12, 323. https://doi.org/10.1186/1471-2105-12-323.

11. Wong, A.C.H., Wong, J.J.-L., Rasko, J.E.J., and Schmitz, U. (2024). SpliceWiz: interactive analysis and visualization of alternative splicing in R. Brief Bioinform 25, bbad468. https://doi.org/10.1093/bib/bbad468.

12. Langfelder, P., and Horvath, S. (2008). WGCNA: an R package for weighted correlation network analysis. BMC Bioinformatics 9, 559. https://doi.org/10.1186/1471-2105-9-559.

13. Ashburner, M., Ball, C.A., Blake, J.A., Botstein, D., Butler, H., Cherry, J.M., Davis, A.P., Dolinski, K., Dwight, S.S., Eppig, J.T., et al. (2000). Gene Ontology: tool for the unification of biology. Nat Genet 25, 25–29. https://doi.org/10.1038/75556.

14. Aleksander, S.A., Balhoff, J., Carbon, S., Cherry, J.M., Drabkin, H.J., Ebert, D., Feuermann, M., Gaudet, P., Harris, N.L., Hill, D.P., et al. (2023). The Gene Ontology knowledgebase in 2023. Genetics 224, iyad031. https://doi.org/10.1093/genetics/iyad031.7

15. Kanehisa, M., and Goto, S. (2000). KEGG: kyoto encyclopedia of genes and genomes. Nucleic Acids Res *28*, 27–30. https://doi.org/10.1093/nar/28.1.27.

16. Chen, Y., Chen, L., Lun, A.T.L., Baldoni, P.L., and Smyth, G.K. (2025). edgeR v4: powerful differential analysis of sequencing data with expanded functionality and improved support for small counts and larger datasets. Nucleic Acids Res 53, gkaf018. https://doi.org/10.1093/nar/gkaf018.

17. Aryee, M.J., Jaffe, A.E., Corrada-Bravo, H., Ladd-Acosta, C., Feinberg, A.P., Hansen, K.D., and Irizarry, R.A. (2014). Minfi: a flexible and comprehensive Bioconductor package for the analysis of Infinium DNA methylation microarrays. Bioinformatics 30, 1363–1369. https://doi.org/10.1093/bioinformatics/btu049.

18. Salas, L.A., Zhang, Z., Koestler, D.C., Butler, R.A., Hansen, H.M., Molinaro, A.M., Wiencke, J.K., Kelsey, K.T., and Christensen, B.C. (2022). Enhanced cell deconvolution of peripheral blood using DNA methylation for high-resolution immune profiling. Nat Commun 13, 761. https://doi.org/10.1038/s41467-021-27864-7.

19. Yang JHM, Ward-Hartstonge KA, Perry DJ, Blanchfield JL, Posgai AL, Wiedeman AE, et al. Guidelines for standardizing T-cell cytometry assays to link biomarkers, mechanisms, and disease outcomes in type 1 diabetes. Eur J Immunol. 2022 Mar;52(3):372–88.

20. Sanz, I., Wei, C., Jenks, S.A., Cashman, K.S., Tipton, C., Woodruff, M.C., Hom, J., and Lee, F.E.-H. (2019). Challenges and Opportunities for Consistent Classification of Human B Cell and Plasma Cell Populations. Front Immunol 10, 2458. https://doi.org/10.3389/fimmu.2019.02458.

21. Yang, J.H.M., Khatri, L., Mickunas, M., Williams, E., Tatovic, D., Alhadj Ali, M., Young, P., Moyle, P., Sahni, V., Wang, R., et al. (2019). Phenotypic Analysis of Human Lymph Nodes in Subjects With New-Onset Type 1 Diabetes and Healthy Individuals by Flow Cytometry. Front Immunol 10, 2547. https://doi.org/10.3389/fimmu.2019.02547.
